# Supplementary material for: Misbehavior or misalignment? Examining the drift towards bureaucratic box-ticking in Competency-Based Medical Education
Source: PLoS One. 2025 Jan 2;20(1):e0313021. doi: 10.1371/journal.pone.0313021 (PMC11694975; doi:10.1371/journal.pone.0313021)
Supplement: S1 File — (PDF) [file pone.0313021.s001.pdf]

Supplementary Material 1

Interview with Residents Guide

Interview starts with going over consent and then handing the resident one or more of their recent assessments.

RA present assessment form and then states, "Please visualize and relive the moment as though it's on video".

"What do you think your preceptor was noticing when they observed you / reviewed you?"
Other related questions that maybe asked to probe further:
"What led to your preceptor's decision / score?"
"What were your preceptor's overriding concerns at that point?"
"How would you summarize the situation at this point?"

"**What information do you think the preceptor used in making this decision?"**

"**What were your and your preceptor's specific goals when using this form?"**
Other related questions that maybe asked to probe further:
"What were you hoping/intending to accomplish at this point?"
"What do you think your preceptor was trying to communicate to the resident?"
"What do you think your preceptor was trying to communicate to program?"
"How did this form help you with your and your preceptor's goals?"
"How did this form interfere with your and your preceptor's goals?"

Continued on next page...

Interview with Preceptors Guide

Interview starts with going over consent and then handing the preceptor one or more of their recent assessments of residents.

"Do you have the form in front of you and if not can you take a moment to look over it?"
"Please visualize and relive the moment you were filling in the form as though it's on video".

"What were you noticing when you were observing or reviewing with the resident?"
Other related questions that maybe asked to probe further:
"What led up to your decision?"
"What were your overriding concerns at that point?"
"How would you summarize the situation at this point?"

"**What information did you use in making this decision?"**
Other related questions that maybe asked to probe further:
"How did you get this information?"
"What knowledge was necessary or helpful in this situation or at this point?"

"**What were your specific goals with this form at this time?"**
Other related questions that maybe asked to probe further:
"What were you hoping/intending to accomplish at this point?"
"What were you trying to communicate to the resident?"
"What were you trying to communicate to program?"
"How did this form help you with that message?"
"How did this form interfere with you providing that message?"
