# Supplementary material for: Misbehavior or misalignment? Examining the drift towards bureaucratic box-ticking in Competency-Based Medical Education
Source: PLoS One. 2025 Jan 2;20(1):e0313021. doi: 10.1371/journal.pone.0313021 (PMC11694975; doi:10.1371/journal.pone.0313021)
Supplement: S2 File — (PDF) [file pone.0313021.s002.pdf]

CD1.85.P
7:40

I This is interview CD1.85.P, and since I don't have your written consent, I'll just get your verbal consent. Do you verbally consent to participate in this study?

R Yes.

I Okay, perfect. My instructions to you is just to visualize and relive the moment as though as it's on video.

R Okay.

I Okay? So, that's just in relation to when you were filling out this form. So, what were you noticing?

R I noticed that the trainee he actually led me through the form, and very easy for me to fill out because he actually filled out the initial more demographic components – the section A completed the resident – so he completed all of those and I thought that was accurate, and then he actually talked me through the very – bits of the form that I would need to complete, and then left me to do that. So, actually interestingly, he initially got up to actually – it seemed like he was he going to leave as I was about to fill out the form, and I then I said, 'Why don't why don't you stay and we'll fill out this together, so I can provide you direct feedback at the moment', which seemed like a very agreeable thing to do.

I Okay, so he kind of led you through that, which made it easier for you, okay. So, what led up to your decision to mark him the way you did?

R I think, actually, we did the form immediately after seeing the patient and having that interaction, and then he had asked me if I would be willing to fill out the form, and I said yes, and then, after he led me through the various areas of the form and completed the bits, I actually had to stop for a moment before I actually filled out my bit, to actually reflect on, well, what was our interaction actually like? So, I don't think I had actually thought about it in the very moment we were actually having the interaction with the patient or reviewing the consult, that I would actually provide feedback or suggest ways to improve on that particular encounter, but actually, by doing the form, that stimulated me to actually reflect more deeply on, well, what was our interaction actually like?, and then actually reflecting on the various things that he did, which most, essentially, were excellent, was able to come up with a few suggestions, and actually, interestingly, he actually, as I was pondering what to provide feedback on for him, he said, 'Sometimes what preceptors will do is bounce it back it the residents – ask them, so what do you think you did well and what do you think you'd like to improve, based on that interaction?', and then so I actually allowed him to speak about what he thought went well, and speak about what he wanted to improve on, based on that interaction, and then, together, we agreed upon what would be suitable things to put in those areas.

I I see, okay, so it gave you a chance to kind of talk about it, even when you weren't reflecting on it, initially, and you worked on it together is what I'm hearing, okay. So, was there any information you used to help make this decision…or how did you get that information?

R Right, I mean, the information that I used was by – this was a indirect observation, so I'd reviewed a consultation with him, and so it was based on my direct interaction with the resident, after we'd reviewed the patient, so that was the information I used. I didn't gather information from anywhere else. I would say, the information, as I said before, though, that I used, was actually the patient's own opinion of what he needed to improve on and what he had done well, so that did factor in.

I Okay, so you talked with the patient?

R Oh, sorry, sorry, not the patient; the resident.

I Oh, the resident, oh, got you.

R I did not elicit feedback from the patient.

I Okay, that makes sense. Okay, and what were your specific goals, at the time?

R That's an interesting thought. My goals at the time of reviewing the consultation with the resident were to provide exceptional care to the patient, and actually it was not focussed on actually improving the resident's ability to be an excellent physician. When I filled out the form, though, certainly my goals switched very much more towards, actually, this being a formative assessment – actually, how do I help this resident be a better physician than he already is? I would say my goals were not summative at all. I didn't feel the need to say this was good or bad or pass or fail or anything like that, but actually to try to find something specific that he could actually modify in his behaviour to be a better physician than he already is. So, my goal was to complete the form – 'cause that's what he asked me to do – and then secondly, to make him a better physician.

I Okay, and how do you think this form helped you with that message?

R I think it definitely helped in the sense that it just provides a stimulus for us to have that feedback conversation. On this particular patient that we saw, it probably would not have given him, actually, any feedback on it. In general, everything actually went very well, but actually by completing the form together, it gave us that stimulus to actually have a feedback conversation.

I Right, okay, so it sounds like it was pretty positive?

R Yeah.

I Was there anything about the form that interfered with you providing that message or that feedback at all?

R No, the form is actually, I think, very nicely constructed. I think the part that would be a minor improvement would be in section b when you're providing essentially, a more global assessment. For example, the highest rating is 'can assess, diagnose and manage this case with no (significant change to approach', then it says you can skip question 7, milestones, if that one's achieved. It would be helpful, in the electronic version of the form, to actually just have those all filled in as – so you don't even have to give it a second though as to whether you really need to fill out that question 7 or not.

I Okay, so I noticed, yeah, you marked him in that fourth box there, so you didn't have to fill out the milestones, but did you have any thoughts on the milestones? Or did you kinda just skip right over that?

R I didn't even look at the milestones after I ticked that box.

I Right, and you didn't have to; I was just curious.

R No.

I Okay, well I think that's all the questions I have for you. Is there anything you wanted to add that we didn't get to?

R No, I don't think so, other than to add that I think, actually, the electronic [cannot hear] of the form, makes it really easy.

I Okay, that's good feedback.

R There were relatively few things to do to actually get to the form, and the form itself is self-explanatory.

I Okay, sounds good.

R Oh, and the one final thing I will add, I really actually liked how the residents are driving these forms forward, and I think that's the way that it has to be, in the sense that it was the resident himself who asked, 'Can you fill out his form me?', and if he hadn't asked, I would not have been pursued to do this form at all. So, especially as he's here doing out of province elective, I was unaware that this was actually the assessment system, whatsoever. So, I think, certainly for out of province, it's very necessary for the residents to drive their assessment forward, and certainly in province as well. I imagine the faculty appreciate it when the resident brings up, rather than to have any goal in remembering to do this.

I Right, okay, well we really appreciate your feedback and taking the time out of your busy to kinda chat with me, and yeah.

R No problem, thanks for the call.

I Thank you so much, take care.

R Okay, thanks, bye.

I Bye

END 07:40

I You said you have the form in front of you. Can you visualize and relive the moment the preceptor was filling in the form, as though it's on video? So, can you describe if this encounter was more about direct observation of your skills or about reviewing your approach to diagnosis and management?

R I saw the patients and then I reviewed with him, and then we went to see the patients again, together, so I think it's more of a direct observation.

I Okay, more of a direct observation, perfect. What do you think your preceptor was noting when they observed you or when they reviewed you, which led to your score?

R When he initially said that I did well on this case. When we saw the patient, he – when I was presenting, actually I said it's hard to assess a wrist effusion, which he comment on later on.

I Sorry, can you repeat that. I'm recording you through the phone, so I just want to make sure that picked up.

R Yeah, actually he asked me to – when I was presenting, I commented on the effusion, that it was very hard to assess, so I'm not sure if there was a wrist effusion or not. So then, when we went to see the patient, actually it was hard for him to assess because the patient was having a tape over her head, and he agreed that we can't see if there is a effusion, and then when we done, he said I did a good job on this visitation, but then his feedback was, 'I thought that you were unsure if there was wrist effusion or not', which we were both unsure if there was or not, actually.

I Okay, so you said that your preceptor said that the patient was kind of hard to assess, and you both weren't sure on the diagnosis?

R Yes.

I Is that right?

R Actually, actually, we aspirated another joint, which was obvious that it was swollen, but that joint specifically that we were asked to assess, it was hard to say if it was swollen or not.

I Okay, so it was kind of a tough case?

R Yes.

I Okay, so what do you think they were noticing when they observed you trying to assess that patient?

R So, I think the most thing is that to be confident and to be sure if there is effusion or not, so probably they wanted more of a commitment to what I saw, and not to be unsure.

I Okay, they wanted more confidence?

R Yes.

I Sorry, I interrupted you there.

R No worries.

I Did you have anything to add to that point?

R No, I think that's it. I totally agree because, this speciality I am on now – like, rheumatology – they care mostly about the joint, so –

I Sorry, can you repeat that?

R So, I'm on rheumatology now, and they care about the joint, so because we are asked to assess the joints, then I should be knowing what I am doing, so, but it was really hard to assess, and he agreed on that, yeah.

I Okay, so you feel like you should know the specialty 'cause that's the area you're in right now, but it was a difficult case, is that right?

R Yes, yes.

I Okay, and you feel like they were kind of hoping that you should be more confident in your assessment?

R Yes.

I Okay, and besides the kind of low confidence that you talked about, what do you think your preceptor's overriding concerns were at that point?

R Like, I won't say low confidence, overall; it's just for this specific thing that I wanted him to assess later on.

I Okay.

R Yeah, so that's why I asked him if he can see it with me, just to confirm if there is effusion or not because I wasn't sure if there was.

I Yeah.

R	Yeah, sorry, what was your question again?

I	I was wondering, what do you think the preceptor's overriding concerns were at that point?

R	To miss effusion, probably later, yeah, ha.

I	Okay, that you missed the diffusion, was that right.

R	Effusion, yeah.

I	Effusion, okay. So next question – what information do you think your preceptor used in making the decision to give you this score?

R	What information?

I	Yeah.

R	Well it's the verbal communication that I had with the preceptor, yeah.

I	Okay, so what you told them? What you communicated with them?

R	Yeah, I told them that I wasn't sure if there was really an effusion there, so yeah.

I	Okay, and do you think your preceptor only used this specific interaction, or do you think they kind of used other observations from that day?

R	Oh no, he showed me how to do it, and he asked me to do it again in front of him. No, he uses direct observation as well, and, I mean, assessment of our clinical skills.

I	Okay, so that was –

R	And he teaches us after that, yeah.

I	Okay, but you think he just kind of looked at the interaction, specifically, versus him watching you throughout the day?

R	Yes, for that case, yeah.

I	Yeah, for the specific interaction?

R	Yes, yes.

I	Okay, and what was your specific goal when getting this form filled out?

R Obviously to get the highest score (laughs), yeah, because, you know , if - I would say this is – now we still have three months to the end of the year, and I'm short on my EPA's, so I wanted to have EPA's done as much as I can, so now I'm asking for EPA even if I – like before, I was asking only if I am confident that I will get the highest score, but now, whatever I'm doing, I would ask for an EPA, just to collect one and also to get feedback, although I was getting feedback in case there was some improvement, but I wasn't asking for an EPA at that time because I was pretty sure that I won't get the highest score, yeah.

I Okay, so initially, you were doing it only when you thought you'd get full marks.

R Yes, yes.

I But now you're trying it out anyways, and also to illicit feedback to help you out?

R Sure.

I Okay, does that sound accurate?

R Yeah.

I Okay, and what do you think your preceptor's goal was when filling out this form?

R To give me a feedback, actually, and to tell me what I should have done to get a full EPA. That means it says that I'm not – I'm not ready now for independent – like, to be independent, seeing this patient, I should have some – like, I should have observation from a well skilled physician, which means that I should have – I should improve myself to be – yeah, to have it done perfectly next time, which I think, if it was – like, if I repeat the same encounter, even now, like, I would do the same. Like, if it was the same the presentation, I wouldn't be sure if there is effusion, and really it didn't change my management because I found another joint which was swollen, so I could have aspirated this joint as well, you know what I meant?

I Um hmm.

R Yeah, so the diagnosis wasn't really based on this joint itself, but that was the joint I missed, yeah.

I Okay.

R Yeah, and also because I didn't do the aspiration of the joint, which I didn't know how to do, so I waited for my self to either to teach me or to just observe me, which is [name]. So, what happened during this case is that I told him that I'm not gonna able to take fluid out of the joint because I didn't do that before, but

that's what, obviously, I would have done, so that was his comment as well – his feedback – that he would give me a full EPA if I have did everything, including the procedure, which I didn't do, which is reasonable, I guess, yeah.

I Okay, and how do you think this form helped you with your and your preceptor's goals?

R I just don't know how the EPA (laughs), to be honest.

I Yeah, you're not sure?

R Well, because sometimes the oral feedback – I'm not talking about this encounter in specific – but sometimes the oral feedback is different from the EPA, so sometimes they would – even when I finished this encounter, he told me, 'Oh, you did a good job.', and then the EPA, the feedback was that there was this thing and this thing to be independent, so it depends on what the EPA looks like and what the preceptor was expecting from a first year resident, so I think it would be different because the EPA – the form – says 'Is this resident ready for independent practice? So, for a staff, like, a first year resident is never ready for independent practice; otherwise, why they are in residency program, right? So, I think that's the point, yeah.

I Okay, and you mentioned – just so I understand correctly, you said sometimes you feel like the oral feedback you receive is a little bit different than what is marked on our EPA form?

R It's usually better because they assess us as a first year resident; while I think the form does – to assess them if we are ready for – to practice independently.

I Are you saying the oral feedback is better than the form?

R Sometimes, yeah.

I Okay, so better, as in they give you more positive feedback – is that what you mean?

R Yes.

I Okay, so even on this one, you said, at the end of it your preceptor said you did a good job?

R Yes.

I How did you feel receiving that oral feedback in comparison to what was marked on the form?

R Like, I wasn’t surprised because I knew I did a good job, as a resident, as what is expected for my level of training, but I totally understand now, when I read the form, that [name] thinks that I need more training to become independent, so yeah, yeah.

I And how did this form interfere with your and your preceptor’s goals?

R Like, it’s very stressful for me (laughs), to be honest, yeah, because now, whatever I am doing, okay, can I get an EPA? And I know it will help me just to do everything perfectly, but that’s what I do in my daily life. Like, I try to do everything perfectly, to be – like, that’s how I would learn; but then now, also I’m thinking, okay, I should get an EPA, so I’m more stressed to do it in a perfect way just to get an EPA, yeah, and I think the preceptor’s don’t have – I didn’t find any preceptor who have any problem filling an EPA, but it won’t be, like, staff, so they will – they would ask us to send the form by email, most of the times, yeah.

I Okay, and then I just have one last question.

R Yes.

I How do you think the preceptor might feel about how filling out this form, overall, helped or hindered teaching and/or patient care?

R (laughs) You know, when I saw the form with this milestone, I said, probably they will give the highest score just in order not to fill the (laughs) – this section (laughs), but then, it depends on the preceptor, I guess, so I think it takes some time for them, although it’s a short form, but it’s also commitment in terms of – because sometimes they send more than one EPA to the same preceptor, so they will need time to fill it up, so yeah, so I’m not sure if they are really happy to do that, or they do it because they have to, yeah.

I Okay, well those are all the questions I have for you today. Did you have anything to add that you didn’t get to speak to?

R No, I think it was, overall, like, I am happy with this evaluation that I got, and yeah, probably next time I will do better, but I hope I will have some time (laughs), yeah.

END 15:02

I And since I don't have your written consent form, can I ask if you verbally consent to participate in this study?

R Um hmm, yeah.

I Okay, sounds good. So this is interview FD1.18.P, and what I'll have you do is just visualize and relive the moment that you were filling out the form, as though it's on video.

R Okay.

I Can you describe if this encounter was more about direct observation of your skills or – oh sorry, I'm reading the wrong section – no, that's right. So, if you can describe if this encounter was more about direct observation of the resident's skills or about reviewing their approach to diagnosis and management?

R It was more review, yeah.

I More reviewing their whole approach to diagnosis and management?

R That's right. I clicked off direct observation, but I guess, yeah, *[preceptor re-reads form aloud]*. I did, so that's direct observation, right?

I Sorry, can you say that again?

R Like, I'm just – like, on the form – the EPA form – I clicked off direct observation 'cause I direct directly observe. Like, it says 'did you hear all of it?' I did. I didn't watch him do any of it.

I Oh, so you didn't see the resident doing anything, but you listened to their presentation?

R Yeah.

I Okay, alright, so I think that would be direct observation of their skills.

R Yeah, I think so too.

I Okay, and what were you noticing when you were observing or review the resident that led to their score?

R I was seeing if they got all the key components of their history, if it was organized, if it led towards a differential diagnosis, and then same with physical exam – if he got all the pertinent negatives and positives, if the findings were

then confirmed when I went to see the patient, and again, if it led to a logical differential.

I Okay, and what led up to your decision to choose the score you did?

R Well, what led to it really was that I agreed with his diagnosis and management. plan and really didn't have to make changes, so basically just what it said there in the last box.

I Okay, was there any other information you used to help you make that score?

R No (laughs).

I Okay, did the resident's performance earlier on have any impact on your score?

R Probably, but I know it shouldn't.

I Okay, can you say a little bit more about that?

R Well, I mean, like, I'm sort of in the – I'm in the process of developing CBD for my specialty, so we've had lots of conversations about this in Ottawa, and for sure studies have shown that prior experiences with a learner influences your decision on, like, your evaluation at any point, but that's not the point of EPA's. It's supposed to be what they – how they performed in that one instant, so anyway, I feel like I did it based on this one case, but probably the fact that he performed well through the rotation made me view his presentation more positively.

I Okay, and what were your specific goals with you filled out this form?

R My goals were to help him complete is EPAs (laughs), and give him one piece of constructive feedback – something that he could work on that was specific.

I Okay, and what were you trying to communicate? What was some of that feedback?

R Well I just wanted to pick one area, based on his case presentation, that I thought he could review more on. So, in this one it was about the indication of – for steroids in HSP was something specific. So, in this case, we did need steroids and he decided we did need steroids, but then I wanted him to tell me when you would use steroids in HSP, which was not relevant to this case because she did need it, so I didn't have to change his management plan, but he didn't know the guidelines around indications for steroids, so that's what I gave him as his feedback.

I And how did this form help you with those goals?

R Well, probably if I didn't – if I specifically wasn't asked to fill out a form like this, then I wouldn't have necessarily gave him a specific task to do - like an area of improvement.

I Okay, so it kind of allowed you – it gave you, like, a starting point to give him feedback – is that right?

R I think it, like, made me do it (laughs).

I Alright, and how might this form interfere with some of those goals?

R I guess, I mean, I feel like in – that the staff didn't understand these assessments very well. They might see a check box on the very right as no need for improvement, since he didn't have to do anything, or look at the other way – that if there is an area of improvement, then they shouldn't get that check box on the right 'cause that's – I think people need to separate, like, the single case versus more global learning issues, but I don't think it hindered me. I kind of understand these assessments fairly well.

I Okay, and how do you think the presence of these new milestones impacted your assessment?

R I didn't even read them.

I Okay, so yeah, 'cause you didn't read them 'cause you didn't have to use them – was that right?

R Yeah.

I Okay, how do you think filling out this form, overall, helped or hindered teaching or patient care?

R I don't think it hindered patient care, and I think it's good for teaching, especially when you don't click the most – like, the no change to management plan. If you click any of the others, it's a really nice way to give constructive because you can point out exactly what you had to change. Like, I really liked, this is not the O-Score, we are using the O-Score in my specialty now, but it's such a nice way to show them that they were lacking in certain areas because you can specifically say, 'Well, I had to change this and this and this, and let's talk about why you missed those things.' So I think doing these forms is a really helpful thing when you're not clicking off that last box.

I So you find it's more helpful if there's more room for that feedback?

R Yeah, I mean, I think when you have to constantly give feedback, the learners will obviously get more feedback. I think, in this specific case, I didn't have to –

like, the form didn't really help me to identify areas of weakness for him because I didn't have to change his management plan much, right.

I Okay, I understand. So that's all the questions I have for you. Is there anything that you wanted to add that you weren't able to?

R No.

I Okay, well thank you so much for taking time to chat with me.

R No problem. I will email you the consent form now, yeah.

I Alright, have a great day.

R Alright, you too, bye.

I Thank you.

END 08:55

FD1.29.P
09:27

I So, this is interview FD1.29.P, and since I don't have your written consent form yet, I'll just ask you do you verbally consent to participate in this study?

R I do.

I Okay, so what I'll have you just to visualize and relive the moment you were filling in the form as though it's on video.

R Okay.

I Alright, so can you just describe if this encounter was more about direct observation of skills or about reviewing approach to diagnosis and management?

R Both.

I Both, okay.

R Yeah.

I So, what were you noticing when you were observing or reviewing with the resident that led to your score?

R So she's on the haematology service, and we see lots and lots of patients who have fever, and this was regarding fevers, and evaluating a patient who has had a new fever. So one of the – so prior to – actually, so the first week on the service – on the haematology service – we talk about an approach to fever in our patients because they're quite complicated, and on top of that they also are very neutropenic, and so they have issues with infections and so on, and so we talk about an approach to this, and the second thing is we do rounds on a fairly frequent basis, and so we go around and we review the patients, and so I had observed [name] a couple of times evaluating these patients on rounds, and so I had a good sense of this, and also I had seen her handle the situation in the past with a few other patients as well. So, the combination of review, teaching in the past for these and then rounds, I was able to directly observe how she approached this particular situation.

I Okay, and what information did you use in making this decision to give this score, if you had other things to add to that?

R There wasn't really anything else. So, the key things that were important was recognizing that patient had a fever, recognizing that it was a new fever in the context of the current antibiotics, recognizing that a source needed to be identified, and recognizing that, even though a patient has been evaluated in the past for a fever, now that there's a new fever, you need to start all over again and work through the entire problem once again. And most importantly, learning what

the management for the patient should be, given this particular context. So, all of those things played a role in deciding the score.

I Okay, and what were your specific goals when you filled out this form?

R Part of it was to convey to [name] that I have confidence in her ability to recognize a fever in a patient – in a haematological patient, to do the assessment for that patient, and then to initiate appropriate management for the patient, so my goal was to convey that information to [name] with the score that she got.

I Okay, and how did this form help you with that goal?

R Pardon?

I How did the form help you with the goal to portray that information and that feedback to the resident?

R Well, I mean, the categories are fairly clear, in that the student does not have an approach, or that they can diagnose and manage the patient case without any significant changes, so it's a nice gradient in terms of the different possibilities for the resident, and in this case it was the – she can manage that particular situation, I think, without much assistance from us.

I Okay, and how did this form interfere with these goals at all?

R It didn't really. I mean, actually this was quite clear. In some of the other forms – for example, there is the evaluation forms that we have for the residents in their mid-term and final evaluations, where there is – I think – I can't remember what the – or I think it's does not expectations and meets expectations, or preceding – progressing as expected, and then the next one is exceptional. I mean, I'm sure there is something in between progressing as expected and ….exceptional, but there's no option for that on that particular form, and so, even though you may want to – a resident is not exceptional but they're certainly more than progressing as expected, there's no room to fill that in. So, with this particular form, though, it seems like that there is a nice gradient between things, and so it's easy to put a resident into a category.

I Okay, so you felt like the categories are a little bit more clear cut, so it was easy to use?

R Yeah, it's just that there's a nicer gradient between the two. It doesn't jump from does not have a basic approach all the way to this person is exceptional. There is other things in between.

I Okay, so I know – I think she scored in the highest one, so the milestones were not used in this case.

R Right.

I But how do you think the presence of these new milestones impacted your assessment?

R It didn't, actually.

I Okay….do you have anything else to add to that or --?

R No, it didn't because when I saw that she can diagnose and she can manage it, then that's what I picked, and then I didn't actually look at the milestones.

I Okay, and then just one last question here --- how do you feel filling out this form, overall, helped or hindered teaching or patient care?

R I don't think it made much of a difference. I don't think it made much of a difference because this is – these situations are very, very specific. So, for example, a fever on a haematology service is not the same as a fever on an internal medicine service or a surgical service. There is very different causes, and there's a different approach to it as well. So, post-surgery, if someone has a fever, then you think about other causes was, and on the haematology service where [name] was, if someone has a fever, then you think of what other causes, and sometimes the two causes have absolutely nothing in common with each other. So, I can say that, in this particular context, [name] did a good job, but if there's another one that – if another patient has a fever on a medicine service, she may not do a good job because she's thinking more about the haematology portion and not the other one, so I don't know if it will make much difference.

I Alright, those are all the questions I have for you. Is there anything you wanted to add that we didn't get to

R No, no, I think these things are good. I like the milestones there, and I think you should still fill them in even if you put someone at the very top, and just make sure that they've been – because they may be able to do some of those things, but then they may have some issues in some of the milestones that they're talking about. Like, if say one of them says "can diagnose and manage this case with no significant changes to initial management plan", but then it says, "seek assistant as needed when unanticipated findings or changing clinical circumstances are encountered", so that may not have happened in this particular case because she's already achieved that, but it might have happened at another similar situation, and even if I had put down can diagnose and manage this case with some changes to initial management, which was the third

one, right, then she may have sought assistance, so I think you should still fill it in.

I Right, okay, yeah, we appreciate that feedback, and I appreciate you getting back to me. I know you're really busy, so thanks – yeah, thanks for participating.

R Oh, no problem.

I And yeah, that's about it.

R Great, thank you.

I Thank you so much; have a good day.

R Okay, bye.

I Bye.

END 09:28

FD1.29.R
08:30

I	And since I don’t have your written consent yet, which you can just send to me whenever in the next week or so – you can even take a picture of it if that’s more convenient for you – I’ll just get your verbal consent today to participate. So, do you consent to participate in the research study?

R	Yup.

I	This is interview FD1.29 R, and what I’ll just have you do is just visualize and relive the moment the preceptor was filling in the form, as though it’s on video. So can you describe if this encounter was more about direct observation of your skills or about reviewing your approach to diagnosis and management?

R	So it was mainly for reviewing the approach. There’s not a lot of opportunities for direct observation on this rotation, so a lot of the stuff is kind of reviewed afterwards, and actually my EPA was filled out afterwards as well, so I didn’t actually have that face-to-face contact when it was being filled out.

I	Okay, that’s good to know then. What do you think – this might be kinda hard to answer if you didn’t observe them filling it out, but maybe just try your best – what do you think they might have been noticing when they observed you or reviewed you, which led to their score?

R	So, it was difficult to say. Actually, I felt like the feedback of this – [name] usually gives really good feedback, but in this particular form he didn’t write a whole lot, so it’s hard for me to say. Sometimes I feel like they think you did a good job, but then they didn’t really reflect a whole lot on what you were saying, and then, as a result, there’s limited comment. And then when you kind of – so, because they have to fill out the milestones now, if they don’t give you a full score, I mean, that actually encourages people to give you the full score just ‘cause they don’t really know what to put for the milestones. That’s kind of how I felt, anyway, about this one.

I	Okay, so what I’m hearing you say is sometimes they might give you the full marks ‘cause they’re not sure what to put down for the milestones?

R	Yeah, then they kind of – they can avoid having to fill that out if they give you the full marks, but I don’t think [name] had a lot of, I guess, constructive feedback to give about this particular presentation. He thought I did an okay job, so there wasn’t that much to say, yeah.

I	Okay, what information do you think your preceptor used in making the decision to give you the score they did?

R	So, I mean, I had this patient for a little bit, so, I think, just knowing, I guess, my daily presentations of the patient were part of my approach. I didn’t actually –

like it wasn't actually one instance where I sat down and was like, this is my specific approach; it was more like, kind of a global impression of how I managed this patient over, like, the course of the two weeks I was with [name].

I Okay, so it wasn't this specific interaction, but kind of, like, overall interactions they've observed you with?

R Um hmm, um hmm, 'cause, I guess, in haematology at least, they're often here for a single issue, and they have other things, but my day-to-day management of the single issue really comes out over the course of the two weeks, rather than a single interaction, I guess.

I Okay, and what was your specific goal when you got this form filled out?

R So, I did want to get more feedback, mainly 'cause, I mean, on this rotation it's pretty busy, so I feel like – and also, because there isn't a lot of opportunities for people to actually review what you're doing, I find, on this rotation, so I just wanted to kind of – not really force, but, like, just give an opportunity for him to actually sit down and give you specific feedback, but [name] has been really good about doing, so I dunno ….

I Okay, and what do you think your preceptor's goals were when filling out this form?

R So, I realize [name] is really good at giving feedback, so he was very proactive about giving us our EPAs, actually, so I think he genuinely wants us to – I guess, for him, I think often the goal is moreso to – he sees that we're doing, I guess, good work, and he wants us – wants this to reflect in our evaluations, so that's why he's really proactive about giving us this EPAs. He does give us feedback on a day-to-day basis, but I think his goal for the EPAs is actually moreso that there is some reflection of the work that we're doing, towards our faculty, I guess, is that makes sense.

I Yeah, totally. How do you think this form helped you with your and preceptor's goals?

R For me, I think I really appreciate knowing that my preceptors are, I guess, thinking about – care about us capturing these EPAs. So, I mean, it lets me know that he is paying attention to my learning needs, so I think that that was – so that was good for me, and obviously [name] accomplished his goal.

I And how do you think this form might have interfered with your and your preceptor's goals at all?

R I think the biggest difficulty – not so much with the specific form; it's moreso that – it's like the way it is with EPA's is that it's difficult to get them filled out on the

spot, which I think, unfortunately, detracts from the point of the EPAs, but it also has been helping me actually get the EPAs – being able to send them afterwards – but then, because I'm sending them afterwards, I find that sometimes the staff don't have time to fill them out right away, and then it kind of defeats the purpose when they're filling out, like, a week later, and they don't really remember exactly what you did, and then it kinda dilutes their feedback. So, not specifically – I would say not specific for this form, but just the EPAs in general.

I Okay, and just the last question here – how do you think the preceptor might feel about how filling out this form, overall, helped or hindered teaching and/or patient care?

R Helped with teaching or patient care, specifically? Or – can you repeat the question? Sorry.

I Yeah, like how do you think your preceptor might feel about filling out this form, overall, and how it might either help or hinder teaching or patient care?

R So, I think he would find that it's helpful, overall, in terms of giving feedback for learners 'cause it's, like, a structured form of giving feedback, rather than – so, I think, like, maybe if there's, like, something constructive or some criticism he wants to give, it's maybe easier to do so through this form, and as, like, a formalized process, rather than him just kinda criticizing my work on a day-to-day basis, so I think that might help with his teaching, and also, in turn, improve patient care, since it improves the work that I'm doing. I think the difficulty would be the time for the hindering of the patient care. I think that, just generally, it's really hard to sit down and really do these forms on a – especially 'cause it's longer now – difficult to them just on the spot if you're, like, busy rounding or something like that.

I Okay, well that's kind of all the questions I have for you. Is there anything you wanted to add that maybe you weren't able to get to?

R No, not really. I think, overall, I feel like I like the milestones that were added. I think it helps guide the feedback that the preceptors are giving 'cause, like, I find that, a lot of times, when they ask for EPAs, they'll look at the box and it's pretty vague, oh, one constructive thing and one thing I did well, I guess, and they kinda struggle to fill that out sometimes if it's a bit vague, so having those milestones helps kinda guide them in terms of what the faculty is looking for from us, so I think that, overall, is an improvement, but I think there's just that difficulty of, I mean, obviously balancing (00:07:41) feedback form, where it's the time that we have to get these filled out because they're already kind of hard to get, so I think it's a bit daunting when they see all these things they have to check off, potentially, but, I mean, hard to say. I dunno yet. I've only had a couple filled out, so….

I	Yeah, and it’s just kind of your perceptions of it, which is what we’re interested in, so yeah, totally. Thank you so much.

END 08:30

I This is interview FD1.52.R, and since I don't have your written consent, which you can just email me after or take a picture and send it to me in the next couple of days, I'll just get you to verbally consent. Do you verbally consent to participate in this study?

R Yes.

I Okay, sounds good. Okay, so I don't know if you remember or have the EPA form that Dr Daniels sent to you. Do you kind of remember it?

R Yeah, I remember what it looks like.

I Sounds good, and I have your form, too, if it helps you clarify anything, but basically, what I want you to do is just to visualize and relive the moment as though it's on video.

R Okay.

I What do you think your preceptor was noticing in that moment?

R Actually, so I used the save for later part of that form because I emailed it to my preceptor because I was on call that night, so I wasn't actually seeing her while she was filling it out – the first one that I did. I actually did another one just yesterday, and I was with my preceptor when we filled that one out; but the first one that we're talking about, I wasn't with her with she did it because we were on call.

I Oooh, okay.

R Yeah.

I Hmm, Dr Daniels sent the first one you did, but you said you did the save for later, which wouldn't be entirely useful. I'm just gonna go off the fly here, and maybe think of the one you did yesterday, and Dr Daniels probably has that on file so he can just go off of that.

R Sure, yeah, so that one, I was doing it with an emergency room doctor, actually, because that's the rotation I'm on, so they had – he had never seen these forms before, they were totally new. So, I was walking him through kinda the whole thing, start to finish. I ended up being pretty clear what he was supposed to do in each box as we went through, but I think, judging by, kind of, his reaction, that, once we got to the milestones part of it and we were trying to go through that, it was a little bit overwhelming to him how many boxes there were to fill out.

I Okay, so it was a new form. They didn't really know about it, but when they got to the milestones, it was kind of, like, 'whoa, there's a lot of stuff to fill out here'.

R Yeah, exactly.

I Okay, how did you get that sense?

R He just was kinda like looking it, and scrolling kinda back and forth and up and down, and he's kinda like, you know, what is all of this stuff kind of reaction, just being a little bit overwhelmed by how much there was.

I Okay, and what do you think led him to his decision or his score?

R I think – he didn't verbalize it, but, like it says clearly in the fourth box that you don't that you don't have to fill out the bottom part if you clicked that, so I wouldn't be surprised if that was some kind of influence, but I think the biggest part was I sat down and explained to him, kind of, as we had explained the different ranking system to those – like, the boxes there – the first part – the part before milestones, just that the fourth one is – there can be some changes, but it shouldn't be a significant change to the management plan or to what we're doing. It shouldn't be something that would kinda be like a deal breaker in moving the patient forward, safely, and I think that explanation seemed to be the biggest part that helped him get his score.

I Okay, just to clarify, did you score in that fourth box where you didn't need to do the milestones?

R I did score in the fourth (highest) box.

I Okay, so you got the fourth box, so he didn't fill it out, but he was kind of just reacting to it?

R Yeah, exactly. He went kinda scrolling through to see what there would be to do for the form, and he reacted to it, but he didn't actually have to fill it out, in the end, yeah.

I Okay, do you feel like – I know you kind of briefly touched on this – but do you feel like there was a sense of 'there's a lot of stuff; maybe I should just kinda click that last one to avoid that'?

R Yeah, I mean, I couldn't say, for sure, but I think, at first, he was kind of looking at the third box, and then he was looking at the fourth. But, at first, he kinda looked at it, and then it was kinda like look down at the rest of it, and then he became a little bit – like, to me, possibly a little bit excited when he saw what else there was to fill.

I Okay, yeah, and it's not – for sure, the whole point of this is to get a sense of what you were thinking and what you were kind of noticing.

R Yeah.

I And it's just all your perception, but all that kind of just helps us. Okay, awesome. What information do you think our preceptor used to make his decision?

R I think – about what – like, from that 1,2,3,4, across the top?

I Yeah, like his overall decision – decisions, based on what he filled out on the form, just overall.

R Yeah, I think – I mean, like, obviously a lot of it's based on – it was emerg, so it was an interaction a little bit earlier on the day 'cause we did at the end of the shift, and based on that kind of specific case we were going through, I am guessing that probably it's also an overall impression of me from the entire shift and not just the one case 'cause I think that would be hard to separate, and we kind of talked about the overall shift a little bit too, and then in terms of deciding how I fell – like, my performance fell within that ranking, after he'd reflected on that, I think it was definitely influenced by my explanation of what each box means, since she didn't really have that background.

I Okay, so kind of like a summative assessment of the day, and also in collaboration with you?

R Yeah

I Okay, cool. What do you think your preceptor's specific goals were at the time?

R So, for – it might be a little bit different for the emerg shift, but I was supposed to have some kind of evaluation at the end of the shift, which is not entirely keeping with the EPA – like, the kind of point evaluation – and so this response is a little bit of a surrogate, and say I had been there and give me some kind of feedback and evaluation. So, I think it was the combination of kind of a summary of the whole shift, in total, but also finding something to tell me, specifically, to kind of work on, going forward. He gave me a little bit of reading tips and that kinda thing, and to give me feedback or organization and, yeah.

I How do you think the form helped you with that goal, and your preceptor's goals?

R I didn't have the greatest kinda organization to get feedback, back. Like, when we got to the end – the kind of one comment to improve on – he was kinda stuck on that for a while, and judging by that, I was feeling he might not have given me a lot of feedback to improve on if he had just talked to me about the shift, in

general, but I think it helped him target an area of improvement for me, based on that one case, possibly.

I And how do you think the form interfered with your or your preceptor's goals, if it did?

R I don't know that it. I think, at the end, I think, if anything, it – in theory, it might have stopped us from talking about this shift, kind of as a whole, and anything that didn't necessarily relate to the case that I needed to work on, but at the end, we still kinda summarized everything else, so I don't think it really inter – yeah.

I Okay, yeah, sounds good, so it didn't really didn't interfere 'cause you kind of talked about the whole shift, but you could see how maybe you would focus on that one specific case.

R Yeah, exactly.

I Okay, well I think that's all the questions I have for you. Is there anything else that you wanted add that we didn't get to?

R No, no, I think that's everything, yeah.

I Okay, I appreciate you taking time out of your busy day to chat with me, and yeah, we appreciate your feedback on this, and we hope we can use it to kind of help you guys out.

R Oh, thanks.

I Okay, awesome, well have a good day, and if you have time, just send over the consent form to me via email or just take a picture of it, and you can send it to me sometime within the next few days or with the week there.

R Yeah, perfect, will do.

END 09:11

I This is interview FD1.64.P. What I'll get you do is just to visualize and relive the moment as though it's on video.

R Okay.

I In that moment, what were you noticing?

R So, do you mean when I actually filled out the form itself?

I Yes.

R I mean, it seemed to be pretty self-explanatory. It was the first time I'd actually filled out the form, but I thought it was – you know, I just sort of went through it, section by section, and it seemed to be pretty self- explanatory.

I Okay, and what led up to your decision?

R So, we had done a consult on the ward. It was a weekend, so this was actually my first time working with [name], but we had a new consult on the ward that she sorta saw ahead of myself and the staff physician as well, so she went ahead and did it. She did the – she looked ahead on Netcare (our EHR), and then she assessed the patient, and she sorta came up with her plan, what she thought was going on, and where we should go from there, and then we went back in together and sort of clarified anything that needed clarifying, and then I examined the patient, and we sort of discussed our plan from there.

I Okay, and what information did you use to make your decision?

R So, basically, I used just the information because I only had the information from that day, so I used the information, essentially, from that encounter.

I Okay, that specific case?

R That specific case, yeah….yes.

I Okay, and how did you get that information?

R Sorry, what do you mean? I'm not sure I know what you mean. Like, I got the information from her, I guess, is that what you mean?

I Yeah, like, were you watching her, or did you just talk with her? How did you come to mark her the way you did, I guess? How did you make that choice?

R Yeah, so I didn't observe her doing her actual history and physical and that sort of thing, so I did it based on her presentation to me, and then going back in and

clarifying things with the patient, just making sure everything was accurate, and then examining him myself as well. So, I guess, just sort of confirming things that way.

I Okay, so talking with the resident and then talking with the patient afterwards, too.

R Yeah.

I Okay, and what were your specific goals at the time?

R My specific goals with respect to my interaction with the resident, is that what you mean?

I Yes.

R I guess my specific goals were just to ensure that the information that we had was accurate, that the physical exam was accurate, that if she had any questions or things that she wanted to me to clarify that, you know, she was unsure about with the physical exam, that we had done that, and then that we had just sort of discussed through the case with any pertinent things that I though were relevant, that, you know, things she can she could improve on or things, maybe, that she had missed and that sort of thing.

I Okay, and how did this form help you with that message?

R I'm not sure that it really helped me with that part of it because I actually did it afterwards, when the entire encounter was over, so I'm not sure that it sort of changed anything from what I had done. I dunno, perhaps, yeah..

I 'Cause you did the save for later, right?

R Yes, I did, yeah.

I Okay, so that wouldn't really apply, you feel like?

R Um hmm.

I My next question was gonna be how do you think this form interferes with you providing that message, but I'm sensing you might not have anything to speak towards that either.

R No, I guess 'cause I did it retrospectively, and, I mean, I guess that's a good question is that, is this usually done sort of with the resident present, and sort of discuss it as you're going, or are most people doing it afterwards or -- ? I'm not sure.

I Right, yeah, okay, and I know you didn't fill out the milestones because you scored her in that fourth box there, but did you have any thoughts on that?

R Okay, so I have this pulled up.

I Oh, actually you did score her in that, sorry. Do you have that form in from of you?

R I do, yeah. So, I thought, you know, certainly there was room to improve when it came to sort of differential diagnosis and those sort of things, but when it came to the actual assessment that she did, I thought that, you know, she did well with that, so yeah.

I Okay, well that's all the questions I have for you. Is there anything else that you wanted to add that maybe we didn't touch on?

R I don't think so, no. I mean, I guess, I dunno if – I know 'cause I know this is part of the CBD and that sort of thing, so I don't know if – I think maybe people are getting more training on exactly – a little bit more information about the forms, so this was my first time doing it, so I dunno, I guess feedback, for me, on whether I was sort of in the appropriate ballpark with my evaluations would be – I mean, when I thought about it, what I was – I'm an R5, so I was sort of thinking about where I should be on the form and where she should be on the form, as an R1, sort of, you know, but probably a little bit more education or information about this, I think, for – even for senior residents would be helpful.

I Okay, yeah, and part of our process is just to see how you're filling them out, and that process kind of helps inform how we implement it too, I guess.

R Um hmm.

I It's helpful to kind of hear your thoughts on it. I'm hearing you say you don't really – you might not have a lot of experience with it, and maybe you're comparing where the resident should be, based on your own experience.

R Right.

I Was there anything else in terms of filling out the milestones, how you approached it?

R I don't think so. I think, you know, I thought it was an easier form to fill out than some of the previous ones we've had in the past, I think, as far as evaluations go, yeah.

FD1.64.P
07:16

I Okay, alright, sounds good. Thank you so much for your feedback today. We appreciate you taking time out of your busy day to chat with me.

R No worries, and I'll just send you that consent form right away.

I Okay, perfect.

R Thank you.

I Alright, have a good day, bye [name]

R Okay, thanks, bye bye

END 07:17

Interview FD1.64.R

Interviewer (I): Just before we start, can I just get verbal consent from you to participate in the interview?

Participant (P): I agree.

I: Ok. Perfect, and then you can just send me the PDF or like take a picture of the PDF signed and email it to me within like, the next week.

P: Ok sounds good.

I: Ok. So, so, my instruction to you is if you can visualize and relive the moment as though it's on video.

P: Ok.

I: Ok? So kind of visualizing when you did this form with your preceptor, what do you think your preceptor was noticing?

P: So I think my preceptor read the form pretty carefully, she- I find that actually a lot of people skip the overall evaluation, and she went straight to the section that broke down like, the different aspects I guess of the evaluation. and ya, and I think she was I guess compa-- like so I did it with the fellow so I think she was sort of reading it and comparing it in her head to her own evaluations.

Phone call is cut off

I: Hello?

P: Hi [Interviewer]. Sorry. I think I went to somewhere that didn't have good reception so--

I: Ok, no worries. Thanks for calling me back.

P: No problem.

I: Ok. We can just pick up where we left off. I think you were saying she skips to kind of, the breakdown of the form.

P: Yah like the big chart where it lists individual things. That was more eye-catching. She immediately started filling that out

I: Ok and you were saying something about her comparing to something else? I missed that part.

Oh I just think she like, as a fellow she probably has some evaluations that she was saying like "I don't expect you to be that level. I think this is where I should be or you know competency level."

I: Ok, so what do you think led her to the decision or the score that she marked?

P: I think probably just the phrasing of the the questions. Like I think some of the – I can't remember exactly. Do you have a copy of the form in front of you?

I: Ya. I do.

P: Ya. I think the scoring, like it's um- what is the scoring for the competency again?

I: Yup. I'll pull it up. I actually have yours that I can just pull up. so the milestones so for example, there's 'recognize urgent problems that need the involvement of more experienced colleagues.' That one you were in 'achieved' and 'perform complete appropriate assessment of patient,' you were in 'achieved', and then the only two where that were kind of 'in progress or area on which to focus' was' generate differential diagnosis along with appropriate diagnostic strategies' as well as 'develop and implement initial management plans'.

P: Ya so I think for the two that she was commenting on, she like would expect that as a like a year five fellow finishing her fellowship she would expect herself to achieve these goals and not like a person on my level.

I: Ok so she was kind of taking a developmental approach almost.

P: Ya.

I: Ok and what information do you think the preceptor used in making this decision?

P: so I like did my presentation directly to her and then I also let her know I had I did my presentation. And then we broke ,we saw the patient and then we came back, and then before we discussed the management plan I sort of let her know that I was hoping to get an EPA done and if I could just like, present my management plan and differential diagnosis for sake of the evaluation. So she sort of heard the whole story and then so she was basing it off of that, and then I guess probably comparisons to like other people in R1.

I: Ok.

P: And where she thinks I should be at. No actually sorry, let me back up. That's not right.

I: Sure.

P: Probably comparisons to like how she would manage the patient or how the staff decides to manage the patient.

I: Ok and what do you think some of her maybe overriding concerns were at that point if there were any?

P: I think at that point- so my differential, my main differential was correct but the secondary ones were not. So we- and then we discussed why she didn't think like- I said the differential was mixed connective tissue disease and she didn't really think so. She thought it was dermatomyositis so we kind of discussed what mixed connective tissue disease was and why we didn't think it was.

I: Ok

P: and then for management… sorry I'm just trying to remember she, for management she just wanted to hold off and do some more blood work and confirm the diagnosis over [cannot hear] before starting the patient on prednisone and plaquenil because he was currently stable. And whereas I wanted to started him right away. And some of the blood work differed a little bit.

I: Ok so you kind of talked about it afterwards.

P: Yeah.

I: And how do you think this form helped with you and your preceptor's goals?

P: so I think at least in the form, although it seems like for a subspecialty like rheumatology. It's pretty like sort of I'm expected for an R1 to sort of achieve everyone in that, especially for more complicated case like SLE, but I thought it was good in that it sort of gave me the opening to say, "Ok here's my plan, assessment plan, could you assess this for evaluation?" Because sometimes I feel for some subspecialty special services they listen to your history and physicals and just kind of don't, they just kind of go off of the plan. They don't spend time on it or they just go off because they don't expect you to make that kind of steps.

I: Ok so it gave you kind of a point to start the conversation.

P: Ya.

I: Ok. And on the flip side of that, how did this form maybe interfere with your and your preceptors goals?

P: I'm not sure if it interfered but I mean for my goal, I guess if my goal is to get these evaluations that like a certain number of evaluations I achieve everything, I think it's hard to get good evaluations on subspecialities for complicated cases.

I: Mm-hmm.

P: so it might be hard to miss my benchmark- or make my benchmark sorry.

I: Right.

P: Ya, for a preceptor, I don't think if it inhibits, maybe it just take a bit of extra time. Maybe it might be like repetitive, like mention things before. I don't know, I feel like they're there to teach and give feedback so I think it's a good way for them to do so.

I: Ok. Well that's all the questions I have but is there anything else you wanted to add that we didn't cover?

P: Uh no. I think that's good.

I: Ok, well thank you so much for your time, we'll send you a coffee card at the end of the study but other than that, that's all I need from you.

P: Great, thank you.

I: Thank you so much for touching base.

P: Sorry about the multiple delays.

I: No worries I'm glad we made it work.

P: Ok.

I: Ok. Take care.

P: Take care.

I: Bye.

P: Bye.

I So, this is interview FD1.98.R, and since I don't have your written consent, can I just get your verbal consent to participate in this study? Do you consent?

R Yes, yeah.

I So, just visualize and relive the moment the preceptor was filling in the form, as thought it's on video.

R Okay.

I Can you describe if this encounter was more about direct observation of your skills or about reviewing your approach to diagnosis and management?

R Reviewing, I think, my approach to the diagnosis of this case, yeah.

I What do you think your preceptor was noticing when they observed your or when they reviewed you?

R I think, when he was reviewing the case, he was going over how detailed I was, in terms of taking the history, and whether or not I got all of the pertinent facts. I think he was also reviewing my knowledge base as well because I think it was a very complex sort of process, and he was trying to factor in multiple things at the same time, yeah, if that makes sense, yeah.

I Yeah, what do you think their overriding concerns were at that point?

R I think, for each preceptor, the way that they prefer sort of things to be done or they prefer reviewing cases is they either like a very detailed approach or sort of a more pared down focussed approach, and I think that's what he was really looking for in that scenario, and that was sort of the feedback that he gave me is to sort of find out what the relevant details are, and be able to organize it in a matter that's understandable easily, and I think that's what most preceptors are looking for at this level of training. Especially as an R1, you have to prove that you know a lot of different things, but at the same time, you have to know which details are relevant to the preceptor and to the person listening, um hmm.

I Okay, so you kind of alluded to this a little bit, but I'm wondering if you can tell me a little bit more about what information you think your preceptor used in making the decision to give you the score that you received.

R What information my preceptor used?

I Um hmm.

R Okay, so I remember I had reviewed – so I presented this case with him – to him – and then he went back and saw the patient, so he probably was verifying my history and physical when he went back and saw the patient with me. Sorry, the question was what things he was looking for?

I What information do you think he used to kind of assess you to give you the score you did?

R Yes, yeah, yeah, yeah, so I think that was the one thing. I think he used his knowledge of, obviously, that case. It was – I think it was inflammation of the bowel, and I think, yeah, he probably used how thorough he thought my differential was, and I think he compared his knowledge to mine, obviously, just to make that decision. And in terms of the management plan as well, I think he was reviewing how correct I was in my management, and yeah, and making sure that I got all of the relevant details. Yeah, for patient safety and for comprehensiveness of the diagnosis, and then for the correct treatment. Oh sorry, I hope I wasn't rambling too much (laughs).

I No, don't worry about it. I think anything you say is data for us, so feel free to ramble away.

R Okay.

I Do you think your preceptor just used this interaction with you, or do you think they used observations from that day?

R I think – that's a good question. Obviously, my preceptor had – like I've only worked with him on call, so I think he could only use his observations from that day, really, just in that particular assessment, yeah. It's sort of been difficult to find preceptors who have been with you for, like, two weeks, who are willing to do assessments so far. I think it's – you just have to find out whoever is available and whoever is willing, sort of, and it's kind of sporadic, so yeah, in this case it was really, like, that – just that one time, and he didn't see me for very much, so.

I So, just to clarify, for this EPA form, you were thinking it was only about that specific interaction?

R Yeah, absolutely, because I didn't see him before that or, yeah, or at all.

I Okay, I see. What was your specific goal when getting this form filled out?

R Specific goal? So, obviously I was looking to complete the EPA (laughs), I think. That was, like, the primary objective. We need so many of these filled out, and yeah, but sorry, probably, like, way too obvious of an answer. The second thing, I think, was really looking for feedback. Sometimes I really think, having these EP's is helpful because it provides an opportunity for you to gather information

about how you're doing in an otherwise quite very busy scenario. So, the feedback I was looking for was about my – like, the way that I was taking the histories and physicals, and more specifically about my management plan. I think history and physicals, like, we're – I don't mean to say that residents are perfect at them, but I think it really – you really have to focus more on the management aspect of things, and so I think I was really looking at feedback in terms of what I should have done differently, or what I did well in terms of the treatment idea that I had for this case. Unfortunately, I don't think that was really the feedback that I received. I think maybe – I don't know if it was, like, the design of the EPA of if it was just the case that we went over together, but I think he was more sort of interested in giving me feedback about the history that I took and sort of the physical exam, and whether or not he agreed with it, so but I'm sure it varies, yeah, but yeah.

I Okay, so you were hoping to get feedback.

R Yeah.

I And you did get feedback, but it wasn't specific to what you were hoping – is that right?

R Yeah, yeah, yeah, I would say so, yeah.

I Okay, and what do you think your preceptor's goal was when filling out this form?

R He probably wanted to do the – well, I think I sort of explained it as well. Like, I think he thought it was an opportunity to give feedback on the resident's sort of approach to this case, and sort of review what he wants done different for the next time when he's on call with me or with a resident. He was really looking for – to sort of specifically tell me what he wants in terms of a case presentation, which, yeah, I think it's very – yeah, he wanted a more focused, pertinent detailed history. Some people really want a more detailed – they want everything in the picture, and they want to know everything from, like, the patient's social to, like, family history, and where, if he wanted to give me feedback, but that's not what he wanted. Yeah, so I think that was relevant to this case. Sorry, I'm trying to answer questions so that it is pertinent to, yeah, this scenario, yeah, yeah.

I Okay, how do you think this form helped you with your and your preceptor's goals?

R It definitely helped in trying to achieve them. I think it – again, as I said before, it's a great opportunity to just sit down and sort of reflect on how things went, and I think, yeah, it was just a good opportunity for us to take a step back an figure out what we can do better , and what went well, and that's always a great idea, especially – yeah, especially for the learner and for the, I think, my preceptor, just so that – yeah, just so that they – yeah, yeah, yeah.

I Sorry, I cut you off – just so that they –

R I don't know what word I'm looking for. So that they know what to do better next time, yeah.

I Okay, so just feedback for both of you, then?

R Yes.

I Okay, perfect. And on the flipside of that, how do you think this for maybe interfered with your and your preceptor's goals?

R Oh, yeah, the layout of it was he was in a rush – sorry, I don't mean to say that we were – like, he was too busy or anything. I think he really took the time to give me personal feedback, which was great, but I think, you know, there's lots of milestones on that form – there's like a big table and lots of things to click through. I think, before it was sort of easy to make that judgement if there was only that one scale with the four, and you could just click on one of them, but I think the – like, having so many options, as well, made it sort of prohibitive because he had to read through all of the options, and then decide – like, make that judgement about where he thought I stood, and I think may have been prohibitive, in that sense, because I could tell that he was, 'Ah, you know, ah you're okay; let's just give you this one', and then, you know, 'I don't really want to go into too much detail because --- ',and I have to say that I kind of agreed as well. I think these EPA's – you know, this – I think, if they're fast and that people are able to do them fast, it'd be good because then you can sort of cut down on the time that you need to do them, and there's so many, so yeah, I remember when he sort of – like, I think we both, like, looked at all of the milestones, and we were kind of like, 'Eh, you know, we can go over this later and maybe with a different case', yeah.

I Hmm.

R Yeah, yeah.

I So, you were saying just at the end there, 'looking at it and feeling like we could go over it later.' Can you speak a little bit more to that?

R Yeah, I think he said – or not he said, but I think maybe we were sort of both thinking that, you know, 'You're not at the level that this applies to you in terms of – ' – 'cause he obviously chose the option where he thinks I could manage this, yeah, at the very – like, I think it was the last one. I think he said, oh, if he really thought I needed some help, then he could probably go over it later and in more specific detail – like, later on, yeah, in a different sort of case, yeah.

I Okay, and I just have one more question for you. How do you think the preceptor might feel about how filling out this form, overall, helped or hindered their teaching or their patient care?

R How the preceptor might feel?

I Yeah.

R Ha, I think, on the one hand, I think he saw it as a good learning opportunity for the both of us. I think he wanted – you know, yeah, he likes giving feedback and stuff like that. I think, on the other hand, he may have – you know, he had to pull me out of rounds to actually give me feedback on this, and he had to take time out of his day as well, so I think it was sort of, like, recognizing that we both need to set aside time to do this, which is difficult sometimes because people don't actually find that that's important, and then that we both thought it was a good – like it's a good thing to do – like it's meaningful. It's just, yeah, we just need to find the initiative, to find the time to do it. It was actually difficult getting to this point and getting this eval because, you know, we'll have to wait until we found some time where we could meet up, and then he was a preceptor for a different team, so that was more difficult, and stuff like that, if that makes sense. I think I'm rambling at this point.

I I think I get you. It takes some time, and you have to both kind of match up your schedules and –

R Yeah, yeah.

I -- it's just a bit difficult, okay.

R Yeah.

I Well that's all the questions I have for you. Is there anything that you wanted to add that maybe you weren't able to add in there?

R No, I think we've pretty much covered everything, yeah.

I Yeah, I think you did pretty good for someone who said you didn't have much to say.

R Oh, thank you. I'm post-call, so I'm, yeah, a little bit delirious as well, so hopefully I made sense.

I Yeah, we definitely appreciate your feedback. I know you're busy and tired and stuff, so we appreciate it, and we hope we can use this to kinda further help you guys.

R	Thank you so much for your help, yes, and I appreciate all the research that goes into this, so hopefully it works out well in the end.

I	Yeah, thank you. So, just on your part, all I need from you is, just within the next week or so, just email me the consent for, and I will email you a coffee card when this is all over, but that's all I need from you.

R	Okay, great, I will try to get on that.

END 15:57

I Since I don't have your written consent, can I just get you to verbally consent to participate in this interview?

R Yes.

I This is interview CD1.06.P, and what I'll have you do is, either looking at the form or thinking back to the form, can you just relive the moment you were filling out the form? So I'll just have you kind of think of that.

R Sure, yeah, I've got the form in front of me here.

I Yeah, just kind of like, basically, go back to that moment is kind of what I tell people when we start. Can you describe if this encounter was more about direct observation of skills or about reviewing approach to diagnosis and management?

R So I think it was probably both. This was an inpatient review of a case, so the resident presented the case; we reviewed kind of a diagnostic approach, and then actually went and she directly was interacting with the patient and I was observing.

I Okay, and what were you noticing when you were observing or reviewing with the resident that led to your score?

R So, I think she had a really good approach, but certainly was missing a lot of pieces and needed a lot of direction, and even after kind of a full review prior to going into the patient, I still had to correct some things that she had said to the patient, so that part of things, I think, you know, it shows that she's got some of the knowledge but wasn't really absorbing all of it.

I Um hmm, and what were some of your overriding concerns at that point?

R I guess just whether or not I had kind of clearly told her what I had wanted her to do because she didn't fully, I think, understand the case, and whether or not that was something that I had missed or hadn't clearly communicated to her, or if she just hadn't really grasped that.

I Um hmm, what information did you use in making this decision to give the score?

R So, I guess, basically, just the direct observation that I had with her, that I had certain expectations that she did meet and others that she didn't meet.

I Okay, so you were kind of just observing her, and if she kind of met the criteria to what you kind of set in your mind, then giving her the score you did?

R Um hmm, yeah.

I And did her performance earlier on have any impact on her score?

R To be honest, this was pretty early on, and this specific case was quite early on in my assessment of her, and, if anything, I think was probably one of the weakest cases that she had reviewed with me.

I Okay, so just to clarify, did your prior knowledge of her, working with her –

R Not really, no, I wouldn't think so, no.

I Okay, so it was kind of solely based on this one presentation?

R Um hmm.

I And what were your specific goals when you filled out this form?

R I think – I guess just communicating that I don't think she's quite at the level to be independent, for this specific presentation, and from this specific encounter is that she's not quite hit those milestones yet.

I Okay, and how well do you think the milestones help you communicate what you wanted to the resident in progressing in the program and what they need to do to progress?

R I think it's useful as kind of identifying which areas need a bit more work, but I think, I guess, the comments is still the most useful for directed kind of recommendations on how to improve.

I So it's kind of like a good little checkbox to identify what the resident needs to work on, but maybe not as valuable as kind of that qualitative feedback?

R Yeah, I think most – and at least in my experience, most residents need a very specific feedback, and really, it's gonna be case dependent and presentation dependent. So, you know, identify that they have a problem with differential diagnosis doesn't tell them 'these are the four other things that you should have actually thought about', so I think that kind of gives you a category with the milestones, and then the comment section is really to give them the specifics of what they need to work on.

I Right, how did this form help you with your goals in communicating with the resident?

R I think it's useful in kind of identifying which areas they do need to work on and which areas they have done a good job on. Yeah, so I think it's nice to kinda

break it up, rather than just kind of an overall score as some of the previous evaluations were.

I And how might this form interfere with some of your goals?

R Not this specific one, but I think, when the form says that basically they've done a very good job, you can basically skip all of the milestones and say they have achieved everything, I think that is a bit of a limiting factor because you can say that they've done everything correctly, but most residents are still gonna have some that they can work on or improve, so I think that – you know, when you hit that one, it seems like you shouldn't be writing in the comments that anything should be improved. If you've said that they've essentially achieved those milestones, then really it means that they don't really have much more to work on.

I Is that kind of something you've experienced or observed – where you kind of say they're doing really great, but still write something in terms of feedback?

R Yeah, and I do try and still write feedback anyways 'cause I think the residents do read that and take it into account, but yeah, sometimes, I guess, there's a little bit of grey area even when they have achieved those milestones.

I Okay, and then I just have one last question. How do you feel filling out this form, overall, helped or hindered teaching or patient care?

R I think it helps teaching 'cause I do think it's a bit more specific for the residents. I don't really think it changes patient care at all, yeah.

I Well that's all I have for you today. Is there anything you wanted to add about the form or your experience?

R No.

I Okay perfect, well I appreciate you taking time out of your day to chat with me and to participate in the research, and yeah, just send me the consent form whenever you have a chance, and we will send you the gift card when the study is completed.

END 07:29

I This is interview CD1.11.P, and [name], do you consent to participate in the research?

R Yes.

I Just looking at the form in front of you, what I'll have you do is just to relive the moment you were filling in the form, and if you can describe if this encounter was more about direct observation of skills or about reviewing approach to diagnosis and management?

R From what I recall, it was more a review.

I What were you noticing when you were observing or reviewing with the resident that led to your score?

R In terms of what?

I What were you noticing, when you were reviewing with the resident, that helped inform the score that you provided?

R Well 'cause we were reviewing, it wasn't in – it wasn't in front of the patient, so it was really considering what they were telling me in their presentation, so pertinent positives, pertinent negatives that were included, and when there was some uncertainty in what she thought was going, or what the – I should say, what the resident thought was going on, the resident actually already had done a few steps to help alleviate some of that confusion, and also plan ahead. So, what are steps they had take to just in case they're wrong, there are safety steps ready. Those are things that I noticed when she was presenting - or the resident was presenting.

I Okay, and did you have any overriding concerns at that point?

R Nope, nope.

I Okay, and did the resident's performance earlier on have any impact on your score?

R I haven't worked with this resident a lot, so I don't think so.

I So it was based kind of solely on that review with the resident?

R Yes.

I Did you have anything else to add in terms of the information you used to give the score you did?

R	Anything in particular?

I	Yeah, I was curious if you had anything else in terms of the information you gathered, or if I can move on to the next question?

R	You can move on to the next question.

I	Okay, what were your specific goals when you filled out this form?

R	Specific goal? Um, it was, ahh, I guess to provide an useful evaluation back to the resident, but we already kinda discussed it, in person. That was just formalizing the feedback I already gave her in person.

I	Okay, so just giving the resident some feedback , in person, and what were you trying to communicate with her?

R	What she was doing well, and then things that she can work on to further to improve either her presentation or management.

I	And how did this form help you with these goals?

R	Well it blended quite nicely, the way the form, especially at the end, about what they're doing well, so they know the things that they should keep doing without having to waste more time trying to improve it, and then just a few pointers – highlight to what they need to work on and what they're doing well very nicely so that residents don't have to kind of guess what we're thinking.

I	So it kinda provides you a way of communicating that. How did this form interfere with these goals?

R	Interfere? I was a little bit – I didn't realize this was a new form, so the milestones kinda popped up, and I guess I don't read things carefully, so they said if you select it, you'd skip question 7, which is the milestones, but I kinda filled it out as well, so yeah, so that was new.

I	Okay, it was just kind of new so you weren't sure how to quite fill it out – is that right?

R	Yeah, well , yeah – well, not – it's quite – it's *[something inaudible]*, right, in progress, they need work on or it's achieved. I just didn't expect those – the set of questions for the milestones.

I	And I don't have your form in front of me, but I think she scored in that top category, so you didn't have to fill it out – is that right?

07:32

R	Yeah, technically, I didn't have to, but I already clicked the first one, but yeah, 'cause I kinda reviewed – what I selected and then realized I didn't have to fill it out.

I	Okay, here let me just pull it up to make sure we are on the same page. Okay, so how did the presence of the milestones impact your assessment?

R	Impact my assessment? Not a ton, since I thought she was doing well already.

I	Yeah, okay.

R	But I guess, if I did have any concerns, it does help break down where they can work at, right.

I	Okay, and how do you feel filling out this form, overall, helped or hindered teaching or patient care?

R	I guess you're supposed to do this in person. I find that a lot of residents actually just, you know, like, we kinda do verbal feedback, but then they email me the thing afterwards. So, in terms of hindrance, not much. It's just my time afterwards to fill it out and I was a little bit surprised with the milestones, but that's where they can fill in the case info – often they do that. I don't know if I'm supposed to do that little case part.

I	Okay, so just so I'm hearing you correctly, it's just kind of a timing thing for you to fill it out after they send it to you?

R	Um hmm, yeah.

I	And that maybe'd be more beneficial if you were to observe them, but they – it sounds like residents often kinda fill it in and then send it to you afterwards?

R	I think residents feel bad that they want you to stay late – later after your end of your shift, right, just to fill out the evaluation, or it's busy in emerg – one or the other – and I'm leaving. So, then just often go 'hey, do you mind if I email you an evaluation to fill out later about this case that we just went over'.

I	Okay, I think that's all the questions I have for you. Is there anything you wanted to add about the form or your experience that you weren't able to?

R	No, I think – yeah, no, nothing else to add.

I	Well, thank you so much for calling me and participating in the study, and you have to send me your consent form sometime within the next week, and we'll send you a gift card when the study is finished.

CD1.11.P
07:32

END 07:32

CD1.25.P
11:11

I First this is interview CD1.25.P, and what I'll have you do, [name], is just to think back on the form and kind of relive the moment as if you were filling it out, so just kind of go back to that time.

R Sure.

I And if you could please describe if this encounter was more about direct observation of skills or about reviewing approach to diagnosis and management.

R Okay, so this one was someone I saw with the resident, overnight and evening. I would say it's more of a review of his approach to the problem. I didn't directly observe him taking the history, so it was based on what he told me and based on my assessment of what was happening, so I would say it's indirect, and it's mainly with the diagnosis and kind of figuring out the etiology as well of some of the management issues around it. So I thought, when he pulled up the EPA, it was pretty easy to set it all up. He had it all done pretty quickly, and then I think the only thing that I would have thought was a bit different was section. It looked a bit different, especially with the milestones part. I don't think I've seen that before in the other EPA's I've completed, so I thought that was a bit different compared to the other ones.

I Okay, yeah, that's the new thing, I think, that they've added in, which is what were hoping to get feedback on – the new milestones, yeah

R Um hmm.

I So, what were you reviewing with the resident, then, that led to your decision to choose your score?

R I think it was maybe, overall, his global assessment, in terms of how safe he was, how well he was able to come up with a differential, and then how – if he was able to come up with, kind of, a management plan with some contingencies kinda built in. So, I think, based on that, was what I kinda evaluated him on, from my perspective.

I Hmm, did you use any other information in making your decision to give this score?

R Hmmm, in what way? Can you give me an example or -- ?

I Like, was it specifically just this interaction with him and what he presented to you? Or maybe did his performance earlier on have an impact on your score? Things like that.

R No, no, this was, I'd say, based on just what he reviewed with me.

I Okay, and what were your specific goals when you filled out this form?

R I'm not sure. He was the one who brought it up, so I would say, yeah, I thought it would be more on, like – when I was filling out – like, the management of that condition, but then when I read the milestones, it kind of became a big vague, which I thought was not as helpful as it could have been.

I It wasn't as specific as you wanted it to be?

R Yeah, like, in terms of – it kind of broke down things that I probably wouldn't have separated from, for example, the differential, I think there's, like, two parts about prioritizing a differential and then selecting the test for the differential. Like, I would have probably, you know, evaluated him on his ability to gather information, his ability to interpret the information, and then ability to act on it. Whereas, I thought they kinda even broke it down a bit too much where it was too specific – like, it was too small of a thing to evaluate.

I Okay, it was too specific?

R Like, too narrow in scope is a better word.

I I see, yeah, so I noticed you gave constructive feedback about keeping broad differentials.

R Um hmm.

I And then in the milestones, you didn't score him in the section that says 'generate and prioritize differential diagnosis'. Can you ---

R Oh, did I not score that at all?

I I don't –

R I think it was just, like, an 'achieved' versus 'in progress', or was there more options?

I No, it was just 'achieved' or 'in progress', but I guess, like, yeah, I'm curious 'cause you didn't score him 'in progress', but then gave him the feedback that he should be keeping broad differentials. I was hoping you could speak a little bit more to that.

R Like, he did come up with a differential but, you know, I gave him a few more things, so I didn't think he was deficient, per se, which, you know, when I read 'in progress' or 'areas to focus on', like, it was kind of binary thing. It's either, yes, like, achieved or it's not achieved, so I thought it wasn't as bad as, like,

completely negative. Maybe if they had another option, I would have put it, but it just seemed like it was either this, like, 'in progress/areas to focus on' or it's 'achieved', and so I felt like it was kind of a – almost a binary decision.

I I see, okay, and I know you spoke to this a little bit already, but how well do you think the milestones help you communicate with the resident how they're progressing in the program and what they need to do to progress?

R It's hard. Like, I don't think it helps me, in general. Like, I think I view it much broader than these select things. I think I view it – like, the stages I look at is are they able to report information, are they able to interpret, are they able to manage it? So I think this was, you know, broken down into a lot more kinda nitty details, and so I think that makes it – I mean, it's good if they actually had some parts, but there's a lot of these things that I thought just didn't apply – like, 'referral to another healthcare provider' – that section I thought didn't apply at all to this case.

I So you kind of referred to the – even though it wasn't applicable, you kind of just marked it as –

R 'achieved', yeah.

I 'Achieved'

R Like, I didn't have a choice to say 'did not assess', you know?

I Yeah, I see what you're saying, and what were you trying to communicate to the resident when you were kind of going over this form with him?

R I think it's mainly what I typed in, which is to tell them not to kinda to anchor down on one diagnosis, per se, so that's why I said to keep things broad, so that's probably my biggest feedback to him 'cause I think he probably got the right one, but it's important just to do that thinking exercise, so, you know, I wasn't sure if he had done that. Like, he seemed pretty certain about what he thought this was, so I thought – that's why I put that as an area for improvement, but I thought he was actually pretty appropriate, overall.

I How did this form help you to communicate that to him?

R Hmm, I think it was just the free text. I thought the milestones wasn't – like, it wasn't flexible enough to help communicate. Like, I thought it was just, you know, 'yes, no, yes, no', right. Like, it just didn't have enough flexibility to communicate what I wanted to say, if I just click on the yes's and the no's.

I Okay, so it was that little comment box that allowed you to kind of elaborate/

R Yeah, I think so, yeah; whereas, you know, the ‘in progress’ and even the ‘achieved’, you know, I don’t even know what it means. Like, what does ‘in progress’ really mean? Like, normally, I think the part above is a bit easier. Part six, like, ‘does not have approach’, ‘can assess’, ‘can assess and manage’. Like, that one – you know, a few more options would be helpful. Whereas the bottom I thought, I’m not sure. Like, ‘in progress’ is that a completely did not do it.

I Right, it’s a little subjective what that actually means?

R Yeah.

I Okay, how would you say this form might have interfered with some of your goals to communicate with the resident, if it did?

R I don’t think it interfered at all. I think there was just parts that weren’t as applicable?

I Okay, and then I just have one more question. How do you feel filling out this form, overall, helped or hindered teaching of patient care?

R Hmm, I don’t think it hindered patient care, and I thought, teaching-wise, I could see when it would be helpful. Like, if they had specific things that I thought they could improve on, and if the form had that explicitly written, I think that would be a very good way to say, ‘hey, this is actually something you need to know’, but I think, in this case, it didn’t work out that way. Like, sometimes, for example – like, I’ll give you another example. Like, I have learners who sometimes will use medical terminology instead of layperson terms, and so sometimes when I’m filling out these other EPA’s, there’s a part where it says that – whether they’re able to communicate clearly or not, use simples terms and stuff, so sometimes I find that helpful because you can point to them, like, what they can improve on. Whereas, I thought this one, they kinda highlighted some domains, but then I thought – I really didn’t like the ‘in progress/achieve’ thing. Like, I couldn’t make much sense of it. I think maybe if there was a bit more options – a few more options – or maybe if there’s a free text about it, or ‘do you have any comments about this area?’, I think that might be a bit more helpful.

I I see, okay, well I think that’s all I have for you. Is there anything you wanted to add that you weren’t able to? I guess, you know, these rating scales, I’ve seen it a lot of different ways, so far, and I think that’s – the challenging part for me is it all seems like it’s all very – like, the end – the last box is always just, like, ‘meets objective’. Whereas, sometimes I thought maybe there’s a bit more nuance to it, like I think they did actually a very good job, but I can’t actually reflect that in there, and it seems like most of the choices are in the negative, and so – like, you know, ‘lacking in this’ or ‘deficient in this’, ‘needs help in this’. Whereas, I think, you know, there’s a bit more range, actually, even above competence that we’re kinda missing out, right.

I	Hmm, missing a bit of a strength paced(?) approach?

R	Yeah, like, ‘this person did very well on this part’, you know, so it’s always just kinda caps it as ‘sufficient’.

I	Okay, well thank you so much for your time today and calling me back.

R	Yeah, no problem.

I	Yeah, we appreciate your feedback, and hopefully we can use that to revise these and make these a little bit more useful.

END 11:11

I Since I don't have your written consent form, I'll just ask, do you consent to participate in this study?

R I do.

I So what I'll have you do is just look at the form in front of you, and then visualize and relive them moment that you were filling it out, as if it was on video.

R Okay.

I Okay, so can you describe if this encounter was more about direct observation of skills or about reviewing approach to diagnosis and management?

R I recall and remember it being more about review of diagnosis and management

I Okay, and what were you noticing when you were observing or reviewing with the resident that led to your score?

R I was recalling that, mostly in terms of the presentation of the case, it was the presentation of both the history – physical – as well as his overall symptoms, the various investigations, and then coming to the conclusion of the diagnosis, and him presenting his management, so it was very much a classical type of situation where it was outpatient setting, based around a computer screen, and discussing the case.

I Okay, was there any other information you used in making the decision to give the score you did?

R Not that I can think of or recall offhand. I will say that it was pretty much moreso what he was presenting to me, as well as the various investigations and the actual direct reports of those imaging evaluations and blood tests that we were reviewing at the same time.

I And would you say the resident's performance, earlier, did it have any impact on your score at all?

R I would say yes. I mean, that case that we were going through would have been halfway through the month long rotation, so I had already had some experience with them prior to doing evaluation.

I I see, and what were your specific goals when you filled out this form?

R My goals were more just to see whether I could reflect upon his performance as accurately as possible.

I Okay, can you say a little bit more about that?

R Yeah, I mean, I was trying to make sure that, both, the evaluation was reflective of what I thought was going on, but as well as to key in some feedback directly to the trainee, just for the fact that one of the things I find with this is that it prompts immediate feedback after an encounter has occurred.

I Um hmm, and how did this form help you with these goals?

R I think it worked quite well. I mean, certainly, trying to provide feedback in clinic can sometimes get washed under, just for the fact that it can be quite busy, so it was nice to be able to bring that up right away on the spot.

I And how did this form interfere with these goals?

R I think it aligned quite well. I mean, the – one of the nice things that I really like is the fact, as long as the trainee's doing well and you say that – that you think that they're doing quite well, then hits achieve on the other milestones, and so just quickens the process.

I Um hmm, so how do you think the presence of these new milestones impacted your assessment?

R Not greatly. I think it moreso just adds language or an actual, I guess, written language what, I think, we think of in our heads, on an abstract way, with respect to what we're actually evaluating for skills, so I think it's just a nice concrete presentation, really.

I Um hmm, alright, I just have one more question for you. How do you feel filling out this form, overall, helped or hindered teaching or patient care?

R I think it helped with respect to, again, doing immediate feedback with the trainee. As I was saying, that perhaps some of the things that they have with the milestones is a little bit more concrete, as opposed to abstract thoughts as to what we have, or at least ideas of what we think a trainee should be accomplishing during the clinical encounters with patients. Yeah, like I said, I do like the quickness of the reporting – the, you know, clicking one essentially clicks the rest. My only fear is that, in order to make this really quick manner is that, if a physician or, basically, a preceptor is trying to make things fast, they may just decide to click it, even though they may or may not feel that way, necessarily. I mean, that's not the case with my evaluation of this trainee, this time around, but I can see that as a potential pitfall for others.

I I see, okay, well, is there anything else, maybe, that you were unable to add that you wanted to add to this? 'cause that's all the questions I have for you.

R	No, no, I think that’s pretty much about it. I mean, it’s always nice to have bit of free form at the end, particularly to have specific feedbacks so what not for the trainee, so no, I thought this actually is a nice initiative.

I	Alright, well thank you so much for your time. I know you’re really busy, so I appreciate you chatting with me.

R	Awesome, thank you.

END 05:28

CD1.82.P
13:24

I Yeah, there's really no risks or rewards, but we are offering gift cards to either Starbucks or Tim Horton's when it's over. What is your preference?

R I guess Starbucks is the preference.

I What I'll have you do is , just thinking back to the form or looking at the form, can you just relive the moment you were filling it out? Okay, so can you describe if this encounter was more about direct observation of skills or about reviewing approach to diagnosis and management?

R So, we did a little bit of both because this resident was the evening senior. She had admitted a number of patients, and then I met with [name] during the evening, sort of midway towards the end of her shift, maybe, and then we started reviewing the patients together, and so she had already seen and assessed and made plans for the patient, so then we reviewed the patients at the bedside together, and we went over her findings, and we also discussed, in the consult room, about the plan, and was it appropriate or was it not, and was her approach good or not. So, we assessed a little bit of both of those things that you said, yeah.

I What were you noticing when you were observing and reviewing with her that led to your score?

R So, I could see that she understood the issue before the patient came to hospital, and did appropriate physical exam and reached appropriate or correct diagnosis, and then her plan was very reasonable and was really what I would have done, and so in hearing her assessment and plan, I thought it was very close to, if not the same, to what I would have done and what I would have said about the patient, which is why I gave it the evaluation that I did.

I Okay, and what information did you use in making the decision to give this score?

R Well, with the – I used the information that she provided. So, she gave me her impression and her physical exam findings, and then I went over the story as well and examined the patient myself, and reached the same conclusion as she did, so yeah, I mean, I dunno if you'd call it truth checking or….I'm not sure what the proper term for it is, but basically I checked what she told me in her I found her physical exam assessment to be correct, her review of imaging to be correct , and her impression of the case to be correct, with the information we had at hand, and her plan was what I would have done, so that's why I gave her the scores that I did, right. I think I gave her full marks, if I'm not mistaken.

I Yeah, it looks like you did. Did her performance earlier on have anything impact on your score?

R	So I had worked with her once before, and then, when I previously worked with her, I also found her to be very good and her assessments to be correct, so, you know, that just put me a little bit more at ease when I was hearing her story. Like, I didn't feel like I had to question everything that she said, you know. So, knowing her or having had an experience with her in the past allowed me to take what she said more at face value than I would have with another resident, I guess.

I	What were your specific goals when you filled out this form?

R	Well, to get the form done, and then to give her a fair evaluation.

I	What were you trying to communicate with her?

R	I was trying to reinforce the good job that she did.

I	Okay, and how did this form help you with these goals?

R	So, it allowed me to say whether she was doing what she was supposed to do or if she needed help with the case at hand. I also found the comment section useful, you know, if there was something to add on top of what was in the check boxes. Yeah, so I guess those things helped me communicate, yeah.

I	How did this form interfere with these goals?

R	It didn't really interfere too much. I found it was very – it has a lot of check boxes. I'm not sure that they needed as many check boxes as it had. So I think, not that it took that much longer to click two or three extra boxes, but I thought that maybe there was too many check boxes, before we would have only about two or three, I think, and it served the same purpose.

I	Okay, so the other form was a little bit simpler but got at the same thing?

R	Yeah, yeah, I think so.

I	And how do you think the presence of the new milestones impacted your assessment?

R	Honestly, I was surprised when I saw it 'cause it was a form I had seen before, but then I just assumed that something changed and I wasn't told about, so I just went with it, but, I dunno, I just – it's a pretty straightforward form to fill, and yeah, I mean, not too many issues with filling it. The one thing I would say about the form is I filled it after the fact, so I had [name] email the form and I did it at home on my own laptop. I think , because it is a little bit of a longer form, usually what we do on these evening shifts would be to give the senior feedback on the spot,

and then fill out the evaluation form that night, you know, before we let them go home. But when the forms are getting longer, it puts an extra challenge to fill out this form at midnight, before letting the senior go home, so it makes it so that you – it's not as easy to do in the middle of the night, and I prefer to do it at home, and then feeling that you run the risk of forgetting about it at home.

I Okay, and I just have one more question for you. How do you feel filling out this form, overall, helped or hindered teaching or patient care?

R I don't think it impacted it very much. As I said, I filled it up to the form, and I wasn't aware that the form had changed when I did teaching around the case with [name]. Looking back on it, and I don't know if this is gonna be the form to use going forward. It makes you wanna question the resident a bit more on some aspects of their presentation so that you can fill out the form a bit better. So, if you look at the milestones thing, it's got one section saying 'generate and prioritize official diagnoses'. You know, we don't always question the residents on that, and so, now that I see that this is on the form, this is gonna be the standard. You know, we would circle back and we would quick the residents a bit more about what else could be going on, then to – like, the next one is 'select and interpret appropriate investigations based on a differential diagnosis'. Again, we wouldn't necessary grill the senior residents on this, on a regular shift, but if we have to tick a box whether they achieved or if they have to work on it, we would potentially have to go back, or you would extend the evaluation of the case on a given date. So, I think, yeah, if this is the form, going forward, knowing that there are 1,2,3,4,5,6,7,8 – 8 milestones that we have to evaluate for the residents, then we would spend much more time reviewing each case, in particular, and maybe quizzing the residents a bit more on various aspects of the case, to be able to tick these boxes properly, and the impact on this would be maybe it's gonna improve teaching, or maybe you'll find an area of weakness that otherwise you wouldn't have, but it might also lead to spending more time reviewing cases when there's – or taking longer to review any given case when your under time pressure with a lot of patients to see.

I Okay, well that's all the questions I have for you. Is there anything else you wanted to add?

R I think that the other thing that I would say is, so this form seems to be with regard to one particular case or one particular consult, and a senior resident can, on one of their shifts, see in between 4 and 8, maybe more cases, and so, I guess there needs to be some clarification whether we do a form per case, or do we do a form that evaluates their overall performance during the shift? 'cause I think, if we do a form per case, then it would get kinda ridiculously long to try and evaluate a resident after the end of the shift versus, if there's a form that encompasses the entire shift, I think that's maybe a bit more time efficient, both for the resident who submits form to us and for us to fill them out, and one thing that we don't want to do is run into the trouble of form fatigue.

I Okay, well thank you so much for your feedback and participating in this study. Yeah, we value what you have to say, and I know you're really busy, so thanks for taking the time out to chat with me today.

R Yeah, no problem, I hope this helps.

I Take care, and just send me your consent form whenever you have a chance, within the next week or so.

R Sure

END 13:24

CD1.99.R
13:05

I And you can just send me your signed consent form within the next week.

R Yeah, sure.

I Yeah, you can just email that to me, but since I don't have that from you, I'll just ask you your verbal consent. Do you consent to participate in this study?

R Absolutely.

I Perfect, okay, so this is interview (incorrectly identified), and what I'll have you do is, if you can look at the form or remember it, I'll have you just relive the moment you were filling out the form, alright?

R Yeah, sure, let me just pull up the –

I Sure, and I am recording you through the speaker phone, so if you can speak as loudly and clearly as possible that would be much appreciated for the transcriber. Okay, what were you noticing when you were observing or reviewing with the resident that led to your score?

R Like, just going through the form, or just with his approach? I guess this is more about the form, right?

I Oh, 'cause you didn't – this wasn't a direct – okay, so I should start with the question –

R Yeah, it was a direct observation. It was from a direct observation, but I didn't have the form right at the time, so I filled it out with the residents a couple of days later.

I Oh, I see, okay.

R Yeah.

I So, I guess, what were you noticing when you were observing or reviewing the resident that led to your score later on?

R Okay, I mean, as an individual, he was, you know, very well organized, and he had a very good, you know, practical approach the case at hand, which actually was kind of a complex case, had a lot of issues to deal with, not only just the myeloma presentation, but other sort of general internal medicine stuff that was clouding everything that he had to wade through, so he did a really good job of that, and then, yeah, was basically able to take the whole case through to completion, to the level that I would have expected somebody at his level. So, you know, I don't expect him to know all the ins and outs of management, but he

highlighted the key problem areas, and had a general approach for those, and then did a – made a reasonable effort at even sorta delving into the management of myeloma from a chemotherapy standpoint, which, for the most part, was correct. So, on the whole, I think he did well. Looking at the form and sort of, you know, knowing how did with the case, and then thinking about how to apply it to the form, I think the form actually seems to be a reasonable way to – and probably better than the prior forms we had to fill out, so a better way of assessing where somebody's at, and I think that, for him, he was fairly easy, and to categorize if he can assess, diagnose and manage the case with some minor change to the approach, just, you know, to re-direct for things that are a little bit more specific and probably outside his scope of expertise. So, on the whole, he did well, and I like the way that the form is set out, so it gives an opportunity to kinda highlight where there are a few gaps – not inappropriate gaps, though. I guess that would be maybe one minor quibble with it is it's not tot – like, sometimes I think it may come across to residents that they're missing something, you know, or a big ticket item is in progress or area on which to focus. I think it's kinda – so, you know, for a type A personality, people that generally find them in these professions, it's hard for somebody to say 'you're not quite doing it' (laughs), if that makes sense, but that is the reality, so, I guess, on the flipside of that is people have to get used to that kind of constructive criticism, yeah.

I Yeah, okay, so what information did you use in making the decision to give the score you did?

R Well, by and large, it was his review of the case with me, and then our discussion together with the patient afterwards. So, he saw the patient first; we reviewed the case, and then we went back in together and sorta summarized everything to make sure the patient and the family were on the same page. We had a bit of a discussion about him afterwards, as well, and then I also had the clinic letter to go over, as well.

I And did the resident's performance earlier on have any impact on your score?

R Like, with other cases, you mean?

I Yeah, was it kind of specific to this case and what you saw in what he presented to you, or did anything else prior to that have an influence on your score?

R I think it's kinda hard to not have his – the time in clinic - his other time in clinic influence it 'cause you sort of always – you know, from where you see all that, the resident, is at based on how they do with other cases, so yeah, I think it would be somewhat foolish of us to not allow it to enter in because – you still want to have some way of talking about their global approach to these sensitive cases.

I Okay, and what were your specific goals when you filled out this form?

R Like, my personal goals? (laughs)

I Yeah, personal, professional.

R To get it done on time. (laughs) To get it done on time; to actually complete it with our busy clinic and stuff is hard, but actually, this form is nice and quick and short. It was a bit of a surprise that way, and it felt like well maybe there should be more here, but as I said before, I think it actually does capture what we need to capture. I wanted to make sure that it was – the feedback highlighted that I thought that he was doing probably above average for somebody with his level of training, in our – you know, busy the clinic was kinda complex patients, complex treatment paradigms, so I wanted to make sure that that came across, but I also didn't want to put something down that I didn't feel was present. So, you know, he clearly doesn't know how to manage myeloma; he wouldn't be able to run a clinic by himself, and that wouldn't be expected, anyway, but you still want to have that sorta highlighted. On the whole, it was – you know, so those would be my main goals. I think, as more generally speaking, I would want to have an opportunity, I guess, to make sure that, if I had resident that I thought was struggling, that there was ample place, I guess, to describe that, and I'd probably have to go back and look at the form a bit more closely to see if that was the case, but it's kinda difficult to really have judgement on that aspect of things , unless you have a resident that comes through that actually is struggling, and figuring out who to make sure that you highlight those areas.

I Um hmm, was there anything you were trying to communicate to the resident, in this case?

R Like, just with the case, in general, or in regards to the – how I gave feedback?

I In regards to how you filled out the form, was there anything else you were trying communicate to him, beyond what you alluded to?

R No, I don't think so. I think I was – I mean, I was able to actually to discuss with the resident afterwards too.

I Okay, and how did this form help you with your goals?

R Like, goals in the clinic? Well, I think, number one, it's short, so it's easy to complete and to get done.

I Okay, let me rewind. I guess I'm curious how this form helps you with your goals in communicating with the resident or the goals that you spoke to just previously to that question.

R Yeah, I think it gives a good – gives a good framework to have that discussion happen, yeah, and everybody's sort of aware of the salient points that we would be expecting of them, and the categories that are there are ones that are very relevant to just the standard day-to-day practice and management of getting through a clinic.

I Okay, and I know you kind of spoke to this a little bit earlier, but I'm wondering how this form might have interfered with these goals.

R Interfered with them?

I Yeah, you were speaking a little bit about, you know, some of the downside of the form, but I was curious for, in terms of your goals, how might this form interfere with it?

R I think the – it being brief, sometimes some of the detail that you might want to highlight, if you felt like there was a concern, is lost. Like, it's an easy form to fill out if you have really no concerns with the resident, and, you know, a couple of quick boxes to type out and some more descriptive stuff, but I don't know how it would play out with a resident that was having problems with, say, content or management or something like that. There the onus – there's a little bit more onus on the teacher and the evaluator to provide that feedback in the pros boxes and cons boxes.

I Okay, how well do the milestones help you communicate how the resident is progressing in the program, and what they need to do to progress?

R I think that they are fairly accurate. I mean, they're very general things, though, right, so it does get away a little bit from what I would expect a resident to know about the diseases that I'm interested in, but maybe that's a bit of a misguided thought process for many of us, and that not everybody that's gonna come through out clinic is gonna be a myeloma expert in their career, so we just want them to be competent physicians, to be able to take a history and, you know, basically determine that very, very basic question of sick and not sick, and provide a bit of a management plan, and also to recognize their limitations, and when to – and be able to verbalize that, and ask for help when they feel that they need to. So, I think, on the whole, it does capture that.

I Alright, and I just have one last question for you. How do you feel filling out this form, overall, helped or hindered teaching or patient care?

R I mean, again, I get back to the idea, like, it is a nice and quick and easy for to fill out, so it's easy to do in the midst of clinic or shortly after clinic, so you can get it done in a timely fashion to give feedback that's relevant to the resident in that moment, which is, I think, a real strength. It is broad enough, but still captures

sorta the basic things that we want to be able to capture for residents of that level, so I think that's good. The drawbacks, I guess, is that maybe it is a bit too vague and bit too broad, but that, again, just puts more emphasis on the evaluator to make sure that they communicate across to residents what they feel that they would want them to know in the clinic, and I think, if you can kinda meld this with the overall expectations for a specific rotation, which residents have separately, and their own goals that you know that are more disease site specific or system specific, I think that they would probably play off each other fairly well.

I Okay, well that's all the questions I have for you. Is there anything else you wanted to add?

R No, I don't think so.

I Okay, well thank you so much for chatting with me. I know we really appreciate your participation in the study, and hopefully it will benefit the form and the residents down the road.

END 13:05

I If you have the form in front of you, that would be helpful; if you can remember it, that would be helpful too, but just think back or look to it, and then relive the moment the preceptor was filling in the form. Does that make sense?

R Um hmm.

I Can you describe if this encounter was more about direct observation of your skill or about reviewing your approach to diagnosis and management?

R I think it was more of the latter.

I Okay, and what do you think your preceptor was noticing when they observed or reviewed you that led to their score?

R I think they were noticing some of the positives in terms of my medical expertise knowledge in the case, as well as some of the negatives with respect to that same medical expert role, in terms of knowing what to do in this case, whether it's assessment, and I think, in this case, it was also management as well.

I Okay, and what do you think your preceptor's overriding concerns were at that point?

R I think the concerns were that there was a few aspects of the treatment plan, including some of the specific medications, that I was not as familiar with, and so I think the reason that they chose the rating was to say that there is some change in approach was because they did need to make some changes to my final management plan in order for it to be the best for the patient.

I What information do you think the preceptor used in making the decision to give you this score?

R I'm not entirely sure what you mean by that.

I Like, did they observe you? Did they base it on kinda of previous experiences? Were they watching your presentation? Like, what information do you think they took into account to give you the score you did?

R Hmm, well because this was the first time that I had worked with this particular preceptor in this area, I think the assessment was based solely on my discussion with the preceptor after doing the case, so I reviewed the case with them after seeing the patient, and then they based that assessment just on that because they didn't know me from before.

I Okay, and do you think they only used this interaction – the presentation that you gave – or do you think they used other observations from that day?

R I think it's possible that other observations may have played into the – into this assessment, but only very slightly because I only reviewed two consults with this particular preceptor, and so it would have only been the other case that could have contributed, but the other case was a bit different, so I think this was – my assessment was fairly focussed on just the case at hand.

I Okay, and what was your specific goal when getting this form filled out?

R Well, I think there were a couple of specific goals. One of them – the most obvious – being that it's a requirement of our program, moving forward, is to complete this forms, but a second goal is that I find that doing these EPA forms does force preceptors to give at least some feedback; whereas, if there is no form, then sometimes the feedback gets delayed further or just gets missed in the busyness of the clinical schedule.

I And how did this form help you with your and your preceptors goals?

R So, for me, it helped me to get a sense of how I'm doing, overall, in this area, and it did help me to focus on if I needed to read about something or focus my self-directed learning on a particular area. It did highlight some of the areas in which I could focus on, and then, I think, for the preceptor, I think it allowed them – in this case, I'm not really sure what their goals were, so I'd have to sort of guess, but let's say that this preceptor was actually filling out a One45 evaluation or, like, an end of rotation evaluation, I think it would help them to be able to reflect more deeply on my performance, rather than them just kind of using an overall gestalt of my experience with them to make that assessment later on.

I Okay, so just to clarify, you feel like, even though you don't know 100% sure, you feel like your preceptor's goal was to help you to further reflect on that experience – is that right?

R I think my preceptor's goal in doing this was – well, I'm actually – I don't really know what their goal is, but one of their goals could have been to – like, in terms of goals for themselves would be, so that if they had to fill out an evaluation of me later on, because they've done this more detailed reflection, then it would help them to provide a better assessment of me at the end, as opposed to them have not gone through this process of doing this more detailed feedback.

I Okay, yeah, and we know you don't know what they're really thinking, and we're just curious about what your thoughts are, so that works. How do you think the preceptor might feel about how filling out this form, overall, either helped or hindered teaching or patient care?

R So, I think that, in terms of the teaching, I think that the preceptors probably thought that this was more helpful because it did provide some specific feedback

for me – for me to learn from – and then, in terms of patient care, because this was filled out after the clinic, I guess it didn't directly negatively affect patient care, and potentially, because of me becoming more competent after getting this assessment, it could potentially improve patient care for patients that I look after in the future.

I Okay, and then I just have a question that I accidentally skipped over here. How do you think this form might have interfered with your and your preceptor's goals?

R I think – I'm not sure about this specific form, but I think, for similar forms, I think one way that it could interfere with goals is that, because does it take some time to fill out, and it is ideally best filled out right after reviewing the case, even if that's in the middle of a clinic or in the middle of a work day, sometimes that could take up some extra time and slow down the efficiency of the clinic or the workday for the preceptor and for the resident, so I think that's one way that it could interfere.

I Okay, well I think that's all the questions I have for you, [name]. Is there anything else you wanted to add?

R No, I don't think so.

I Okay, one thing I forgot to mention is that we are offering ten dollars Starbucks or Tims to the participants after the survey - or after the study is done, which we'll send to you, so I'm curious what your preference is – Starbucks or Tims?

R Tims would be preferable.

I Okay, awesome, I will mark you down for that, and yeah, I have your consent form, and that's all I really need from you, so thanks so much for participating in the study. We appreciate your feedback.

R Okay, no worries, thank you.

END 09:46

I Any questions for me?

R Not right now, no.

I Okay, sounds good. So since I don't have your written consent form, I'll just get you to email that to me whenever you have a chance.

R Yeah.

I I'm just going to ask, do you consent to participate in this study?

R Yup.

I Okay, sounds good. So what I'll have you do is just to visualize and relive the moment that you were filling out the form, as though it's on video.

R So, what do you mean?

I Just try to remember it, like, as if you're replaying it in your mind like it's on video. The moment you were filling out the form, just to go back to that time.

R Oh, sure.

I Yeah, I'll just have you – yeah, that's like my instruction for you.

R (laughs) Alright.

I So I was gonna ask you if you can describe if this encounter was more about a direct observation of skills or about reviewing approach to diagnosis and management?

R It was – you know, this was about approach to diagnosis and management rather than any skill related question. You know, the case summary and presentation surrounding an individual with inflammatory bowel disease with some localized symptoms of shortness of breath and hematochezia. From the emergency department, we had – it was afterwards, in order to evaluate the individual encounter and review. I guess, from my standpoint, it's helpful having a timely opportunity to document and review this, so the resident himself brought it up that, you know, he's instructed that he needed to have a certain amount of focussed evaluations for review and assessment, so it was nice that there was a very short lag period in between when we had done the initial assessment and when we tend to have formalized feedback. So, as far as the form itself goes, you know, it was separated into broad categories of types of presentation, and then a very small descriptor and describe the case, which is useful in order to kinda anchor specifically to the case we were talking about, and kinda very

generic sub-headings such as location of the encounter, the supervisor rank, and whether or not there was direct observation. So, I find that the form itself was quite easy to go through and didn't take much time, and then in regards to the evaluation section certainly is a lot shorter than the previous evaluation forms that I've had to do, and so, yeah, at least the – my thought process was this would be a much easier process to do on an immediate basis, rather than the prior forms that we've had that certainly can take five or ten minutes in order to fill the entire form out.

I Okay, so when you were filling this out, what were you kind of noticing or observing, which led to the score that you provided?

R Sorry, like, so the score that I provided, at least in the case, would be – was that the individual can diagnose and manage the case with no significant changes to the initial management plan, and so in that regard, we had – it was a very – it was nice to have a summary option for the case. So, the individual I was reviewing touched on, fairly completely, the differential diagnosis and what his management considerations were and the subsequent plan, and so, at least within, you know, number six or, say, four kinda very broad options into whether or not there is a complete approach, whether or not there is some initial changes required, or whether they have a basic approach or none at all, and so kinda from my thought process standpoint, it was nice having this kinda global heading here, especially when there is very little concerns that were coming up in the evaluation of the individual. And so, at least in this specific case, I did not use the subsequent milestones grouping, which was question number seven, for areas to focus improvement on. So, I did take a look at them, and certainly, in broad strokes, I think it allows, from the evaluator's standpoint at least, a better way to try to focus on areas for improvement, rather than the large [long] generic forms that essentially spew out like a 1 to 5 number for the individual.

I Okay, so you didn't use the milestones, but how do you think the presence of the new milestones impacted your assessment?

R Well, it kinda focuses the area of – where you are doing your assessment and subsequent feedback for the individual, so it highlights the important areas of your – of the clinical assessment, so recognizing whether or not there's any urgent or emergent problems there, completion of the entire assessment, differential diagnosis plan, and then developing the subsequent management plans. I find that it's much more – I find it useful in order to frame a reference for evaluating somebody, and then potentially even sending them feedback 'cause oftentimes you get kinda lost in how best to evaluate someone or review someone, so I found these kinda subheadings are useful in order to say, alright, well we can look at these milestones, and are they making them, or are there any other concerns that come up in these kind of subheadings?

I Right, okay, and I was just curious, did the resident's performance earlier on have any impact on your score?

R What do you mea – oh, like, for prior interactions with them?

I Yeah, like, did you kind of just base your decision off of the presentation that they gave you, or did any previous interactions kind of have a role in your decision?

R Well, you know, when you're going evaluations on a specific individual, problem we try to, ideally, take into account how they've managed things in that individual interaction, at least that's what I try to do on a – at least when you're evaluating, - not as an entire individual, but on a specific interaction. Now, sometimes there is – like, if you're looking at it from the milestone standpoints, they don't always – they aren't always relevant to the single case that you're talking about, so this would be, like, the last subheading of 'identifying patients requiring hand over to other physicians or healthcare professionals' may not be relevant in all cases. Certainly, at the end of the day, on an emergency rotation or when you're admitting patients it might, and that's where sometimes might like, come in, but you have to think about, alright, and I think it more comes up if you're using this from a global approach for evaluation somebody, but on the individual standpoints, I try to take a more focussed evaluation to their – kind of a current presentation that we're discussing.

I I see, okay, and what were your specific goals when you filled out this form

R Pardon?

I I was curious what your specific goals were when you filled out this form?

R So, well goals for my standpoint, so, you know, the request is to provide, ideally, timely feedback to say, you know, are things going well? Are there any areas that are coming up for concern, or kinda what are specific areas for improvement that I should give? So, in this specific case, there was a complete, focussed examination, relative to the clinical problem. You know, complete approach to the clinical assessment and differential diagnosis, so, you know, my goal is essentially to say, yes, things are going well – or identify, you know, yes, things are going well, you know, things are going mostly good but here's areas for improvement, or here's where you need to really improve on things, and ideally what I would like to do or I see some good use for this is trying to document on a, ideally, as close to real time basis, you know, objective areas for improvement and feedback in a timely fashion for people so you are not running into issues kinda three, four weeks, months down the road and, you know, sort of then getting concerns or issues, where I could have improved previously, so that's kinda where I see this quite useful, yeah.

I Um hmm, how did this form interfere with these goals?

R I didn’t see any.

I Okay.

R There is a free subheading box – or a free text box to put, you know, where you have focussed concerns and in free form standpoint, so I think that’s always a useful area to have, where you’re not just giving tick boxes or checks; you’re able to give kind of a focussed review in a short few sentences of mini-paragraph.

I Okay, and I just have one last question for you. How do you feel filling out this form, overall, helped or hindered teaching or patient care?

R I’m a little bit neutral on it. Now, certainly, I think it more as a evaluation for the resident, in order to get feedback for their overall performance. Now, certainly, that can then come out in the way of teaching around areas of where you’re having deficits that have been identified, or you can give teachable moments in regards to, you know, ‘these are the areas that I think that you’re doing quite well on, and you should continue to do this and learn to build on your areas that have shown that good performance in the past.’, but, specifically, these forms aren’t always – so that’s where, you know, I could see you have teachable moments that come out of, kind of, focussed review of the presentation in the initial case. That certainly could be incorporate into, kind of, overall, how you approach teaching the residents, but that’s not, at least, how I’ve been viewing or been using it as.

I Um hmm, well I think that’s all the questions I have for you. Is there anything you wanted to add to this?

R No, not at this time.

I Okay, well I really appreciate you getting back to me. I know you’re really busy, so yeah, we appreciate your participation in the research.

R No worries. Yeah, well hopefully it’s helpful.

I Okay, thank you so much.

END 12:38

FD1.37.P
04:42

I You've read over the consent form and everything? You've consented?

R I'm cool with it, yeah.

I Okay, do you care about the gift card?

R Yeah, I didn't notice the gift card.

I What I'm gonna have you do is just to think back on the form and kind of relive it like you were there, and if you can just describe if this encounter was more about direct observation of skills or about reviewing approach to diagnosis and management?

R Hmmm, let me just look at what I did with him. Okay, ask me again what your question is.

I I was wondering if it was more about direct observation of skills or about reviewing approach to diagnosis and management.

R The latter 'cause I was on call with him, yeah.

I Okay, and what were you noticing when you were observing or reviewing with him that led to your score?

R Hmm, I mean, it's a bit of a gestalt, isn't it, sort of. Obviously, what I put down – that he's giving the pertinent positives and negatives, that he's confident about what he's finding, it's detailed, yeah.

I And what information did you use in making this decision to give this score?

R So, the information he provided to me. Sorry, what do you mean?

I I was wondering if you can elaborate on that. Like, were you just going based on what he said, and -- ?

R I'm looking up the case in Net Care and seeing whether it's not it's accurate, but yeah, it's what he said. It's just over the phone, right?

I Did the residents performance earlier on have any impact on your score?

R I hadn't worked with him before this, so –

I Okay, so no?

R No.

I Okay, and what were your specific goals when you filled out the form?

R Hmm, to complete the form ‘cause I had too (laughs), and because, I guess, to try and give him some positive feedback that he’s doing great.

I Okay, so you’re trying to communicate that positive feedback to him?

R Yeah.

I Okay, and how did this form help you with these goals?

R It has, yeah. It was actually pretty easy to fill out.

I Oh, I was curious HOW it helped you.

R Well, achieve those goals. It allowed me to give a positive feedback.

I Okay, and how did this form interfere with these goals?

R It didn’t. I mean, I gave it to him verbally as well, so –

I Okay.

R But I suppose it’s necessary for the record, right?

I Yeah, and how do you think the presence of the new milestones impacted your assessment?

R Umm…….I don’t think it did.

I Okay, and then just last question – how do you feel filling out this form, overall, helped or hindered teaching or patient care?

R You know, it might have had more of an impact in a situation where the resident was not as good, but I think, for the good ones, it’s not that difficult to evaluate them, not matter what the form is, right?

I Do it didn’t really apply here?

R Not in this particular case, but I can see that it might be more helpful if – I mean, it seems more simple to fill out and more relevant than the previous form, so that might have been an issue if he wasn’t as good.

I Okay, did you have anything else to add?

R Nope. This is the form that's in use. You guys are just doing a study to look at how people to like it, or to adapt it or -- ?

I Yeah, we're hoping just to get people's thoughts on how they filled it out, and then hoping to adapt it to improve it.

R Yeah.

I Yeah, so we're just listening to people and how - what their experience was, so yeah, if you can just get me your consent form whenever you have time. I know you're really busy, so just in the next couple of weeks would be great, but other than that, that's all I have for you. Thank you for touching base with me.

R No problem, and I get my $10 coffee card, I guess?

I Yeah, do you want Starbucks or Tim Hortons?

R I'll just give it to my grad student. I'll get – she likes – I think she likes Starbucks yeah.

I Okay, thank you.

END 04:42

FD1.37.R
14:08

I Do you have the form in front of you or do you just remember it?

R I have it in front of me.

I Okay, so if you can just look at that form, I'll just ask you to relive the moment the preceptor was filling out the form.

R Well, so actually, this form filled out by [name], the FD1 common acute one, was actually filled out – I did the save for later option, and that was sent to her later, and so, basically, I guess, the steps to getting it filled out were that I, first of all, had just done a night of call with her, and I asked her if she would fill one out for me. I actually had, like, numerous cases that night, and she probably could have filled out four or five, but, you know, I didn't want to bother her too much, so I got one done, and I asked for an unstable patient because I really had an unstable patient, and I managed them sort of without her assistance for the bulk of the night. I called her at 5 am just to involve her and sort of tie things up for the morning, and so then, in the morning, after GI hand over at 7:30, so at around 8 I asked her if she would fill one out, and she said, 'absolutely', and she said, 'could you just send it to me', and I did, and actually it did take one reminder email to get her to fill it out, so I waited a week and then I – which is sort of, like, what I've been doing for the people who don't fill them out, which is most people. When you do the save for later option, they seem to sort of need a nudge, so with that reminder email, she filled it out the next day, and no issues or concerns, and, at the time when I had asked her about filling out the EPA form, she said she absolutely no concerns with my work through the night, not specifically about the – oh, sorry, sorry, she did a common acute medical – I apologize. [name], I'm mistaking the two. [name] did unstable acute; she did the hypotension case, I think, or fever. It was a cholangitis patient, I think. They're technically both, so it sort of fit fever and hypotension and also shortness of breath, I guess, but still, you can only choose one. So, yeah, so she said she had absolutely no concerns, and that was that.

I Okay, so I'll just ask you some questions, and maybe even though you didn't observe her filling it out, maybe just answer just how you think 'cause a lot of this is just us wondering about what your perceptions of them filling it out was, so just answer it to the best of your ability, I guess.

R Sorry, can you repeat that?

I Yeah, I said, even though you didn't observe her filling it out, just answer the questions, like, based on your perceptions, to the best of your ability.

R On the form?

I Yeah

R Okay, yeah, so, I mean, the first one's a really straightforward, like –

I Oh, so I'll – it's okay, I'll ask you questions, and you can answer each one.

R Sure, yeah, absolutely.

I Yeah, sometimes people think they just have to, like, freely associate, but it's okay.

R Yeah, sure.

I So, was this more of a direct observation of your skills or more about reviewing your approach to diagnosis and management?

R Reviewing the approach, for sure, yeah.

I Okay, and what do you think your preceptor was noticing when they observed you or reviewed you, which led to your score?

R Well, so basically there was a gap between when we talked, so it was, like, basically 11 pm to 5 am, and between that gap I was seeing probably about five patients, and this one in particular that required quite a bit of work, which is why I sort of added that patient into the EPA, and so I guess, she – she never really – she never really saw what I was doing, but we basically just talked about everything in sort of like – basically, not quite a case summary, but a summary of the events from 11 to 5, basically, was what we had talked about.

I Okay, so she didn't actually watch you, but she was scoring you based ---

R No, and that's the case with most of these, realistically, because how often are you working, especially on call, obviously, but other than being on call, just like day-to-day, the staff isn't on the ward that often, unless you're doing team rounds, and even when you're on team rounds, you're not actually individually doing something; you're doing it as a team, so how can you ask them to do an EPA based on your approach? Like, maybe occasionally you'll have a staff – like [name], for example – who asks you questions, and not necessarily grill you, but sort of try to understand your thought process of approach and diagnosis and management, but really, on call, they're not asking your those questions ; you just do it.

I Okay, so, in your presentation, that was what she was basing her score off of.

R Yeah, and it was basically, like, I sort of walked her in, and, of course, like, a case sort of summary of, like, 'this is guy X that you've heard about at 11; this is what's gone on since then; this is what I'm thinking; this is what I've done, and

this is what I plan to do or have ordered and am thinking about.' So sort of just that, like, catching her up on the events of the night.

I Okay, so what information do you think she used in making the decision to give you this score?

R So, the big things were, first of all, that she – like, I think that she appreciated that I thought through things broadly – like, I sort of did a differential based approach on what was going on. I thought about all the pieces of information that were available to me, being labs, physical findings, and also trends of things overnight, and, like, I had the benefit of actually having a set of trends in terms of the – like, these were the patient's LFT's at this point in time; these were the patient's LFT's at this point in time, which was, like, extremely important to the case, and then she appreciated that I had further steps to the management; it wasn't close ended. I had sort of made sure that the case would be continued appropriately, onwards, from the point of me leaving the hospital, and for people to be able to actually, like, follow up on the case. I didn't just sort of clean my hands of it. I made sure that there was good follow up, and I – you know, in proper handover, you like to give people sort of the plan, right, I didn't just say 'this is what was done'. I said, 'this is what was done and this is the plan, and I think that that's what she appreciated most, and I think it was the fact that, sort of, with, like, any patient case, it's just like sort of quality. You're thinking about what's going on and what needs to be done eventually, so, like, those future management steps, and also, like, a broad differential approach is really appreciated by any of my staff. That's sort of what I've learned. That's what we're taught, but, you know, it's harder said than done – harder – yeah, yeah, you know what I mean. (laughs)

I Yeah, I do. Do you think that the preceptor only used this interaction, or do you think they used other observations from that day?

R No, I think she used the whole day. Realistically, I think that that individual EPA was sort of an assessment of my competence on call with her, and so I think that the approach that I used with that patient was sort of used with, like, the – I think something like seven consults with her. It was a really busy night, and I think she really appreciated that I took independent steps. She was, like, very happy that an R1 was sort of functioning at the level where I didn't need to call her several times during the night. I mean, just from a pure sleep standpoint, I'm sure she appreciated that, but also, you know, I did call her at 5 to catch her up on things, and to sort of re-involve her, to get back – like, to basically – you know, I took independence without sort of overstepping my comfort zone, and I think that that was what she actually appreciated most, yeah, and it was definitely the picture of the whole day. I really think that that's what that EPA funnelled down to, realistically, and I think that's, technically, a lot of EPA's as well, and I think, like, that's sort of what I'm seeing and feeling, at least. It's more a sense of your overall quality of work, rather than that individual EPA, yeah.

I Okay, and what was your specific goal when getting this form filled out?

R Well, my goal? So, I know that, like, the right goal is to, technically, like, have certain skills checked off, right. Like, I want – in this case it was either, like, an acute presentation of fever or hypotension (laughs). Like, this guy really filled out all of them. He was definitely altered all in all sense, but she – like, my – I guess my goal was around, first of all, just managing a patient, individually, and being able to sort of assess them, and go from, like, initial assessment to future management plan in the hospital, and that's sort of , like, my goal with every patient, at this point in time, especially on call because I'm trying to get ready to be, you know, be on emerg senior, which is what we're doing next year, so that's, like, sort of my goal is to be individually competent, and to not have concerns with, you know, my sort of comfort zone, and at least, you know, not just keeping someone alive but, you know, sort of working on how to figure out what's going on, what might happen, and to have, like, that future management plan, and while, like, this EPA is really just the acute presentation, really, I mean, like, other than just getting all the EPA's checked off – and I needed this one – it's, like, having good skills and approaches with all of these presentations, but while these cover a broad differential of presentations – like altered LOC, chest pain, fever, hypotension, shortness of breath and others – like, that is every – like, I guess that encompasses everything because there's the others as well, but really, like, I do want to have a good approach with all of those. The EPA didn't really, like, change the way that I approached the patient at all, and getting it filled out was mostly just like a tick box for me, but, like, I approach ever night on call wanting to actually be competent at all of those. Like, I want to be ready for any patient because that's sort of what we're supposed to do in internal medicine.

I Gotcha, and what do you think your preceptor's goal was when filling out this form?

R Truly, I think it was just her sort of satisfying my need to get it filled out. I don't think – like, I know that it's supposed to be an opportunity for me to get feedback, and I sort of got that from her at 5 am and then at 8 am the next day, but the feedback at 8 am, in person, was mostly just, like, sort of like a pat on the back. At 5 am we sort of reviewed everything that had happened over the night, and she gave me a few, like, clinical pearls, suggestions as to management plans, and of course, like, a few things changed, on a few of the patients, that she wanted done. Like, very few things. Just a few labs, check up on person X, make sure not blank – those sort of things – but really, other than just those small sort of clinical tidbits, it was mostly a pat on the back at 8 am instead of, you know, like a thorough feedback sit down session because just, like, at 8 am, after GI handover, the hepatology team has to go do hepatology and see the patients, so she's not gonna sit down with me for fifteen minutes to fill out that form, yeah.

I How do you think this form helped you with your and your preceptor's goals?

R I think it was just sort of a formal representation of what she otherwise would have told me, and, you know, while I appreciated that, I think that our discussions were also really satisfying for me, and I didn't really need the paper or electronic sort of formal feedback. I was satisfied with the way that we interacted during and after the call shifts, yeah.

I Okay, how did this form maybe interfere with some of those goals?

R It didn't interfere with any. It's just it's sort of extra stuff that I have to get filled out, and so, while it is a formal representation of my ability to approach an acute presentation of fever or hypotension, really what I think – like, it just – to me it just sort of added the step to sort of show that I had had someone at least review my approach and management of fever and hypotension in the acute setting, but I really don't – it didn't so much interfere as it was just this sort of, like, extra hurdle, yeah.

I Okay, and how do you think your preceptor might feel about how filling out this form, overall, helped or hindered teaching or patient care?

R I truly don't think she – like, I think, for her, it was just another opportunity to give me sort of confirmed feedback and, you know, I appreciated her kind comment in the form, but I don't think it really changes her interaction with me or her feedback giving process with me, especially in the context of the save for later function where you don't have that sit down session, so, like, when you do have this sit down session and they actually fill out the form, they usually – they give y you a comment. You know, they say, like, 'good job; I probably would have done x; you're doing a really good job around your cases.' Something very broad and general, but at least you have that formal talking session. A lot of the time, you don't have that formal talking session. When you do the save for later, they sort of just say, like, 'good job on call', and then you get the form, and it sometimes says something insightful; sometimes it doesn't. Just, like, people are busy.

I Okay, well I think I've captured a lot of content here with you. Was there anything else you wanted add, [name], that you weren't able to?

R No, no, I think I've got it in.

I Okay, cool, well thank you so much for taking the time to call me back and do the interview. We definitely appreciate your research participation.

R Yeah, let me know if you need any other talking tidbits. Always happy to talk. (laughs)

I Oh yeah, cool. Thank you very much, take care.

R Thank you, have a great day.

I Yeah, you too, bye.

END 14:08

I What I'll have you do is - well, first of all, I don't have your consent form, so I'm just gonna ask you for verbal consent. Do you consent to participate in this study?

R Yes.

I Perfect. So, this is interview FD1.44.P, and what I'll have you do is, just thinking back on the form, relive the moment as if you were filling it out, so kinda just revisit that time, and if you can describe if this encounter was more about direct observation of skills or about reviewing approach to diagnosis and management?

R I think this was more about diagnosis and management.

I Okay, and what led up to your decision to choose the score you did?

R What led up to me choosing the score that I chose?

I Yeah, if you can talk about how you made that decision, what information you used to make that decision?

R Well so, I dunno whether you've reviewed what I actually wrote.

I Um hmm.

R The scenario that the resident posed for me to review was actually somewhat of a – I mean, it's a bit of an unusual scenario that wouldn't really be a general internal medicine resident kind of a scenario, and so, in truth, before the resident went to see the patient, we had already had a long lead in conversation about this very unique nephrology problem, and so, you know, in many ways, I kinda think he didn't choose a very good example of admission or of a patient interaction that he had with me because he performed quite well in what he had to do, but the night before – and this was during a call night, and we knew this patient was going to be coming, was going to be presenting to the emerg, and so that – but the night before, before I left the hospital, I had had a very detailed conversation with him about, you know, what we would be expecting, what we're looking for and, you know, I gave him a fair bit of kinda forewarning, teaching, education around this unique problem. If he had been a nephrology fellow, then I wouldn't have done quite so much and would have let him think about it more, but there is now way that he would have had any real understanding about what he was going to be seeing a few hours later, if I hadn't prefaced the interaction the way that I did. So, in many ways, what he – all he had to do is really kind of, you know, regurgitate a bit of the conversation that he and I had had. So, when I was filling it out, even though he did a very good job at what he did, I actually had to think back at, you know, well how am I gonna evaluate him? 'Cause he did a good job, but he did a good job because it was framed in such a specific way

that, like, anyone would have done a pretty good job because it’s just not a general internal medicine problem, and so, as I was filling out that particular scenario, as opposed to the other one that he sent me, or over the weekend I did two others for a different resident, they were all kinda more bread and butter stuff, and I had a lot easier time filling out those ones rather than this one, where I really had to think, yeah, he did a good job, but I don’t just want to say he did everything awesome because he wouldn’t have known about that had we not have had such a thorough conversation ahead of time. Does that make sense?

I Yeah, so it sounds like you kind of had to preface some of it, so –

R Yeah, and so I hemmed and hawed a fair bit about how to fill out that EPA ‘cause, in reality, I kinda felt like it wasn’t really an EPA I should have been filling out.

I Hmm, I see.

R You see what I mean?

I Yeah.

R It wasn’t demonstrating all that he knew; it was really just a pretty unique – like, I mean, this was such an esoteric kind of nephrology thing that my guess is there would be a whole lot of senior nephrology fellows who, without prefacing, might not have even understood the significance of what they were seeing.

I Um hmm, okay.

R And so, if you asked me to go back into that moment when I filled that out, you know ,the others ones that I filled out previously, you know, it was like ‘bang, bang, bang’ easy. This one, like, I was thinking well what the heck am I gonna write? And so that’s why you’ll see that, you know, what I wrote was sort of more kind of vagueries, instead of something specifically for him to work on. There isn’t anything specific for him to work on in that scenario ‘cause he’ll never see this again unless he becomes a nephrologist, and even then, he may never see it as a nephrologist, and so, you know, I felt a little bit like – I felt hesitant around filling it out.

I I see.

R Does that make sense?

I That does. What were your specific goals when you filled out this form? I know it was a little bit tricky, but what were you trying to accomplish, or what were you hoping to communicate to the resident?

R Well, first off, that I thought he did a nice job with the scenario that he had at hand, and also to reassure him, which is the second that I made in the open comment dialogue box, saying that, you know, like, don't worry, you'll never see this again so it's not a big deal, depending on whether you felt confident or not confident, it's such a unique problem, you'll never ever encounter this again, but I also – I wanted to give him some positive feedback because this particular resident is a – my interactions with him were very, very positive, and, in reality, you know, I sort of took the philosophy around what I observed all day from him, and brought that to bear, to the EPA on the case that he asked me to review, and even though the case wasn't all that relevant to a general internal medicine EPA, you know, I wanted to be reflective, in some ways – and maybe this is wrong; I don't know – but I wanted to be reflective of the overall general positivity that I had with all the other things that we did throughout the day. The trouble is he didn't send me an EPA for all the multiple other things we did; he sent me one on this particular esoteric case. Otherwise, if it had been a different EPA, I would have, you know, remained much more specific to the particular action and interaction that I was being asked to evaluate, but this one didn't fall into that category.

I Okay, so you took kind of a more broader assessment, taking in multiple interactions.

R Yeah, yeah.

I Okay, and how do you feel like this form helped you with your goals to communicate with the resident? Does that make sense?

R You know, not really.

I Like, when you were speaking to how you were giving the resident feedback, and hoping to kinda tell him he did a good job but maybe this is something he's not gonna really see, how did this form, specifically, allow you to communicate that, if it did?

R Yeah, it forced me to put something down on paper, so I had to think about it and be explicit, which otherwise it's easy to gloss over that, but a form makes you do that.

I Right, okay, and then just on the reverse of that, how might have this form interfered with some of your goals, if it did?

R I mean, it didn't particularly, and again, I think this particular EPA just wasn't really appropriate for GIM. I mean, I have to say, if I think about the form, in general, one of the things that I find frustrating, in having filled out a few of things, is being forced into writing something about what the resident could have done better. You know, I mean, I find that a bit aggravating. Sometimes a resident just

does a really great job on the skill that they're supposed to have, and there really isn't anything to do better, and that's perfectly reasonable, you know, and I suppose somebody could turn around and say, well, there's always something to improve. You know, maybe they could have smiled more, maybe they could have held the patient's hand better, maybe they could have done X, Y or Z, but that's just – that's just minutiae. Sometimes they also just do a really bang up job, and there isn't any criticism that you can give them, and I think that oughta be reflected in a form – when they're doing a great job and you really can't add anything.

I Yeah, for sure. Okay, so in this form, you said he was doing a good job, but it looks like you still kind of scored him in the milestones, right? How well do you think the milestones themselves help you communicate how the resident is progressing in the program and what they need to do to progress?

R Well, hang on, now I have to go back to look at the form because I'm – give me a minute. If you want, you can turn the recorder off for a second 'cause I actually have to find this thing.

I It's okay.

R Hang on.

I Let me know if you have a hard time looking for it 'cause I can just re-forward it to you.

R No, no, it's okay, I've got it here. So, under the milestones, yeah, so, I mean, it kinda depends because some of these milestones just aren't relevant to the case.

I Hmm, so some of them you chose because they didn't apply?

R Yeah, so it would be lovely if there was an option that said, well, this just isn't really applicable.

I Right. So, the ones that you scored 'in progress', am I hearing you right in that those ones weren't applicable?

R No, not necessarily. I think I probably deferred to the 'no problem' and 'achieved otherwise'.

I Okay, in kind of thinking about how well the milestones helped you communicate to the resident, aside from some of them not being applicable, how well do you think it allowed you to kind of communicate some of those things that he needed to work on?

R I dunno, I mean, I think they're pretty self-explanatory.

I Okay, and then I just have another question for you. How do you feel filling out this form, overall, helped or hindered teaching or patient care?

R The form itself was fine. I mean, what I found was that it would have been nice to have been given the opportunity to do this right away, like right after we reviewed the case or saw – but it wasn't until a day or two later, I received the email asking to have this filled out, and the resident asked me ahead of time, verbally, and then it took another day for the email to show up, and then, admittedly, the way in which he had crafted the scenario – let me just see if this is the right one – yeah, there was the way in which he had written it earlier, and because I edited it. When he said, 'describe the case', he had described a case, but I think he had put together two separate cases into one, and it was – you know, it was a night on call, so it's easy to understand that there may have been some confusion in recollection as to what specifically the case was, but he had written some stuff in the 'describe the case' that was irrelevant to the actual case, and so had to go back and I had to text him, and said, you know, 'look, can you be explicit? Who were you actually referring to?', and so then I went up and I edited the 'describe the case' section in that section number two.

I Right.

R So, it would have just been nicer if, you know, after the interaction or the next morning, he had handed me his phone and I could have just, you know, kind of done it right into his phone, right away.

I Yeah, okay.

R So, this very specific EPA interaction with this resident actually took probably five times longer than any other EPA that I've ever done, and it just would have been so much easier to have just done it right then and there in the moment.

I Okay, was there anything else you wanted to add?

R No.

I Okay, perfect. Thank you for your time, [name]. I know you're really busy, so we appreciate you participating and sharing your thoughts about filling out the form.

R Sure, no worries.

I Yeah, so whenever, in the next couple of weeks, you have some time, just feel free to send me the consent for.

R You actually need me to send it?

I Yeah, I do need you to send it, but there's no rush, so within the next couple of weeks is fine.

R Well, so here, we're gonna do it right now.

I Okay.

R 'Cause otherwise it's not gonna get done.

I Sure.

R And then is your address on there somewhere?

I Yes, I should be cc'd on that email that you're looking at.

R Okay, I'll send you physical – I'll just – yeah, okay.

I Perfect, thank you so much.

END 15:15

FD1.49.R
13:16

I	So since I don’t have your consent to participate in this study, I’ll just ask do you verbally consent to participate in this study?

R	Um hmm.

I	Okay, perfect.

R	Okay, sure.

I	And we are giving away gift cards for $10 for Starbucks or Tim Hortons, after the study is over. Which one do you prefer?

R	Anyone…it doesn’t matter. You said Starbucks or what?

I	Tim Hortons.

R	Oh, Tim, yeah.

I	Okay, perfect, I’ll mark you down for that. So, this is interview FD1.49.R, and were you able to review the EPA for that [name] sent?

R	Sorry, again?

I	You can kind of remember the EPA form or pull it up, that I’m gonna ask you about?

R	Yeah.

I	Okay perfect, so, yeah, so just, I’m gonna record you through the phone, so if you can speak as clearly as possible that would be great.

R	Sure.

I	So what I’ll have you do, is just, thinking back on the form, can you just relive the moment the preceptor was filling out the form – just kind of go back to that time – and describe if this encounter was more about direct observation of your skills or about reviewing your approach to diagnosis and management?

R	No, it’s reviewing.

I	It was reviewing?

R	Yes.

I Okay, what do you think you preceptor was noticing when they observed you, or when they reviewed the form – or reviewed you – to lead up to your score?

R I actually told the management all over.

I Sorry, can you repeat that?

R Like, they were discussing with me ‘what’s your differential?’, and then I was, like, going through my approach, step-by-step, and then, he was asking me, ‘okay, what do you want to do next? What do you think about next?’, okay, and then ‘what is your plan?’, so that’s kind of that part. He was just giving me a question, and then I’m expressing my thoughts and my approach and then my plan.

I Okay, so he was asking you –

R Yeah, yeah, and then, eventually, he said, ‘okay, I agree’, and then he added just one blood work– one more blood work to my plan.

I Okay, so he listened to you kind of talk through the presentation and what you would do or what you were doing?

R Yeah.

I And the he said he agreed?

R Yeah.

I Okay, what do you think that your preceptor’s overriding concerns were at that point?

R No, he didn’t mention any direct concerns.

I Okay, he didn’t mention anything?

R Yeah, yeah, he had that more blood work; just to broaden up my differential diagnosis.

I I see.

R And to execute more disease, but no actions. Like, he didn’t direct to me for any concern.

I Okay, what – I know you spoke to this – but what information do you think that they used to help make the decision?

R Sorry, again, can you repeat it?

I Um hmm, what information do you think your preceptor used in making the decision to give you your score?

R From my point of view – I didn't discuss that directly with him – but from what I noticed, it's my approach to the case and my differential.

I Your approach to the case and your differential.

R And differential, and eventually my plan.

I And your plan, okay.

R Yeah, so I have my reasoning to exclude some differentials and to involve some differential, and based on that, that was my – like, I wrote my plan accordingly.

I I see.

R And he seems, like, very agreeable about it.

I Right. Do you think that they used only this interaction with you, or do you think they used other observations from that day or from before?

R I think it's more work.

I You think it's more what, sorry?

R Like, you said verbal interaction or observational?

I Oh no, I was wondering, do you think that they kind of marked you based on this one interaction with you? Or do you think they – maybe if you'd known them before or if they've reviewed with you before?

R No, no, no, he was just covering for that night, and he didn't – yeah, he – like, I was on call with him.

I Um hmm, so it was just this specific interaction?

R Yeah, yes.

I I see, okay, and what was your specific goal when getting this form filled out?

R What was my specific goal?

I Goal, yeah, what were you hoping to accomplish when you get this – when you got this form filled out?

R For example, no, I didn't get that question, sorry.

I Oh, I'm just wondering, so you got this form filled out, right.

R Yes.

I What do you want from it? What were you hoping for? What were you trying to accomplish? Does that make sense?

R Yeah, basically, it's trying to accomplish to make sure that if I have similar case in the future, I would be able to handle it independently.

I I see, hmm, so you were hoping – how would this form help you in the future, then?

R Yes, yeah, yeah.

I Um hmm, how – I'm asking how you think that form would help you in the future, then, handle new cases independently?

R Yeah, getting the feedback – like, getting the feedback, or discussing it with more experienced staff, that's for sure. It will give you, okay, you should have thought about that, you should have did that, and that will broaden my mind to do it independently in the future.

I Okay, so getting feedback. How did you – what do you think your preceptor's goal was when filling out this form for you?

R I didn't, first of all, my preceptor for now, as I am R1, I think it was teaching or making sure that I learned something new, making sure that I have a good differential and good approach to the case.

I Okay, trying to kind of help you 'cause you're an R1.

R Yeah.

I I see, okay.

R Yeah, that's what I felt because I think, if I am R3, I think the expectation of my preceptor will be definitely different.

I Um hmm.

R Yeah.

I How do you think this form helped you with your and your preceptor's goals?

R I think it – like, same area as I mentioned before. Like, it's – it kind of outlined what should I approach by the end of doing this case, and also outline, for my preceptor, what they should be looking at or what they should be observing.

I Um hmm, it provides and outline.

R So I think it's just making my targets and my preceptor's targets are in the same line because, if they are expecting something and if I am expecting or aiming at something else, I think that would be difficult to communicate with the preceptor, but having this form, I think it's – yeah, it's unifying my target and my preceptor's expectations.

I Unifying your target and your preceptor's expectations, alright. Okay, and how do you think this form interfered with your and your preceptor's goals, if it did?

R Not actually interfered. I don't think it's interfering, no. It's just the time – like, the time it has to be filled, and the paperwork around it. Let's say that just the logistics around it.

I Just the timing and the logistics.

R Like, I have to tell him – yeah, I have to tell him that I have a form, then he has to fill in the form, and then we can fill it. I fill my part and he fills his part, so that kind of – yeah, like lots of these forms maybe sometimes overwhelming for the residents.

I Okay.

R Yeah.

I And how do you think the preceptor might feel about how filling out this form, overall, helped or hindered teaching or patient care?

R In this case, I felt that my preceptor was welcoming to do it, but it's not the case with other preceptors because he was very welcome to do that ,and he told me that he is ready to fill out the form, so he offered. That's why I think he was very happy to do it.

I Hmm, what is the other cases where that might be how it works out?

R You know, nobody will refuse, but somebody will say, 'okay, email the form for me', and they will never fill it, and another one say, 'okay, let's do it another time'

because of a busy service, or because I am working with another preceptor later, so it would be difficult to get a time to fill that form out with him, so I think it is too difficult, may make it, like, unapproachable, or I have lots of EPAs that was done, and because of that and because I have done some rotations in other hospitals, I have still the feedback with the preceptor, and they were – they told me, 'okay, email the form to us', and it was never filled. Like, I lost almost 15 EPA's because of that.

I Okay, wow. So, do you think that speaks to what you said before about logistics and time?

R Yes.

I Okay, yeah, well that's all the questions I have for you. Is there anything else you wanted to add?

R No, that's – yeah.

I Okay, well thank you so much for –

R But the only thing I would add, regarding the milestones, from my point of view, I think it will be very, very overwhelming, and in utopian world that would perfect, but in our actual world – because I'm among other residents and I can take their opinion – and myself, and my opinion, that will be very overwhelming if they have to do all –

I It would be overwhelming to fill out?

R Yeah, the milestones. Like, I think a simple form would be much better.

I Um hmm, this one's a little bit complicated you're saying?

R Yeah, yeah.

I Because it would be time consuming for the preceptor – is that what you mean?

R Yes, yes, and I think, in terms of practicality, milestones are designed to be followed in the future by the same preceptor, and they have to observe that same resident, right, which is not the case in resident training. Every week we have a different preceptor.

I You're saying, for the milestones, it should be the same preceptor filling it out for the resident, so they can follow through, with time, and kind of compare?

R Yes, that would be very difficult.

I Okay, ‘cause right now you have it filled out by different people?

R Sorry?

I Right now you have it filled out by different preceptors, and you’re not finding tit as helpful?

R I don’t have that situation yet, but I think my expectation is that it will be difficult.

I I see, okay. Alright, thank you for your feedback, and thanks for getting back to me.

R Okay, you’re welcome.

I We’ll chat with you later, and feel free to – just some time within the next week or so, send me the signed consent form, but not rush.

R Okay.

END 13:16

FD1.71.P
12:51

I Since I don’t actually have your form yet, I just want to make sure, do you consent to participate in this research?

R Yes.

I Okay, so what I’ll have you do is just think back to the form as if you’re reliving the moment, and describe if this encounter was more about direct observation of your skills – or about the resident’s skills – or more about reviewing approach to diagnosis and management?

R I think it was both, actually. They admitted a patient, and then they admitted the patient, and then they discussed, you know, what to do, but also he assessed the patient, so –

I Okay, and what were you noticing when you were observing or reviewing with the resident that led to your score?

R So, I mean, I think this form now is a little more complicated than it used to be, and I’m not sure this is an advantage. The more details you’re introducing into the form, I think, the more difficult it’s gonna be to – it’s not about honesty, obviously – it’s just not gonna really cover exactly what’s happening. So, my – I mean, obviously, you can’t just say something is in progress or achieved, even if you are not 100% sure about it, which is not really easy to do this without really – like, you know, stray from what actually is happening there. That’s my concern with the form. So, it used to be more simple, and that was, I think, better because, if you had any kind of comments, you could still make the comments, and that was about exactly what’s happening, and this is not necessarily so. I mean, if you want to give me examples, I can give you examples, I guess.

I Okay, yeah, sure.

R So, I mean, generally, differential diagnosis along with appropriate diagnostic strategy. So, differential diagnosis is not a question when a patient is known to be admitted with an ST elevation myocardial infarction, you know, differential diagnosis is not gonna be the question here. It doesn’t even – you know, it doesn’t come up, or ‘develop and implement initial management plans’. This patient was admitted from the cath lab, so, you know, then was thrombolysed at reperfusion or not – I can’t remember – but anyways was going to the cath lab, had a stent, and then we are admitting this patient with a CCU, so again, what is the management plan? Well, I mean, basically, everything that had to be done with a STEMI has already been done. This is routine for the CCU that we are basically admitting them after the initial management plan is already done. Okay, so and this is basically – if – you know, you get my jist, right? This is basically the problem. If we are too precise about this – I mean, I can say it’s in progress. It has nothing to do with the resident.

I	It has nothing to do with the resident, yeah.

R	Yeah, because it's nothing to do with that particular case because most of these cases – you know, this form is probably good for an undifferentiated admission to general medicine where we have no idea what is going on, and we have to kind of find out – you know, diagnose the patient, and then go through a differential diagnosis and treat accordingly, but in CCU, most of cardiology, this is done way before the admission.

I	So, what would you do differently? Or what would your idea of -- ?

R	Well, I mean, you could still continue. If my opinion about this is reality– like not the not the norm, or the average case that gets reviewed is still – you know, these are applicable to that average case, then fine, don't change anything, but this was easier to handle, and we did have to, you know, half heartedly answer all these specific questions that did not apply. It was easier when it was just, you know, basically, a comment on how he is doing. I mean, he was doing his job; he was doing what was expected of him – assess the patient. We still need to look at is this patient in heart failure, had any kind of complications? He needs to know what he's doing, so I can quiz him on a lot of things, but not really what you're asking here.

I	Um hmm, you don't feel like the form's capturing what you should be assessing?

R	Yeah, the form doesn't really capture what is happening in CCU, and that is basically – for the most part, that is where I'm seeing patients, currently – in patients – and that's where I'm testing resident's knowledge.

I	Okay, so how did you come to rate the resident the way you did then? How did you make a decision?

R	I basically just said it's achieved. I mean, it had nothing to do with him. Other times, obviously, it's him in the emergency room, so I know he can make those decisions, but this is not beside – I mean, I have to say something because, if I don't say either, then it's just not gonna allow me to continue.

I	Okay.

R	Right? So, this is the – I saw this as a little bit of a problem. Again, I'm comparing this to the old version where you just had to say, are you actually relatively confident that this resident knows what he's doing? And that – and if I have any concerns, I can raise them, and they also, as you know, want me to comment on at least one area for improvement. I mean, that's also a little bit of – I mean, basically, the residents know when they perform something well, and then those are the cases where they want you to sign off on, right, for obvious

reasons. So, usually there's not much to comment on that could be improved upon. Those are the other cases that they don't ask me to comment on because they know what they were not performing that well. (laughs).

I Right, you want them to be showing the stuff they need feedback on, but that's not what they're showing you

R Yes, but if I'm commenting on something, if I'm criticizing something, then likely they're not gonna end up wanting me to do this in writing, right, so –

I Yeah, so you said you were observing the feedback in the emergency room. Was there any other information, specifically, you were using to come to the conclusion you did?

R No.

I No?

R No, I don't think so.

I Yeah, were you just observing him or talking with him?

R Yes, he was reporting, basically, what he found, and then we examined the patient together and it was fine; it was accurate.

I I see, and did the resident's performance earlier on have any impact on your score?

R Yes, yes, yes, yes, it did because, yes, you are right. I mean, I did kinda take into consideration that I have been working with this guy for two weeks, so I kinda know him, yes, and obviously, when I'm giving him feedback, it's based on two weeks experience, so I am – you know, if I'm criticizing anything, it's not about this particular case, but it's about overall things he can work on.

I Okay, and what were your specific goals when you filled out this form?

R What do you mean 'specific goals'?

I What were you hoping to intend or accomplish?

R They have to have this knowledge. It's not what I am hoping to achieve by this; it's that they must have my signature on this, and so, you know, with the comments and anything, eventually they have to do the EPA's, right, so that's –

I I'm just guessing – I'm trying to think, what were you trying to communicate to him?

R Well, in this particular case, again, he was doing well. He was doing fine, except that it was the form didn't apply, word by word, but he was fine, so there was not – but again, this is by design. They have to ask you to fill this. It's not that I'm filling such a form about everything, okay, so they know when they are fine. These are usually cases without any complexity or, you know, particular problems, so they want to be successful; they want the cases signed, and they won't want me to comment on lots of things that I can criticize.

I Okay, and do you feel like this form helped you?

R It doesn't, I don't think, at all, no, that's the point, but it's not even about me, obviously. I'm happy I have an opportunity to explain my concerns about this you asked, but, for the residents, this is about having ten signatures that they can perform this and can perform that, so that's basically it. It's a must for them.

I Okay, so you feel like there's other ways this form can interfere with some of the goals? I know you've mentioned some of them, but ---

R No, I don't think this is a detriment. I'm just thinking that this is not really helping anyone. This is, obviously, documenting that something happened, but I think what is better for the residents, from a development perspective, I think it's better if I can give them some meaningful feedback based on two weeks performance, and I do that, obviously, but you need more than just one case, obviously. You need to have two weeks, and well that's a different discussion we could have. I'm happy to talk about that too, but that is, I think, better and more meaningful the resident because I will remember a few things that I want to comment on, and whether they feel this is just or not, that's another matter, but that is fine. Overall, it's difficult to find one way of evaluation that's meaningful to both parties, and obviously the program also has to see that something is going on with the residents.

I Yeah, and then just specifically with the milestones, how well do you think the milestones help you communicate how the resident is progressing and what they need to do to progress?

R Well, you're talking about – I mean, there are no milestones mentioned here on this form or –

I Well, the milestones – let me just pull it up here – the milestones is number –

R So yeah, sorry, the milestones, number 7, yes, they are milestones, okay, well that's exactly what I was criticizing (laughs).

I Okay, that specifically.

R Sorry yeah, I was talking about this. These are a new things, these milestones.

I Yes.

R I mean, it used to be very similar except for --- these are the things I'm criticizing - that they are not applicable to every case, and this form does not allow you to skip one or two. You have comment on all of them.

I So you don't feel like those milestones can accurately capture what feedback you want to provide?

R Yes, that's how I'm feeling, yes, exactly, exactly.

I Okay, and how do you feel feeling out the form, overall, helped or hindered teaching or patient care?

R The form had nothing to do with that.

I No?

R No, no.

I Yeah, well that's kinda all the questions I have for you. Do you have any other thoughts that you wanted to share?

R No, no, this is it. No, no, thanks for listening.

I Yeah, no, thank you for sharing your feedback. We really appreciate it.

R Okay.

I So we'll kinda take what you said into consideration, and hopefully use everyone's thoughts on the form to kind of revise it or see what we can do to improve, so we appreciate what you have to say.

R Yes, absolutely, thank you.

END 12:50

I	Since I don't have your written consent form, which you can feel free to just email me whenever you have a chance, I'll just ask you, do you consent to participate in this study?

R	Yes, I do.

I	This is interview FD1.76.P, and what I'll have you do is just kind of, looking at the form or thinking back to the form, just relive the moment you were filling it out, and if you can just describe if this encounter was more about direct observation of skills or about reviewing approach to diagnosis and management?

R	In terms of the form that I filled out for [name]?

I	Yes.

R	Yeah, so this – and, so I guess it was – I know I put here 'direct', but now that I recall it correctly, it was probably when I discussed these cases over the phone, on call, so that would probably be indirect observation.

I	What were you noticing, when you were observing or reviewing with the resident that led to your score?

R	So, I was, overall, just getting a sense of whether or not I felt that [name] was competent and was being able to relay pertinent information, and if I felt that she was safe, I guess. So, those would be sort of the most important things I was looking for, in terms of her ability to glean information, so speaks to her competency, and as long as she was sort of safe doing it, and that she could actually come up with a management plan on her own.

I	Okay, and what information did you use in making the decision to give the score that you did?

R	Like, what information from my encounter with her or -- ?

I	Yeah, like, to get to the score that you did, what information did you pull from to help inform that decision? Was it, like, listening to her? Was it watching her? Was it –

R	Yes, you know, this was all listening, over the phone.

I	Can you say a little bit more about that? What were some of the things that you were hearing for?

R	Yeah, so I guess, in terms of pertinent details, I was listening, for this particular scenario, first and foremost, were the patient's sort of stability, in terms of their

vital signs, and she started off with sort of clearing that, in terms of how the patient was doing from a human anatomy standpoint, and then (00:03:05), for me, pertinent positives and negatives, and since we were dealing with a case of altered level of consciousness and potential sepsis, I was (00:03:14) for sort of points regarding infectious symptoms and pertinent (00:03:25) such as (00:03:26) white count or, you know, chest x-ray results or urinalysis results, etc.

I And did the resident's performance earlier on have any impact on your score?

R Hmm, which was my previous interactions with them prior to completing the CPA?

I Yes.

R I guess, you know, I did interact with [name] before, and so, I guess, whenever you interact with someone beforehand, it always is going to, I think, alter how you perceive them in the future, and there was nothing really negative, in her case, and so I would imagine that, yes, having interacted with her probably did shape my evaluation of her, but not in a bad manner, I wouldn't say.

I Right, and what were you specific goals when you filled out this form?

R I guess my specific goals were just to establish whether or not I felt she was competent doing this task; and secondly, was she safe doing it, right, because some people, you could say that they're not maybe at that level of competency that you would like; however they're still safe in their approach, and in terms of the questions they ask you, in terms of the help they ask for, so competency and safety, as I stated initially, were the two things I was just trying to assess on this form.

I And what were you trying to communicate to her?

R So, I, after listening to her, in terms of communication, I asked for clarifications for certain points regarding certain points in the history and in terms of investigations, and communicated to her that I was, overall, happy with her proposed management plan, and there was, in this case, little for me to add.

I Okay, and how did this form help you with some of your goals?

R Let's see………..I think the form was relatively easy to fill out, and sort of broad stroke categories. Like, for example, number 7 with the milestones, they were fairly easy to sort of address. Either they achieved it or they needed work, and so I thought that was fairly straightforward to complete.

I Just adding to that, how do you think the presence of these new milestones impacted your assessment?

R Hmmm, you know, in this case, ‘cause I thought she did a great job overall, there was not much to differentiate for her, in terms of the milestones.

I Right.

R Right, but in other scenarios with the same learner or with other learners, I think it’s helpful when they haven’t sort of achieved everything, right, because then there’s nothing really to differentiate, in terms of what they can work on, yeah.

I And how did this form interfere with some of your goals?

R I don’t think it necessarily interfered with any of my goals.

I Okay, and then I just have one more question. How do you feel filling out this form, overall, helped or hindered teaching or patient care?

R It didn’t affect patient care at all.

I No?

R Like, in terms of, like, I don’t think it hindered patient care, by filling out the form, nor did it help patient care, by filling out the form. Now if you’re asking if the discussion itself with the resident helped patient care, well it did but it is not necessarily reflected in filling out the form.

I Okay, and what about in teaching – in helping or hindering teaching?

R I didn’t necessarily think that it, again, helped or hindered with respect to teaching, when I was on the phone with her, because it’s just something I filled out after the fact, so I didn’t really use this to, I guess, consciously assess, at the time when I was speaking with her, you know, did she meet all those criteria, but –

I Okay, alright, well that’s all I have for you. Is there anything you wanted to add?

R No.

I Okay, well thank you so much for taking time to chat with me and participating in the study. Feel free to send me your consent form whenever you have a minute, and we will send you the gift card at the end of the study.

END 09:26

FD1.80.P
12:48

I So this is interview FD1.80.P, and I'm just gonna ask you, do you consent to participate in this study?

R Yes.

I Alright, so just looking at the form you have now, if you can just kind of go back in time and relive the moment that you were filling it out, just describe if this encounter was more about direct observation of skills or about reviewing approach to diagnosis and management?

R It was direct – well, it was direct observation 'cause I went over it with the resident, after the resident had assessed the patient, so I didn't watch the resident doing the history and physical, but I did go over it with him afterwards, and then we went back down to see the patient.

I I see, okay.

R So I was – it was still direct, but it was not direct, in that I didn't go in there with him and watch the entire thing.

I Right.

R But we did go in and go over it, so it was – 'cause there's kinda direct, completely, and then there's direct, and then there's indirect, right?

I That makes sense, yup.

R And so this was just direct; it was not direct, complete.

I Okay, and what were you noticing, when you were observing or reviewing with the resident, that led to your score?

R Umm……let me just see what …..okay, so, with all these milestones here, so the resident was actually able to look after all of the appropriate things that I would expect at this level, for a resident to be able to do with this patient. So, this was a complicated patient. I actually prefer to explain. So, this was a complicated patient, for which it would even be a challenge to do a complete assessment and determination of next steps for a haematology resident, let alone an internal medicine R1, and so I thought he did a great job at figuring out what was going on and figuring out, basically, what should be done, but he did need some support through this because that would not be expected at an R1 level, and I didn't actually give him the opportunity to completely run the whole thing because I knew there were some things about this case that were beyond what he could possibly know, and so we talked about, and I explained things to him, and then he completed those tasks.

I	Okay, and what were your overriding concerns at that point?

R	Patient safety. This was a timely diagnosis. We had to make decisions and act upon things quickly. There were a lot of people to actually phone, and so, because there were two of us, I actually told him to phone some people and I phoned some other people, and that, of course, gets the job done quicker.

I	Um hmm, and what information did you use in making your decision to give the score?

R	The resident's performance and what he was giving me back in terms of information and plan.

I	Okay, so what he was kind of presenting to you, yeah.

R	Telling me, yeah.

I	Um hmm, and did his performance earlier on have any impact on your score – like, early on -- ?

R	No, no, actually no.

I	Okay, so it was just this specific –

R	This particular resident, I hadn't – how can I say it? – I think his performance on this case was better than what – the performance I had seen previously, so no, I was not prejudging him.

I	Okay, and what were your specific goals when you filled out this form?

R	To fill out the form (laughs).

I	Yeah, to complete it.

R	No, I mean, he needs to get his EPA's done, and I told all the residents that, when they've seen something that fits one of the EPA's, they should tell me and we'll fill out a form.

I	Right, what you were trying to communicate with him on the form?

R	I was trying to communicate with him, and actually I put it more in the comment section here because I thought the actual check boxes were not as relevant to the particular case. So, actually, I think my comments are -- really where I wanted to go with this is that he recognized that this was emergent, and he didn't perseverate on finer details of the case. He actually got to the things that he

needed to do, talked to me, and we kinda came up with a management plan, and this is beyond – like, he really did need to talk to me about the case, and the fact that he recognized that was one of the things that he did very well is he didn't try and go off on his own on something that he really definitely needed my help on.

I	Yeah, okay, and how did this form help you with your goals to communicate that?

R	Actually, I thought the form was terrible for this particular case, which is why it's written kinda the way it is. I'm presuming you've seen the form, correct?

I	Yeah, I can see it.

R	You know, he actually did a really good job, but he – it doesn't actually kinda go over levels here, so I think he achieved things at the level I would expect for him, but he did not achieve things at the level that would be expected of a staff person looking after this case, so this was not a simple case. This was not somebody came in with pneumonia , where probably a resident at an R1 level could do – 'cause they're a good resident, they could do as well as a staff person, potentially. This is the kinda case where you really need to have additional training in order to do this. Now, this was the case that he saw, so given that he had to fill out EPA's, he's gonna have to do it on the cases that he saw, and it's not fair to say that he didn't achieve anything when he did really well for his level on this case.

I	Um hmm, it's not at the standard where he's at?

R	So, he's not at the standard where he could look after this patient independently, no way, no way, but nor should he be able to be at this stage. I would actually not expect any resident in internal medicine to be able to look after this patient, appropriately, even an R3, an R4. You really need to be either a haematology trainee – and this was a complicated case, so even those would need some help from staff, likely – or a staff haematologist, but if you're on the haematology rotation and you see this patient, and you've got these EPA's to fill out, this is the case you're gonna do it on, but the form didn't quite fit the case.

I	Okay, and you said, a little bit earlier, the milestones, yeah, they didn't really apply in this case.

R	They didn't and so I filled it out, but it's – you know, I think he achieved what I expected him to achieve.

I	Okay, so how well do you think the milestones helped you to actually communicate with the resident how he was progressing and what he needed to do to progress?

R I actually think this was – I would say this was a quite useless form for what I was trying to communicate.

I Yeah.

R Which is why, like I said, I think the comments are actually more important.

I I see, okay, and just how do you feel filling out this form, overall, helped or hindered teaching or patient care?

R I don't really think it did –

I Help or hinder?

R -- anything to – yeah, I can't say that it was really all that helpful. I think this was a case of it was a great learning opportunity for the resident. It got to show me how well he can make good decisions, to recognize his limitations, and to recognize emergent situations and what had to be done. That's actually what I got out of the case with the resident, but none of that is kind of on the form.

I Yeah, okay.

R Like, his initial management plan was – you know, just to give an example – I'm just kinda looking down at that one – so 'develop and implement initial management plan'. His initial management plan was to assess the patient as quickly as possible, figure out what was going on, and then go and get help, and he did that, and if he was an internist – a trained internist – with a case like that, that would be what I would be expecting. You know, an internist would have to actually get a hold of a haematologist and figure out what to do, and do it quickly, so he did do what would be expected of an internist.

I Gotcha, okay.

R I think the forms are a little bit trickier when it's a sub-speciality, you know?

I Yeah, it doesn't really apply.

R It doesn't really apply, always. I mean, sometimes the patient has more of a medicine – basic medicine problem, and it doesn't really matter what their sub-specialty issue is, but in this case it did, and so cases like this, it doesn't really fit, and because residents are on call, and, you know, the cases come up that come up, it's not fair to penalize the resident and say, 'Well we can't fill out this form' just because you had the misfortune of not having a case that was really applicable to the form.

I Um hmm, okay, well that's all the questions I have for you. Is there anything else you wanted to add about the form or your experience filling it out?

R …..I think – yeah, I hope that I've actually communicated to you what they issue is. There almost needs to be another category or something to communicate that the milestone is achieved at the level that would be expected of internal medicine versus sub-speciality, when it's sub-specialty.

I It needs to be differentiated at levels.

R It does, it does.

I Okay, I hear what you're saying, okay.

R 'Cause or else the residents are never gonna pass (laughs). Like, they're gonna be kinda stuck with not being – not doing perfectly on a case that there's no way they're gonna be perfect on, or else they're not gonna be able – we're not gonna be able to fill these things out, and they're not gonna get the EPA's done.

I Right, okay, was there anything else you wanted to add?

R I don't think so.

I Okay, well thank you so much for chatting with me today and sharing your thoughts. We appreciate you participating in the research, and hopefully we can use it to revise the forms.

R Okay.

I Perfect, take care.

R Okay, bye.

I Bye

END 12:48

FD1.62.P
08:41

I So, this is interview FD1.62.P, and what I'll have you do is, just thinking back on the form, just relive the moment that you were filling it out, as though it was on video, so just kind go back to that time, basically.

R Okay.

I So, just kind of remember that time, and so can you describe if this encounter was more about direct observation of skills or about reviewing approach to diagnosis and management?

R So, it was reviewing and approach to a case, and I believe I filled out multiple forms for this resident, and another one was on goals of care discussion and things like that.

I Okay, yeah, this one – specially the one with the milestones boxes.

R Okay, yeah.

I Um hmm, so that was reviewing approach, then?

R Yes.

I Okay, and what were you noticing, when you were reviewing with the resident, that led to your score?

R Well, I just – I thought he had a – sort of a very complete assessment of the patient. There was a very chronologic history; there was a good detailed physical exam, focussing on all the pertinent positives and negatives, and there was a nice summary of the case and breakdown of the issues, and organized approach to work through the differential diagnosis, the required investigations, and then – and basically a management plan, and I thought, in this case, everything was really complete, and I was impressed with it, especially for his level of training, and I believe I gave him the top marks on this exam.

I Um hmm, yeah, you did. What information did you use in making the decision to give the score?

R Well, I filled out the score. I was on call with him overnight, and the case was originally reviewed over the phone, and then I reviewed the patient myself in the morning, and then I instructed him to send me a form or mini checks or whatever the equivalent is, and I think it was two or three days later that I actually filled out the form, and so I used basically his approach to the case over the phone, and then my assessment the next morning.

I Did the resident's performance earlier on have any impact on your score, or was that assessment just based on this specific interaction?

R This was my first time ever on call with him, and I had never met him or worked with him before.

I I see, okay, and what were your specific goals when you filled out this form?

R Well, I just wanted to try and give useable feedback, and I always try and remember specifics about the case and try and include that in the text boxes. I find the wording a little bit maybe confusing in terms of between, you know, the resident can manage a case like this completely on their own. I just find sometimes the resident is maybe pretty close, and I almost want to give them that five level, but the wording sounds more like they should be in the four level. I find that hard when I review some of these cases with a resident, in terms of where to exactly put them, but –

I Okay, so, in that case, do you kind of defer to the four then, usually?

R No, I would say, usually I push them to the five. I think I just say it's close enough, and that's just my interpretation of the scale.

I Right, okay. How did this form help you with these goals to kind of give that feedback, like you were saying?

R Well, I think it is – it just allows for the opportunity to give some feedback, and I like it how the comments are mandatory and you have to put in some sort of comments, and I always think, hopefully, those are gonna – the resident's gonna read those, and hopefully they're gonna use that section. The overall scale at the top, I don't really find that too useful, and like I told you, I sort of interpret what the meaning is behind that.

I How do you think this form might have interfered with some of those goals, if at all?

R I dunno, I think it's fine. I think it's just a medium for feedback.

I Okay, and I know you didn't use the milestones in this case 'cause you scored him in the five, but how do you think the presence of these new milestones impacted your assessment?

R Can you remind me what the milestones are?

I sure, I'll just pull it up right now.

R I think there's, like, eight different categories, and you have to check off which one they didn't quite meet.

I Right, but if you score him in the five, then you don't have to fill out the milestones – or, the four, sorry. So, you had given him the 'can diagnose/mange this case with no significant change'

R Yes.

I And so, because you selected that, it says you can skip over the milestones, so the milestones is looking at things like recognizing urgent problems that need involvement of more experienced colleagues, or performing complete and appropriate assessment of patients, generating differential diagnosis, and it gives you two boxes, so you score them in progress or area on which to focus, or achieved.

R I see.

I So you didn't have to do it, but I was just curious in your opinion if it impacted your assessment or anything like that?

R No, I mean, I wouldn't just give someone the top mark to avoid filling that out. Yeah, I think it – maybe it's a little bit limiting. Maybe there should be kind of a text box in that area if there is another – something that falls outside of those boxes that you'd want to comment on, but other than that, I think it's fine.

I Okay, and how do you feel filling out this form, overall, helped or hindered teaching of patient care?

R I don't think it has any affect on patient care. I was able to fill it out on mail at home, at the end of the day, so yeah, I wasn't too concerned about that.

I Okay, that's all the questions I have for you, but is there anything else you wanted to add?

R No, I think that's everything.

I Alright, well thank you for taking your time to chat with me today. We appreciate you participating, and feel free to just send me the consent form whenever that's convenient for you, and we'll send your ten dollar gift card to some nurses or some support staff, right?

R Perfect, yeah, that's great.

I Okay, great, thank you so much.

R Will you be contacting me about another one in the future, or is this everything?

I I think this is everything that I’m a part of. I don’t know what Dr Daniels has in store, but this is all that I know of right now.

R Okay, thanks very much.

END 08:41

FD1.12.R
12:02

I So this is interview FD1.12.R. So, I'd like you to just think back to or look at the form, and try to relive or visualize the moment that your preceptor was filling in the form, so just put yourself back in that position. Please describe if this encounter was more about direct observational skills – sorry, direct observation of your skills or about reviewing your approach to diagnosis and management.

R I guess I would say, in this situation, it was both. So, what had happened is I had admitted that patient overnight, independently, then doctor [name] and I gone back to examine the patient together and to confirm the history and the physical exam findings, and then we reviewed the diagnostic approach afterwards, so, you know, I would say that Dr [name] used the form to guide what to look for when reviewing me or observing me examine and present the patient, and also used it kinda to guide how to evaluate my diagnostic approach to the issues.

I Okay, thank you. What do you think your preceptor was noticing when they observed you or reviewed you that let to their score?

R Well, I think in this situation, one of the things that was really important in the evaluation, to my preceptor, was just the approach to problem, and there was a patient multiple internal medicine issues, and all of which, kind of, in their own right, prompted the need for it's own evaluation. So, I found the from helpful in that I recall her looking through the milestones kind of as we talked about each issue that the patient had been there for, and it triggered conversation between her and I about how I evaluate in terms of working up each issue.

I Okay. What information do you think the preceptor used in making the decision to give you this score? So what were some of the evidence or the information they would have used?

R So, I think the first bit of information she used was the objectives, information that she had in front of her, then confirming what I had found on physical exam and on labs and on history to guide what I had done in terms of management. She actually, as part of reviewing, would often write out on an order sheet things that she would order kind of as she was thinking about the story just to prompt her memory and make sure we weren't missing anything when we reviewed, and then we kind of compared our order sets at the end, and she used that to see that there weren't really any deficiencies, and what I had done compared to what she would have done.

I Okay, great. Do you think that she used only this interaction, or do you think that she used other observations from that day?

R I think that, in this case, she did just focus on this interaction, but I could see how, if somebody's filling out multiple EPA's in a day, kind of your overall impression of your resident, how well they performed could bleed over into other

interaction, but I think these milestone bit was better than the – as the one that existed previously with no milestones does is that kind of prompt the examiner or the observer to think through all the points that their supposed to evaluate the resident on, rather than just kinda say, 'oh, you know what, they've been doing a good job today, so that's 5/5' or this one is doing pretty well today, so that's 4/5', if that makes sense.

I Right, yeah, that makes sense, thank you. What would you say was your specific goal in getting this form filled out?

R So, it was for me, right now, somewhat two-fold. One is just documentation and specific feedback related to specific cases, and how I was able to manage them; and then, two is, professionally and for the purpose of completing EPA's and a portfolio.

I Okay. And what do you think would have been your preceptor's goals in filling out this form?

R So, I think, for this particular preceptor, one of her goals was to support me and my learning as a resident, and she was very intentional about wanting to provide me with feedback, and just said to me, then, at the beginning of the shift, that 'Let's take the opportunity to do some EPA's today, specifically probably the FD1 EPA's, essentially, where you can admit the patients independently, and I'll review how you did with that', so I thought it provided her structure in what to look for when working with me that day, and that, yeah, it was essentially to support my learning and provide me with feedback.

I Okay, so you do you think that the EPA forms would have helped you with your and your preceptor's goals, or in what ways do you think they would have helped?

R I think it just kinda provides a more structured way for a preceptor to give you feedback every time, just kinda like thinking about other examples, in general, I guess, before they – or in circumstances where you're not filling out an EPA with a preceptor, they might just say, 'Okay, yeah, the job, that was good', and kinda leave it there, but then having the form in front of them prompts them to say, 'Oh yeah, I think that you did this appropriately', and kinda talk through some of the milestones. Whereas, previously, when I have admitted patients or I'm reviewing a case, the questions about what I had done, we had one this morning that I didn't get an EPA for, and my preceptor just said, 'Okay, good job', and I had to prompt them and ask, 'What would you have done in this situation?', otherwise they wouldn't have given me that feedback, but they may have done that if the form was in front of them, yeah.

I Okay, so they give you – it's a little just a bit more structured and maybe concrete and specific way of receiving feedback?

R Yeah, well I think there's just a little bit more robust feedback, yeah.

I Perfect, thank you. How do you think this form interfered with your and your preceptor's goals? Is there anything that you didn't like about them or that you thought maybe got in the way of some of your goals?

R I think the form itself is pretty easy to use, and my preceptor commented on that. I guess I find, for whatever reason, on my mobile device it's not the easiest to use, so I'm kind of always have to find a computer to pull things up on, so there's that issue. Not in this particular case but in other cases, I think preceptors kind of get evaluation fatigue, and some of them, I feel like, don't even really read the milestones. They just have their overall impression that, like, this was good, or else, you know, they kinda look at the length of the form and go like, 'Ahhh, yeah, no, that's good 5/5'

I Right, 'cause they don't want to fill it out, yeah, extra milestones.

R Exactly, so, in that case, if they don't kinda – I don't know if it's just because they don't want to fill them out or because they're truly satisfied, the feedback maybe kind of just the same as it would have been without the form, where they think you did a good job, and then essentially write, 'good job' and then ways to improve – "no ways to improve" (laughs).

I Right, okay.

R Yeah.

I Can I ask what kind of mobile device you use

R It's a Samsung Galaxy.

I Okay, I'm just curious if –

R Yeah, I find it –

I Go ahead, sorry.

R I find the forms are crashing or I can't pull up the portfolio properly on them, or there's a lot of lag time on the screen. That just makes them less user friendly.

I Okay, that's good information, thank you.

R Yeah.

I How do you think your preceptor would feel about how filling out this form, overall, helped or hindered teaching and patient care?

R I'm not sure. I think that's a difficult question. This particular preceptor at least took an interest in filling out the forms, and saw them not just as another hoop to jump through as a preceptor, but did see them as a chance to provide additional feedback and support the learning of the resident, and it did also prompt discussion between her and I about documentation issues, furthering practice, and keeping up with your professional education. It was useful in that regard too – kind of letting her see me more as a junior colleague, yeah, it's kind of using it to build their professional portfolio, if that makes sense.

I For sure, that's a good answer, thank you. That's all the specific questions I have for you. Is there anything else that you can think of that you'd like to add about the forms or on this particular EPA?

R No, not in general.

I Okay, well thank you very much for chatting with me today. We're gonna send out gift cards once the study is finished, so I'm not exactly sure when that specifically will be, but once all of our interviews are finished, then we're gonna send out the gift cards, so keep an eye out for that.

R Okay.

I And I already have your signed consent, correct?

R Correct.

I Right, you sent that to me yesterday. Okay, thank you so much, [name]

R Yeah, you're welcome.

I Have a good day.

R You too.

END 12:02

I So this is interview FD1.13.P, so [name] do I have your consent to participate?

R Yes, you do.

I Excellent. Can you hear me okay?

R Yes, I can.

I Okay, perfect. Okay, so I'd just like you to think back, or looking at the form, try to relive or visualize the moment that you were filling in this form for the resident.

R Okay.

I So I'd like you to describe to me if this encounter was more about direct observation of skills or about reviewing the approach to diagnosis and management?

R I'd say it was more about reviewing the approach to diagnosis and management.

I Okay, what were you noticing when you were observing or reviewing with the resident that led you to your score?

R I'm sorry, could you repeat that again?

I Sure. What were you noticing when you were observing or reviewing with the resident that led to your score? What led up to your decision to choose it?

R Oh, okay. So, I think the main thing was – I mean, I didn't really notice at the time that I was reviewing with the resident; moreso when I was completing the form – was, as I was completing the form, I realized that I didn't make any significant modifications to the resident. He reviewed the case with me; we examined the patient and reviewed his orders, and essentially, we just continued on with his plan without any modification.

I Okay. And so how would you summarize the situation at that point?

R The point that – at that point when I was reviewing with the resident?

I Um hmm.

R Yeah, I suppose the situation was best summarized as, you know, it was two things. It was, from the resident's perspective, it was probably a learning opportunity and a practice point in as much as he had this rather complicated patient that he had to deal with, and he did deal with appropriately. From my point of view, it was. Interestingly enough, it was a busy night on call. There was

a lot of patients to see, a lot of patients to take care of, and I guess I noticed that the resident was, on a service perspective, helping get all of the work that needed to be done, done, and we were focussed. Because he was so – he had demonstrated competency in looking after this patient, we were able to focus more simply on getting the work completed, rather than having to review or change plans in great detail. I don't know if that makes any sense or not, but –

I Yeah, it does, thank you.

R Yeah.

I And what information did you use in making this decision to give this score? So what was some of the evidence in terms of what the resident was doing or saying or portraying?

R So, the information, I guess, was compiled from what the resident told me himself, from what I had heard from the emergency physician who referred the case to us - because the emergency physician had called me directly about this case - and I had heard what the emergency physician's understanding of the case was, so I was able to kind of correlate that with what the resident was telling me, as well as what I discovered when I spoke to the patient myself and looked on Netcare. So I supposed I used the other sources of information to kinda double check with the resident.

I Okay, thank you, and did the resident's performance earlier on have any impact on your score?

R In earlier cases prior to this case?

I Yeah, or earlier in the day. Either way.

R Yeah, it – well, that's a good question (laughs). It maybe did. I wasn't aware of it, but he's a strong resident, for sure, so that, I suppose, might have, as I was talking to the resident, therefore given him more credibility than I would have had he been a weak resident.

I Right.

R Yeah, so possibly.

I That makes sense. Just a few more questions. What were your specific goals when you filled out this form? What were you hoping to accomplish with it?

R (laughs) I think I was just hoping to – in all honesty, there was a number of forms I had to complete. I had a number of EPA's sent to me by a number of different residents, and I was just hoping to get them crossed off my to do list (laughs)

I Absolutely.

R Yeah.

I Okay, how do you found that this form helped you with these goals? What did you – I guess, what did you like about the form?

R I did like how as in this particular case, I thought the resident was competent with no significant change to the plan, that I didn't have to go through each individual question and ask them individually, so that made it more time efficient, from my perspective.

I Okay, and how would you say that the form interfered with these goals?

R One thing that I've noticed on this form and with other EPA forms is that, even if the resident has performed at the highest level, you're still forced to type in at least one area for improvement, which I find sometimes artificial because if think they've, overall, done very well, it's hard to identify an area for --

I Right, so you're having to come up with things that you might not otherwise have indicated?

R Correct, yeah, so, you know, if I thought overall that the resident can diagnose and manage the case without any significant changes, then it's hard to think, for this particular case, that there is – it's hard to think of at least one area for improvement with this particular case, yeah.

I Okay, thank you. So you didn't use the milestones in this particular case. I'm wondering how you think the presence of these new milestones impacted your assessment.

R Sorry, there was just an announcement on the overhead PA system when you were saying that, so I couldn't hear the question.

I So you didn't use the milestones in this particular EPA, but how do you think the presence of these new milestones impacted your assessment?

R I think the presence of the milestones does give the form a rather somewhat intimidating appearance, and the thing I mentioned earlier about it being a strength where, if the resident scores in the highest level, you don't have to complete all of the milestones, might tempt some people to maybe just choose that as an easy way out, so they'll say that the resident did at the highest level, so you don't have to go through all of the individual questions and answer them individually.

I Right.

R Yeah.

I That makes sense.

R Yeah.

I My last question is, how do you feel filling out this form, overall, helped or hindered teaching or patient care?

R I think I – in terms of patient care, I'm neutral. I don't think it has an impact on patient care. In terms of teaching, I think there is a potential for more immediate feedback to learners, so they can get the feedback on a more timely basis, and have the feedback given to them in the context of the case, which I think could be more valuable. Now that's, I guess, more for the overall EPA process itself, rather than for this specific form, but I guess that's the one thought I have about that.

I Okay, thanks so much. Is there anything else that we didn't cover that you'd like to add?

R Nothing I can think of, no.

I Okay, excellent, well thank you very much for agreeing to chat with me today, and again, we'll send out gift cards once the study's finished, and if you could just send me the signed consent when you get a chance, in – I guess it's – is it Friday today? – I guess if you have any time today or early next week, that would be great.

R Absolutely, will do.

I Okay, thank you so much.

R Okay, you're welcome.

I Okay, have a good day.

R You too, bye bye

I Bye

END 11:23

FD1.46.P
10:37

I This is interview FD1.46.P. Do I have your consent to participate in this research, [name]?

R You do, yes.

I Excellent. Okay, so I'd just like you to, thinking back or looking at the form in from of you, try to relive or visualize the moment that you were filling out this form, okay?

R Yeah.

I So I'd like you to describe if this encounter was more about direct observation of skills or about reviewing approach to diagnosis and management?

R More about – I didn't – it wasn't a direct observation, so it's more approach to sort of investigations and management.

I Okay, thank you, and what were you noticing when you were observing or reviewing with the resident that led to your score?

R Their clinical, sort of, like- this is hard question. Sort of – you're kind of integrating their clinical knowledge, you know, sort of assessing their clinical knowledge their ability to decision make, to then decide the appropriate management. So you're looking at their clinical, sort of, knowledge base, their decision making and judgement, I guess.

I Okay, and how would you summarize the situation, at this point, when you were filling out the form?

R What do you mean exactly?

I I guess, sort of what was going on in the situation. How would you summarize how the resident – what the resident was doing and what they were saying?

R I don't – sorry, I don't quite understand the question. So, you want a summary of what the case was or -- ?

I Sure, sort of –

R Sorry, so basically it was an 80 year old gentleman who had acute shortness of breath that deteriorated in the hospital, and he sort of required medical intervention and sort of a MET call, and actually a bit of ICU stay, so sort of approach was the recognition of her – sort of, the clinical deterioration in the patient, and how – and sort of her judgement in how – sort of the severity of the situation, and sort of the need for additional help and intervention.

I	Okay, thank you, and what information did you use in making this decision to give this score? What was some of the evidence that led you to rate this particular resident in this way?

R	My encounter with the resident, sort of the history, her presentation, her clinical presentation and summary of the case, my clinical assessment, and sort of the result – investigations results as well.

I	Okay, thank you, and did the resident's performance earlier on have any impact on your score?

R	Sort of early on before – I only had one encounter with her.

I	This was your first encounter?

R	Yeah, yeah, I've only had – yup.

I	Okay, that answers the question. What were some of your specific goals when you filled out this form? What were you hoping to accomplish?

R	Well I guess it was just to sort of formally document a resident, sort of assess a resident's sort of clinical assessment, and approach to investigation and management.

I	Okay, so what were you trying to communicate to the resident in filling out this form?

R	Yeah, so I guess was to communicate sort of their current – sort of their current clin – my observation of their level of clinical functioning and sort of ability.

I	Okay, great. How did this form help you with these goals

R	I like some of the breakdown of the questions, so some of the detail questions – so, what type of presentation, the case description, the encounter, who they – sort of the rank of the supervisor. You know, it's quite detailed, quite specific, which I think is good for sort of resident learning, you know. I like the milestones, but I found them quite vague.

I	Okay.

R	No, maybe vague's not the right term. A bit too broad. Like, either in-progress, or achieved.

I	Right.

R I guess that's how EPA's are looking now, but I think it's a scale; it's not that clear cut.

I Right, so did you find the milestones themselves, vague, or did you find the actual scale for rating the milestones, vague?

R So it's more like – the milestones themselves were fine. It's just the scales. So, for instance, in the part of above the milestones you have an area sort of four choices for assessing, diagnosing, initiate and management for patients with common acute medical presentations, and there's four options for the answers, so that provides more leeway I think it's easier to actually determine at what level the resident is.

I Right.

R But then, the milestones, it's literally broken into two broad categories, and often you don't always – and also, the other thing is the milestones is you can encounter -- you might not be able to answer all of these milestones, so I think that's sort of one of my limitations, especially if you only work with the resident once or twice, it's hard to answer the milestones.

I Okay, thank you. How do you think the presence of these milestones impacted your assessment?

R I think their presence is good. It gives you sort of the objective to focus on, you know, when you're doing the assessment, so I like the presence of them.

I Okay.

R I just maybe – I just think you just can't answer all of them for a single encounter. It's more if you have multiple encounters with the resident where you can address the milestones 'cause not all encounters you'll be able to answer all the milestone.

I Okay, thank you, and how do you feel that filling out this form, overall, helped or hindered teaching or patient care?

R Oh, I think it would have sort of helped patient care 'cause it would allow recognition of any significant issues in the resident's performance. It would allow that recognition, things that they need to work on – you know, deficiencies – and the same it also allows recognition of something that the resident is doing well and they need to keep doing, so I think, overall, it's very positive.

I Okay, great. Those are all of the specific questions I have for you, but is there anything else you'd like to add that we haven't talked about already?

R I don’t think so. I think, just for me, (laughs) I’m going on about the milestones. I think it’s easier if you’re doing this after you’ve known the resident and you’ve had many encounters with them, but for a one off encounter, you can’t – whether it can be tailored more – or an extra answer where it’s not observed, rather than just the two categories for each of the milestones. I think that’s my main limitation.

I Okay, so like an option to say ‘not applicable’ for instance?

R Yes, yes.

I Okay, that’s really useful information. Thank you. Okay, so that’s all that I have for you today. Thank you very much for taking the time to chat with me. If you could, in the next couple of days, ideally, just print, sign and email me your consent form, that would be wonderful.

R Yeah, I’ll get that done. Sorry about that.

I No problem. And once we’re completely done the interviews, we’re going to be sending out all of the gift cards, so keep an eye out for that.

R Okay, no worries.

I Okay, thank you so much.

R Thank you, [name], cheers.

I Have a nice day.

R You too, bye bye

I Bye

END 10:37

FD1.54.R
08:22

I So this is interview FD1.54.R. So I'd like you to just sort of think back, or looking at the form in front of you, try to relive or visualize the moment that your preceptor was filling out this form. Just sort of put yourself back into that headspace.

R Okay.

I Okay, so I'd like you to describe to me, first, if this was more about direct observation of your skills or about reviewing your approach to diagnosis and management.

R I would say the latter – reviewing my approach, yeah.

I The latter, okay, thank you. What do you think your preceptor was noticing when they were reviewing you that led to their score?

R That led to the score that they gave me?

I Um hmm.

R I think probably a combination of the thoroughness of the written note, and a three line summary outlining the case. I think those were the two big things.

I Okay, so what information do you think that they would have used in making the decision to give you this score? What was some of the evidence would have prompted them to give you the score that they did?

R I think it's, 1) if they agree with the diagnosis; 2) if they agree with the initial management plan that I've suggested, and I think I think the third thing, which s probably the main one, is how much does any change that they would make to my management plan – how much – yeah, how much would they alter my management plan that I came up with, in terms of evaluating how correct they thought it was. I don't know if that made sense.

I Okay, so I'm gonna turn your volume up a little bit. What was your specific goal when getting this form filled out? What were you hoping to accomplish?

R Well, I mean, it's a requirement.

I Pardon?

R It's a requirement, but my specific goal was to make sure that someone else was confident in my ability to manage a patient with altered level of consciousness.

I Okay, thank you. What do you think your preceptor's goal was when filling out this form?

R I think their goal was to provide some specific feedback for a specific case that we saw together.

I Okay, and how do you find that this form helped you and your preceptor meet these goals?

R Well, I'd say a written evaluation that I can refer to at a later point in time, and I also think that, you know, it forces each of us to reflect on how the patient encounter went, and in doing so, I think it sort of reinforces whether we feel that our learning goals or objectives have been met with this particular case, or whether there's still a gap that needs to be filled.

I Okay, perfect, and how would you say that the form interfered with some of these goals? Is there anything that you…?

R Yeah, I mean, I would say, sometimes I find that we structure our approach for presentation to a staff member based on the criteria outlined in the EPA itself, as opposed to what might be practical in a given situation. So, for example, if it's a very busy call or if there's a lot of consults attending, sometimes it's not always practical to sort of go through all, sort of, tick boxes that would be expected that would be expected to be captured by an EPA.

I Right, so on the one hand, it's nice that it's specific, but then sometimes it's maybe too narrow or too specific?

R Yeah, well just, like, I liken to almost to an OSCE where if we didn't –

I Sorry?

R We had an OSCE last week, actually, and if we didn't articulate specific items, like, for example, if a patient comes in and if you haven't asked about this, or haven't asked about this, or haven't asked about this, you don't get a score for it, but you may have thought about it, but because you didn't specifically articulate or think it was relevant to the specific sort of summary that you needed to give them, that sometimes changes their perception of it. So, what I'm saying is, with the EPA, sometimes that it's sort of looking for specific things, and when the preceptor goes through it and says have you met the specific criteria, then click yes, that was achieved. Well, I sometimes find it's very – it very much influences how they maybe perceive the encounter went.

I Hmm, okay, that makes sense, thank you. The last question I have for you is how do you think your preceptor would feel about how filling out this form, overall, helped or hindered teaching and patient care?

R I think, in principle, they would probably say it's a good idea and was helpful. The challenge I find on an ongoing basis is it often doesn't seem to be the appropriate time to do it right in the moment, and it's often done at a later point, so sometimes they might feel that it hinders the patient flow or the work flow of a particular day.

I Okay. Thank you very much. Is there anything else that you'd like to add that we haven't already talked about?

R For how EPA's work in general, or just –

I Yeah, sure, either this EPA or EPA's, in general, if you have any feedback or thoughts.

R Well, I mean, the couple feedbacks I guess I would say is – and I know the staff in our program is working on it – but I still think a lot of the staff are either unaware or don't make it a point, on each rotation, for a couple of EPA's to be completed for each resident. I find it's often very much us going to them and asking for it, as opposed to it maybe going both ways. And then the only other thing I would mention – I think it's really good – I've noticed in the elevators there's now posters about how EPA's are now a new thing, but the website link has now changed that they have not yet updated all the posters. That's a big deal, but that's all I would say, but other than that, I think, in principle, it's actually a very good idea, and I think, the more we do it, the more people will get used to filling them out.

I Okay, great, thank you very much, [name]. So, you've already sent your consent form. The way that this works is that, when all of the interviews are completed, we're going to send out the gift cards.

R Okay.

I So just keep an eye out for that, and yeah, thank you very much for taking the time out of your day to chat with me. I really appreciate it

R No problem. I'm happy to help.

I Okay, have a good day.

R You too.

I Bye

R Bye **END 08:22**

I Alright, this is interview code CD1.74.R. Alright, so thinking back or looking at the form in front of you, I'd like you to try to relive or visualize the moment that your preceptor was filling in this form.

R Okay, well actually I emailed it to my preceptor, so –

I Okay, so just kind of get yourself in that headspace for the next couple of questions, okay?

R Okay.

I I'd like you describe if this encounter was more about direct observation of your skills or about reviewing your approach to diagnosis and management?

R It was reviewing approach. It was not direct, so indirect, afterwards.

I Okay, so reviewing your approach.

R Yeah.

I What do you think that your preceptor was noticing when they reviewed you that led to their score?

R What they noticed while reviewing?

I Um hmm.

R Yeah, so I think particular EPA, I missed a point on that, so the patient was hypotensive, and I think that was by EMS notes, but then we were looking at the chart, couldn't find the EMS notes, and then this was not – so this information was not told to us, so then we were not sure, and I think that was the main point because was previously on steroids, and then apparently she was initially hypotensive, but receive – which resolved after having some IV fluids by EMS, but during her stay in emerg – almost for two days – she was stable, and the main point that I got was, you know, trying to look at the EMS notes as well, and also – and think about adrenal sufficiency if you have somebody on prednisone taper in the past.

I Okay, thank you. So what would have been your preceptor's sort of overriding concerns at that point?

R Overriding concerns? I think it was mostly trying to – making the point that – to consider adrenal insufficiency, secondary adrenal insufficiency if you have somebody on prednisone taper. I think that was the main point, and then also to trust your source as well because that was during my senior management shift,

so, you know, I trusted all the details from my junior. I asked about hemodynamic stability, I this fine? And then I saw some nursing notes, which is as per the nursing notes during the hospital and two days in emerg, and there was no hypotension documented, but I missed to also look at the EMS notes, and I just, you know – I think it's a lot information that I was given from my junior, yeah.

I Right, that makes sense, thank you. Do you think that your preceptor used this interaction only, or do you think that they might have used other observations from that day?

R Observation? So, you know, it was later on, so we already admitted the patient, and then the patient was in emerg when I was reviewing with her, and there was not direct observation of my – of my examination or history and taking and things like that. Now, I guess, later on we found the EMS notes, in the chart and I'm like, oh, at some point she was hypotensive; I didn't know that.

I Okay.

R Yeah.

I What was your specific goal when getting this form filled out? What were you hoping to accomplish?

R Actually, the first time, that was my staff who told me to send her an EPA (laughs). Usually I try to go after them, but this time it was my preceptor who told me to send them the EPA, so, and my goal is obviously to achieve my EPA targets by the end of the year, yeah.

I Okay, great. What do you think was your preceptor's goal when filling out this form?

R Feedback.

I Yeah, so to provide you with some feedback?

R Yeah, yeah.

I Okay, how do you think that this form helped you and with your preceptor's goals?

R I think it has helped, like, but more probably, like, more feedback. Because sometimes, you know, if I said we see patients, and then afterwards we don't know what happened, and sometimes we forget about it, and sometimes it's hard to get, like, on site feedback. Usually it's at the end of the week that you are reminded of some cases that you might have seen in the past or something to

include, but that would be, like, just on the spot, so, you know, things that you missed, those are things that you need to focus, and this is how you can improve, so I don't have to wait 'til the end of the week.

I Some more sort of direct and timely feedback for you?

R Yeah, yeah, yeah.

I Are there any ways that this form interferes with you and preceptor's goals? Maybe some things you didn't like or -- ?

R Interface with what, sorry?

I Are there any ways that the form interfered with you and your preceptor's goals? So, maybe things you didn't like about the form, or things that sort of got in the way of reaching your goals?

R No, I think it was fine.

I Yeah?

R It was a bit different from the old EPA's. I think now it has more detail, so –

I Right, the milestones

R Yeah.

I Okay, how do you think that your preceptor would feel about how filling out this form, overall, helped or hindered teaching and patient care?

R I think it, overall, helped.

I Yeah?

R Yeah.

I Okay. Can you say a little bit more about how you think the form helps teaching or patient care?

R I think, like, more focussing on the, as I said, milestones, so then the areas that I was good, the areas that I need to have improvement. Because, in the past, I think we had this just, you know, was there any changes, yes or no? Or, you know, I think it was from 1-5 or something like that, but did not have any milestones. So, now especially now she would be able to comment, you know, on other milestones; not just the management plan or the diagnosis, so I think it also has implications that is also helpful.

I Okay, perfect, thank you. Those are all the specific questions that I have for you, but is there anything else that you'd like to add today?

R No, I'm good, thank you.

I Okay, awesome. Like I said, we will send out the gift cards once the study is completed.

R Pardon me? Sorry, I couldn't understand that.

I That's okay, like I said, we'll send out the gift cards once the study is complete.

R Okay.

I Thank you so much for taking the time to talk with me.

R Okay, thank you, bye.

I Bye

END 08:01

FD1.46.R
11:46

I So this is interview code FD1.46.R. Okay, so thinking back, or looking at the form if you prefer, try to relive of visualize the moment that your preceptor was filling in the form. Just kind of take yourself back to that moment.

R So, actually, I emailed the preceptor, so wasn't there when she filled the form, but I think we went through it all in detail, how I manage the patient. I think she – I got her feedback from her over the phone, and she was very happy with how it was managed.

I Okay.

R I think she was kind of, yeah, confident of what she wrote.

I Okay, so was this encounter more about – so this would be more about reviewing your approach to diagnosis and management, as opposed to direct observation of your skills?

R Well it's kind of. She was on call at the university, and the patient was at the Royal Alex, so she couldn't make it to the Royal Alex, so I managed the patient on my own.

I Okay.

R And afterwards I reviewed it with her, so she was fine with… .

I Okay, thank you. What information do you think that the preceptor used in making the decision to give you this score?

R So, first of all, would be recognition of how sick the patient is when I called the preceptor initially.

I Um hmm.

R So I was doing a home call at night, and I had to come from home because I realized the patient is sick, although hemodynamics were stable, but I was concerned that the patient is going to progress poorly – this is number one. Number two would be how I manage the patient in terms of ordering preliminary investigation over the phone before I'm in house.

I Okay.

R And number three would be the outcome, so, in terms of patient safety and dealing with the family.

I Great, thank you. Do you think that your preceptor used only this interaction, or do you think they may have used other observations from that day, or other perceptions of you?

R I think that it wasn't a single decision or a single situation based decision because we had a few sick patients on the ward, and I was able to interact with the preceptor prior to this situation. I think she kind of felt that the background about me during – because I was covering the weekend and then this call, so, and she was kind of more confident of writing her feedback based on the previous attempt or previous situation.

I Okay, thank you, and what was your specific goal when getting this form filled out? What were you hoping to accomplish?

R Sorry?

I What was your goal when getting this form filled out? What were you hoping to accomplish?

R So, I was looking for anything in managing the patient, or if there's anything I can improve on – this is number one. Number two, just filling out more EPA's.

I Okay, great, and what do you think was your preceptor's goal when filling out this form?

R I think the preceptor was kind of trying to help with more EPA's for myself from her side, and she was just trying – you know, I felt over the phone when I just asked if I can send – and if I can send her an EPA on her email, she was very willing to do it. Like, she had that kind of – sort of deserved it because I managed the patient while she's in a different hospital.

I Okay, thank you. How do you think that this form helped you with you and your preceptor's goals?

R With my -- ?

I With these goals. How do you think that the form helped you?

R I think that it does actually when they write a comment – like their comment on how I managed the patients or how the patients are managed, in general, and what their expectation is about managing the patients because different staff have different expectations. So one of the staff would be more concerned about the medical/legal aspect and keeping the safety of the patient. One staff would be more concerned, more interested in seeing how good I approach the patients, and others would be just thinking about medications or my interaction with the nurses, and liked how I managing the patients. So, I've seen different – yeah, I've

seen different comments from different staff, so it’s very helpful to know what their expectations are.

I Okay, so it helps give you some sort of qualitative formative feedback on your performance, and then it also helps with understanding what different preceptors expectations are for you?

R Yeah, ‘cause if they look at the patient from different perspectives, yeah, some would – one of them would include all perspectives, as one would focus on one perspective, so they give you their feedback according to their expectations.

I Okay, perfect, thank you. How do you think that this form interfered with you and your preceptor’s goals?

R Interfered?

I Yeah, like what are some things that maybe you didn’t like about it, or got in the way of sort of reaching your goals of getting that feedback?

R So, for the form itself, the one with the milestones, I think the preceptors gets confused. If I get the highest score, they don’t have to fill out the seven questions underneath.

I Um hmm.

R So, but they do, so that makes it a longer form.

I Okay.

R It takes longer time to fill out the form.

I Right.

R That’s from the preceptor side. From my side, I didn’t have any problems filling out the form or sending the form.

I Okay, thank you.

R Yeah, it doesn’t take any time, yeah.

I I just have one more question for you. How do you think that your preceptor would feel about how filing out this form, overall, helped or hindered teaching and patient care?

R So, I usually get my forms filled out after managing the patient, so it does not interfere with the patient’s care.

I Um hmm.

R So, they usually fill it out maybe from a few days to a week after I send it.

I Okay.

R Some of them wouldn't find time to fill it out, so I have to send reminder emails.

I Right.

R And sometimes they do not respond even with reminder emails. So, it does not interfere with the patient's care, from my side, sending the form, and I think, from the preceptor's side too, replying to the form or filling out the form because they do it later when they have enough time.

I Okay, and how about with regards to teaching?

R Teaching, it's helpful for me to me what my weakness points are, so I was just trying to recall how the EPA – not specially this one – but, yeah, so when I started doing the EPA's, I remember one of the comments was to keep more broad differentials about the diagnosis.

I Um hmm.

R So I learned that, even if the patient is diagnosed with a certain diagnosis or as a certain disease, and he presented with representation related to this disease, I need to exclude other differentials.

I Right.

R So that's one of the things that I've learned from my EPA's, in terms of teaching.

I Okay, thank you very much.

R No problem.

I So I don't have anymore specific questions for you, but is there anything else you'd like to add that we haven't talked about already?

R Yeah, so, yeah, it's just sometimes it's hard to get hold of the preceptor, in terms of filling out the EPA's, so I usually, before sending the EPA's, I will let the preceptor know that I'm going to send off an EPA email, and that, but it ends up by not responding to the EPA's. I send the reminder emails, but sometimes they do not even respond to the reminder email.

I Okay.

R So, I get attempt lost because the staff are not responding or not filling out the form, and yeah, and yeah, and on some rotation we don't get the chance to fill out to do a lot of the EPA's. Like, I was endocrine, and there wasn't many patients, but most of the patients were on outpatient setting, so most of the EPA's are related to inpatients rather than outpatients. For a whole month or for a whole four weeks I couldn't do any EPA's.

I Hmm, okay, so it sounds like there's a few barriers to getting them filled out.

R Yeah, if we can get EPA's for patients we are seeing in the clinic or for outpatient that would be helpful too.

I Right, okay, that's very useful information, thank you so much.

R Thank you.

I Okay, so like I said, we are going to be sending out the gift cards once we've completed out interviews, so just keep an eye out for that.

R Thanks.

I Okay, thank you so much for chatting with me today.

R Thank you. Thanks for calling.

I You're welcome. Have a good day.

R Okay, you too, bye bye

I Bye

END 11:46

I Alright, this is interview code FDI.15.P. I'd like you to think back or look at the form and take a moment to relive or visualize the moment you were filing out this EPA.

R Okay

I Please describe if this encounter was more about direct observation of skills or about reviewing approach to diagnosis and management.

R Reviewing approach to diagnosis and management.

I What were you noticing when you were reviewing the resident that led to your score?

R I'm pretty sure they present the patient is a very organized and concise manner, and her management plan was complete and didn't need any or very minor modifications from my end.

I Okay, perfect, thank you. Did you find that the resident's performance earlier on had any impact on your score?

R This was the first time I was working with the resident.

I Okay, excellent, and what were some of your specific goals when you filled out this form? You've mentioned this already, but just for the audio.

R Yeah, it was mainly just to provide positive feedback to the student in the program. I thought she did a good job.

I Excellent, thank you, and then how did you find that the form helped you reach these goals?

R I mean, I think that I was able to express that she had a good approach to the program – sorry, to the problem – and that – that I didn't think that she needed much help in terms of her management plan. Yeah, that was – I think it asked all the necessary questions.

I Excellent. Were there any ways that this form interfered with these goals?

R No, I don't think it interfered with it. The only thing is, you know, it asked for one area for improvement. I mean ,she did a very good job, and I wasn't able to identify any areas where she could have improved, so yeah, I put 'none' or 'n/a/'or something like that, so as long as that's allowed. That was maybe the only thing.

I Um hmm, okay, and so you didn't use the milestones in this particular EPA, but how do you think the presence of these new milestones impacted your assessment?

R I don't think they impacted my assessment.

I No? Okay.

R Because I really didn't use the milestones.

I Okay, and then I just have one more specific question for you. How do you feel that filling out this form, overall, helped or hindered teaching or patient care?

R So, in this case, I don't think that it necessarily altered patient care, again, because I think she did a good job, which was reflected in her evaluation and in my completion of the form. I think, in other cases, it does prompt more immediate feedback to the resident. So, if there are areas for improvement, it just prompts that discussion right away.

I Okay, awesome. Is there anything else that we didn't cover that you would like to add that you think might be useful information for us?

R I don't think so.

I Okay, perfect, thank you.

R Okay.

I So you've already sent me the consent form, and we will send you a gift card when the study is complete. Thank you so much for chatting with me today, and sorry about the technological issues.

R Okay, no worries. I'm sure half of it was my cell phone. Okay, thank you.

I You're welcome, have a good day

R Okay, bye.

I Bye

R You too, bye.

END

FD1.63.R
07:48

I Okay, this is interview FD1.63.R. So I'd like you to just think back, or look a the form if you have it in front of you, and just try to relive or visualize the moment that your preceptor was filling in this particular EPA. Just sort of put yourself back in that position.

R Okay.

I Okay, I'd like you describe if this encounter was more about direct observation of your skills about reviewing your approach to diagnosis and management.

R Review my diagnosis to management.

I Okay, thank you. What do you think that your preceptor was noticing when they observed you – or, I guess, when they reviewed you – that led to their score?

R Can you repeat the question?

I What do you think that your preceptor was noticing when they reviewed you that led to their score?

R Well, for this specific EPA, I emailed the preceptor ask her is she is available with feedback from here, and then she filled it by her own.

I And then what, sorry?

R She filled the EPA by her own. I emailed her the form, and she completed it, I submitted it.

I Okay, so what do you think that – sort of what was some of the information that the preceptor would have used in making the decision to give you this score?

R I would say, like, my implementation of skills, my – how I went through the case, how I mentioned the relevant symptoms and relevant negative symptoms, how I interpreted by physical exam and initial lab work.

I Okay, great, thank you. Do you think that your preceptor used only this interaction, or do you think that they used other observations or impressions of you from that day?

R I get the feeling that the preceptor was taking into account your level of training, as well, during these encounters. Like, if you are year one or year two, that's slightly different in terms of their expectations and how much they would grade you.

I Okay, thank you. What was your specific goal when getting this form filled out? What were you hoping to accomplish?

R One is that I would say, every time I present a case or just an admission with my preceptor, either a staff or a subspecialty fellow, I would get some feedback from that, but given that we are required, through our training, to do these EPA's, that's one of the main purpose of doing the EPA.

I Okay, so meet the requirements?

R Yes

I Okay, thank you. What would you say that your preceptor's goal was when filling out this form?

R Say again.

I What would have been your preceptor's goal when filling out this form?

R I think that when I asked her to do the EPA, she had multiple encounters in the past, and she was aware that the requirement from the program, and it's only fair for me – fair to me that she do it.

I Okay. How would you say that this form helped you with you and your preceptor's goals? What do you like about it is maybe another way to think about the question.

R Yeah, so this form, I didn't do many of the milestone form yet.

I Right.

R Maybe the third or fourth one. I think it's make it easier to the preceptor who choose not to give you the full score to navigate through what area you can improve on.

I Okay, perfect, and in what ways do you think this form interfered with your and your preceptors goals? Maybe what are some things that you didn't like or that got in the way of reaching your goals?

R I can't think of anything (laughs). I think it's well written, like, the way that multiple domains that the preceptor would read through and decide.

I Okay.

R Yeah, I can't think there is something that can be added.

I	Okay, one more question for you. How do you think that your preceptor would feel about how filling out this form, overall, helped or hindered teaching or patient care?

R	Can you repeat the question again?

I	Sure. How do you think that your preceptor would feel about how filling out this form helped or hindered teaching or patient care?

R	I dunno (laughs). I don't have answer.

I	Is there a way that you think that the forms help or hinder teaching or patient care – your own teaching?

R	I would say maybe with subsequent forms, in terms of the reading the domains, and understanding that such aspects of – like, a teaching moment or teaching encounter, that you will pay attention next time about a specific encounter. Either way, like even if the staff wanted to give verbal feedback to a student or a resident, they would use these domains. I would say that's how the form helped, and so patient care, I'm not sure.

I	Okay, thank you, that's helpful. Is there anything else that you'd like to add about this EPA that we haven't talked about yet?

R	Yeah, I'm not sure how did they select the specific EPA? Was it by luck?

I	For this interview?

R	Yes.

I	So, basically, we're contacting – [name] is contacting residents and preceptors who are filling them out, and just sort of as they come in, and then people who are willing to speak with me, I contact and set up interviews with, so they're kind of just – a lot of people are being contacted for them.

R	Yeah.

I	Yeah, so we're just curious about how the new milestones – how preceptors and residents are feeling about the new milestones, more or less.

R	Yeah, okay, yeah, no, I don't have any further questions, no.

I	Perfect, thank you so much for taking the time to chat with me today, and we'll be sending out the gift cards once the study is finished, so just keep an eye out for that.

R Okay, thanks.

I Thank you so much.

R Okay, bye.

I Have a good day, bye.

R You too, bye.

END 07:48

FD1.22.R
8:05

I	Okay, so I'd just like you to think back, or looking at the form if you have it available, just try to relive or visualize the moment that your preceptor was filling out your form.

R	Okay.

I	So just kinda put yourself in that headspace.

R	Sure.

I	Can you describe whether this encounter was more about direct observation of your skills or about reviewing your approach to diagnosis and management

R	I guess, like, this particular one was a little bit of both because this preceptor came into the room, and sort of watched me review the case with the patient in front of me, so a little bit of both.

I	Okay, so they would have been observing you interact with the patient, though?

R	Hmm, no, it was more summarizing at that point.

I	Okay, perfect, thank you. What do you think your preceptor was noticing when they observed you or when they reviewed you that led to their score?

R	I mean, think they were probably looking for pertinent positives and negatives in the history, like trying to round out the history, look for anything that was missed, and then sort of develop a differential from that, and see if my differential matched up with theirs, if I missed anything important would probably be my guesses.

I	Okay, do you think that your preceptor had any overriding concerns at that point, or was it pretty positive, overall?

R	I mean, they didn't say they had any concerns in particular, so I think it went okay.

I	Okay, what information do you think the preceptor used in making the decision to give you this score? So what was the – I guess, some of the evidence?

R	I mean, I guess, like I can't pretend to know what they were thinking, but probably, you know, how much they felt they had to contribute in addition, or, like, was there any critical diagnoses that, like while we're not investigating, I didn't mention, but maybe it's oh, like, you know, I feel like, you know, we don't need to investigate this because X, Y, Z makes it a very low probability. So, I imagine they were sort of looking for investigating things that were still possible, or ruling out, based on history, to a reasonable asurity.

I Okay, thank you. Do you think that – this question may or not be applicable to you – but do you think that they would have used only this interaction, or do you think they used other observations of you from that day?

R Well, this was the only time I met the staff, so it was probably just this interaction.

I Just the one interaction, okay.

R Yeah.

I What was your specific goal when getting this form filled out?

R Pardon?

I What was your specific goal when getting this form filled out? What were you hoping to accomplish?

R Just get more EPA's.

I Just get more EPA's?

R Yeah.

I Okay, and how about your preceptor's goal? Do you think that their goals would have aligned, or if they would have had different goals?

R I mean, I think, you know, they probably want to, for participating in teaching, being in a teaching hospital on a CTU team, so I guess, you know, I feel like their goal would probably be to convey some teaching, and assess how I'm doing in terms of learning and being able to do cases like that by myself, or monitored, or, you know.

I Okay, and so what do you think your preceptor was trying to communicate to you through this form?

R Pardon?

I What would you say that your preceptor was trying to communicate to you through this form?

R Probably that they're happy with my approach, just keep reading around cases, just general sort of advice

I Okay, in what ways do you think that the form helped you with your and your preceptor's goals?

R Like I said, for my goal, letting me know that my approach is, you know, along the right lines in terms of seeing patients with that presentation, and considering the alternative diagnoses, and establishing initial manage work up. In terms of my preceptor's goal, I guess, like, their goal would be to further learning of residents, so it's difficult for me to talk to that, I guess.

I You're doing great. Just do your best with it.

R Pardon?

I Do you think that there's any way that the form interferes with these goals? , or anything you don't like maybe about the forms?

R I mean, I guess something that I always worry about is whether scores are higher because, like, it's in a barrier for – like, you know, it always makes me wonder, like, if you give the highest score, you don't have to then fill out all that, like, multiple milestones thing.

I Right.

R So then I wonder, you know, are they giving the highest score to avoid doing that, or did I truly do a good job, you know?

I Right.

R But then some staff are – will go down the checklist anyway, even if you get the highest score. So, for some, I'm not that concerned, but for others, you're like, oh, I wonder, but it's just a wonder, right. I'm sure they wouldn't do that out of laziness.

I Yeah, so in those situations, are you sort of hoping that you – or wishing that you had more feedback?

R I mean, I'm happy if they say they truly don't have any feedback, but, you know, I'm sure, like, everyone can't say that, right?

I Right, okay, and this is another one of those put yourself in your preceptor's shoes kind of questions, but how do you think that your preceptor would feel about how filling out this form, overall, helped or hindered teaching and/or patient care?

R I don't understand the question. Could you rephrase it?

I Sure. So, what I’m trying to get it is I’m curious to know how you think your preceptor would feel about how these forms help or hinder teaching and patient care?

R I mean, I think I would probably hope that it helped. I guess they too would, like – you know, I’m sure they have concerns that they’re maybe hindering or helping ,but I think everyone recognizes it’s a ongoing, evolving process to perfect the forms, right.

I Right, okay, thank you. I don’t have any other specific questions for you, but is there anything else that you’d like to add about this particular EPA or EPA’s in general?

R No, I don’t think so.

I Okay, perfect, and you’ve already sent me your consent form.

R Yup.

I So we are good on that front, and like I said, we’ll be sending out the gift cards once the study is finished, so just keep an eye out for that in your email

R Sure, okay, cool.

I Okay, thank you so much for chatting with me today.

R Oh, no, sorry for being so difficult to get a hold of on Friday.

I Oh, no problem, I understand. The life of a resident is very busy.

R Yeah.

I No worries. Okay, have a good day.

R You too.

I Bye

R Bye

END 08:05

I Alright, so this is interview code FD1.10.P. Do I have your verbal consent to participate?

R Yes.

I Excellent. Okay, so what I'd like to you do is to sort of get in the right head set for this, just sort of think back or look at the particular EPA that [name] sent you, and try to relive or visualize the moment that you were filling in this particular form.

R Okay.

I Okay, so I'd like you to describe if this encounter was more about direct observation of skills or about reviewing approach to diagnosis and management.

R This one was more about reviewing the direct management of the patient.

I Reviewing?

R Um hmm.

I Okay, thank you, and what were you noticing when you were observing – or I guess when you were reviewing with the resident that led to your score?

R Sorry, maybe I misspoke. I did actually directly observe her interaction with the patient in this case.

I Okay.

R So it was actually, I suppose, directly observing her management of the patient.

I Okay, excellent. So, when you were observing, what were you noticing or reviewing with the resident that led to your score?

R I was thinking, in my mind, what I would be doing in a similar scenario. I was thinking about things that I would be asking in that scenario for patient safety, or observing the patient's safety. I would be mindful of moving the interaction with the patient along at a speed to ensure that we quickly hit all of the points that we needed to, to be able to make a decision about present care.

I Okay, great, and did you use the milestones for this EPA?

R Yes, this one had the seven or eight item milestone checklist.

I Okay, and then, for this particular EPA, did you fill out individual milestones, or did you click, sort of, achieved for all of them?

R I filled out individual milestones.

I Okay, so what were – did you have any overriding concerns that point, when you were observing the resident?

R Overriding concerns in terms of, like, patient safety, or in terms of the resident's approach?

I Yeah, just about the approach or anything.

R There was nothing that I thought was immediately unsafe or needed to be modified. There were things that I might have done differently, and I certainly provided suggestions to the resident as we went through it together, but I didn't have any immediate concerns about how the resident did for their level.

I Okay, thank you, and what information or evidence did you use in making the decision to give this score?

R So, let me just – just give me two seconds just to reference back to that (pause) Okay, right, so the main items that I thought about were sort of the overall safety, so if the resident had a safe approach and was doing all the things necessary, once of which was to have myself or a staff – in this case it was myself – present, just because of the acuity of the patient, to ensure that, you know, it was a supervised encounter because of the acuity of the situation, and that was all very well done, and then taking a brief history. Definitely, for us, in that situation, it's a balance between being comprehensive and being timely enough to be able to make the decision.

I Right.

R There are certain things like any acute medications or doses that the patient needed to receive, and I confirmed and sort of assisted the resident with confirming those things, and I believe, in this case, we did have to add one additional medication that the patient hadn't received en route, and that wasn't something that the resident knew, but, at that level of training, I didn't expect that they would necessarily know that, and that was one of the milestones I had sort of put as 'in progress'. There was some clarification with me as to just making sure we had all the appropriate treatments on board, so I found it helpful, in that sense, to be able to sorta document that with the individual milestone..

I Okay, thank you, and would you say that the resident's performance earlier on had any impact on your score – like, in other encounters?

R I think that my previous encounters with this resident gave me enough confidence to be able to let her lead that discussion with the patient. This is a fairly junior resident, although towards the end of the year, was someone that I didn't know as well. Just based on the patient acuity, I likely would have had them just observe me perform that same encounter for the first time. In this case, I'd worked with this individual, previously, and I was comfortable that, with some guidance, she would be able to do that, so my willingness to let her take charge of the situation was based on previous encounters. As far as the particular scoring on this one, though, I tried to sort of assess each of these individually, based on that encounter.

I Okay, perfect, thank you. What would you say that your specific goals were when filling out this form?

R So, I've done a bit of work in the past years on assessment committees with competency based medical education and entrustable professional activities, and one of my goals – I think it was my goal for this resident – was to be able to capture a clinical scenario, which, I thought, for her level of training, she did quite well, and I wanted just to be able to have that documented for her, so that, going forward, hopefully, and review with sort of CBME principles, that this is something that she would be trusted to do with semi-independence, or at least indirect supervision, going forward, just to be able to document a skill set that she has.

I Okay, wonderful, and how would you say that this form helped you with these goals?

R I think that it gave a very quick easy way to be able to document this. There's no paper, there's no complicated log in process. I received an email that I got on my mobile phone, and I filled out the form in my phone, and that entire process took – for the individual EPA – less than three minutes, so it was very quick, very easy to be able to document this for the resident, and with the particular breakdown with the milestones, it let me highlight the particular areas where, you know, I recommended to her to spend more focus, which is, in this case, it would be the depth of knowledge and medical expert side. I thought her communication skills were very appropriate, etc., and that I was able to document with the tool.

I Hello?

R Yes.

I Oh sorry, I thought we cut out for a second there.

R Okay, did you get the end of my response there/

I I think you said something about document – allowed you to document.

R	Yeah, and so it allowed me to document quickly, easily, and she had certain parts of the milestones that were done, you know, certainly well enough that I think would be fine for her to do them independently in the future. To do them with I think indirect supervision would still be appropriate. So, the form with the individual milestones allowed me to break that down quite nicely, and with just the buttons to press to identify those responses, it's really very, very quick to use.

I	Okay, great, that's very good information. So, one of my final questions for you is how well do you think that the milestones help you communicate how the resident is progressing in the program, and what they need to do to progress?

R	I think that they're quite effective at that, particularly because, with how it is set out, we can just sort of press the buttons. I think that you're less likely to get that detail of feedback about each of the residents and their particular progressions. If it was – it's solely reliant comments, I would do my best to include comments, but, you know, from a time perspective, I think it's simpler to document what the resident is doing, and it really isn't an overwhelming number of selections. By having it broken down like that, if an individual isn't ready for complete independence with a particular task, and just has one or two areas to focus on, that can be highlighted quite nicely with that tool, so I quite like that format.

I	Okay, so kind of like you said, with patient care, it's kind of the mix between being thorough and also being timely.

R	Yeah, and, you know, particularly, in my discipline, that was just one of the keys, and I was able to sort of document that, plus there were sort of treatment decisions, written communication, discussions, you know, in terms of consent, or whether we would or wouldn't take the patient for a procedure. So, most of those realms I was able to encompass with that too.

I	Okay, wonderful, and are there any ways in which the form, you think, interferes with some of this, or something that maybe you don't particularly like about the form?

R	No, I don't think it interferes at all because, if the resident is expecting – or progressing very well, if they're ready to independently perform that EPA, there's the option to bypass the milestones with the highest score sort of on the first question, so if someone's doing very well, it isn't a hindrance of going and selecting, you know, seven or eight normal yes boxes. There's the ability just to bypass that from the start if they've done well, so I think that's actually a very nice feature.

I	Okay, wonderful. My last question for you, how do you feel that filling out this form, overall, helped or hindered teaching and patient care? The two components there.

R Yeah, I don't think that it had any effect on the patient care. I remembered the encounter well enough. I filled this out two days later, after the encounter had been done, after the resident sent it to me. I mentioned to her to send me one because of the encounter, in particular. So, in terms of effect on patient care, it didn't delay or harm or adversely affect the patient care in any way. In terms of teaching, I'd actually already sat down with the resident to talk about the particular situation, and that was sort of where the conversation to send this form came from. So, in this particular encounter, it didn't also encourage sort of a discussion around a teachable moment, but in other instances it has, but in this one in particular, I had already taken a moment to speak with the resident and said, you know, 'I know you have these forms. By the way, send me one', so really was, in terms of patient care, certainly not a hindrance, it was neutral, and in this particular instance, also probably didn't bolster teaching in a significant way.

I Okay, do you find that there are times when the forms are sort of ideally used?

R I think that the best time for the form is actually when – you know, in a more timely fashion, so within one or two days of having done the encounter. I find that, you know, I have the opportunity to interact with the residents on call, and sometimes when these circumstances come up on call, you know , it's a bit more challenging to take time – although not impossible – to take time to fill them out in the course of a busy day, but often it'll be the evening after when I'm post-call. The encounter is still fresh in my mind. I find that's a fairly – definitely easy time to get them down.

I Okay, great. Is there anything else that you'd like to add about this particular EPA or EPA's, in general, that we haven't discussed yet?

R No, I don't think so.

I Okay, great. So, I have your verbal consent. If you could just get me your signed consent forms within the next day or two, that would be wonderful.

R Okay.

I And, like I said, once the study's complete, then we'll be sending out gift cards, so just keep an eye out for that.

R Great, thank you so much.

I Thank you so much for taking the time to talk to me today. This was very, very helpful.

R Of course.

I Okay, have a good day.

R Thanks.

I Bye

R Bye.

END 14:09

T

CD1.81.P
10:52

I This is interview code CD1.81.P. Okay, so what I'd like you to do is just, thinking back or looking at the form if you have it available, just try to relive or visualize the moment that you were filling out this particular EPA.

R Okay.

I Just sorta put yourself in that headspace, I suppose.

R Okay.

I So I'd like you to describe if this encounter was more about direct observation of skills or about reviewing approach to diagnosis and management?

R Well, there was….

I Sorry? Sorry, you're cutting out a little bit. Sorry, I'm just wondering if this particular encounter was more about direct observation of skills or about reviewing approach to diagnosis and management? Okay, I can't hear you. Oh no, the call has ended.
So I was just asking if this encounter is more about direct observation of skills or about reviewing approach to diagnosis and management?

R I think it's more like the second – approach to diagnosis and management, yeah.

I Okay, perfect, and what were you noticing when you were reviewing with the resident that led to your score?

R I think he was, overall, pretty organized and systematic, and I think he had a pretty thoughtful approach. Yeah, no, I think he, more or less, kind of considered a fairly broad differential, and kind of next steps, immediate, but also the therapy, and how that kind of those work in tandem, kind of simultaneously for patients who are critically ill.

I Okay, excellent, and what information did you use in making the decision to give this score?

R Well, I mean, I guess mostly it was his reporting was organized, systematic, but also, I think, in the context of the patient, it just made, I think, logical, intuitive sense, what he was – the kind of story he was giving me, and also, considering what I heard before compared to the report from the emergency physician, it kind of made sense, but it also shed additional light on the patient, so –

I Okay, did you think that the resident's performance earlier on had any impact on your score? Had you worked with this resident before?

R Yeah, I worked with him before, but I haven't – like, this is – I was on call with him. I haven't had the opportunity to see him do – like, this was the first time I saw him do, like, a new consult, so I think it's kind of important 'cause, obviously, if I'm working with someone who is in the ICU, and we have these progress notes which tend to document things quite rigorously 'cause they're typed progress notes, so I think our residents can easily just copy and paste, as much as we try to actually dissuade that. But anyways, so when you're on call, the residents actually have a chance to go see a patient, give us a story that's new, and kinda get to see a fresh look at what they actually know.

I Okay, thank you. When you were filling out this form, what would you say that your specific goals were? What were you hoping to accomplish?

R I think just – well, my perspective is always, you know, it's accurate and provides some measure of insight, so I think that's hat I was hoping was that it did reflect kind of an actual event, and didn't just kind of gloss over, like, on a global scale. It just kind of identified each issues that were important for assessing him.

I Right, so you tried to make it more specific to this particular resident and this particular encounter?

R Yeah, I think so, yeah.

I Okay, thank you, and how do you find that this form helped you with these goals?

R I think the form, I actually kind of enjoy the way it's outlined. Like, the milestones, I think, kind of make more sense, rather than being like an overall likert scale from, you know, not sufficient to sufficient, or satisfactory or excellent, whatever. This just goes over, basically, kind of dependent to independent in terms of, like, in progress to achieved, and I think it's probably more realistic in terms of my understanding of assessment.

I Okay, wonderful, and are there any ways that this form interferes with these goals, or anything that you don't like about the form?

R No, I think it's very appropriate. Yeah, no, I think it's good, yeah.

I Okay, and so you didn't use the -- or fill out the milestones, I guess, especially for this EPA, but how do you think the presence of these new milestones impacted your assessment - just having the option?

R Oh, I thought I – oh, I filled out the part where it says, like, 'in progress to achieved', I thought.

I Yeah.

R Yeah, yeah, but you mean the individual, like—

I Yeah, so sometimes , I guess, when it's not achieved, you can sort of fill out the…

R Yes, that's right, of course Okay, yeah, I know what you mean, sorry, yeah.

I So just having the option t do it that way, how do you think that that impacted your assessment?

R Yeah, I think it's reasonable. Like, again, I think it's very practical or very pragmatic in terms of the assessment. Like, if you do believe that the resident did a reasonable job and did effectively what you would do in a given situation,I think it merits that they're – they have an independence in one area, so no, I think it's a fairly flexible and realistic form, especially if the resident is performing exceptionally well, then I think it's very reasonable. I think it would become more difficult for each milestone. If the resident was very new and maybe had multiple areas where they were demonstrating signs of gaps in knowledge or skill, it might become -- like, if each milestone does have subheadings, if you're in progress, then it may become very difficult and lengthy to complete.

I Right, okay, and lastly, how do you feel that feel that filling out this form, overall, helped or hindered teaching and patient care?

R I don't think it actually hindered. I think it, again, provides a very kind of realistic and on time kind of feedback and evaluation, although I guess it's not really feedback because, technically, I don't think – I'm not sure of this – but I don't think the resident would receive this form. Is that co -- ?

I No, they do receive the forms, yeah.

R Oh they do?

I Yeah.

R Okay, and at that time or at a later date?

I I believe the way that it works is once you submit it, they're able to view it.

R Oh, interesting.

I I think that's how it works.

R Yeah, well, I guess, you know, in that sense, then I think it's very reasonable because I know, just from my own perspective and from other perspectives, I think residents always want to be seen as being excellent or outstanding or

above average. I think most residents kind of lament the idea of being average or being below average, so I think this is – it fully allows you to be a little more objective, but also not, like, you know, tell the resident that they have particular weakness; just that there are areas in which they need to improve, which I think is appropriate.

I Right.

R So, I think, in that sense, it probably is quite helpful, I think, for the resident ‘cause it is a little more pointed at the time of the feedback ‘cause I think most residents – and speaking from my own perspective – generally, when you get feedback at the end of the rotation, it’s kind of global and it’s – there’s little kind of individual points of reflection. It’s just kind of glosses over everything with kind of general statements, so I think this probably gives much more pointed and probably objective feedback to the resident on areas they can improve.

I Okay, thank you, and would you say that there’s any way that this form helps or hinders patient care?

R No, I think it’s easy to perform. I mean, I think, if this form would happen for every single resident on every single consult, it might become a nuisance, I would say, but I think – I mean, I’ve – like, as far as I’ve seen – I’ve only used this form, I dunno, three or four times – and it’s been fairly limited, but I would think, if this became, like, every single consult, it might get a little bit annoying.

I Right, in moderation.

R Yeah, it’s kinda in moderation, yeah ‘cause we’ve got, obviously, work to do and people to see, so, if you’re filling out evaluations constantly, it could become very, very cumbersome.

I Yeah, and I could see how, if you’re filling them out constantly, it might be, yeah, like you said, cumbersome to have to fill out those milestones each time, and you might be sort of tempted to be like, well, it’s achieved (laughs).

R Yeah, exactly, yeah, exactly, so I think, you know, like you say, in moderation is reasonable, but if it’s every single procedure, every single patient, this would get really old, really quickly.

I Okay, perfect, thank you for that.

R Yeah.

I I don’t have any other specific questions, but is there anything you’d like to add about this particular EPA or EPA’s in general?

R No, no, I think they're pretty reasonable, yeah.

I Okay.

R I guess I do have a degree of bias because I'm doing a masters in medical education, but I do think that they do have, I think, more merit just 'cause of the kind of individualistic and discrete event is probably more – just offers up more kind of direct feedback, rather than saying, you know, 'overall, did you did well in this rotation'. It's just a little more pointed.

I Right, it gives the resident a little bit more of concrete sort of specific feedback fo them to sort of digest and –

R Exactly, yeah, for sure.

I Okay, wonderful. So like I said, once the study is complete, we're gonna be sending out the gift cards, so keep an eye out for that.

R Sounds good.

I But other than that, I have no more specific questions, so thank you very much for speaking with me today. This is very helpful feedback.

R Yeah, no problem.

I Okay, you have a great day.

R Okay, thank you.

I Bye

R Bye bye.

END 10:52

FD1.53.P
10:29

I Alright, this interview code FD1.53.P. Okay, so what I'd like you do, just to get started, is to think back, and try to relive or visualize the moment that you were filling out this particular EPA.

R Okay.

I Just sort of put yourself back in that position, and I'd like you to describe to me if this encounter was more about direct observation of skills or about reviewing approach to diagnosis and management?

R It would be the latter, yeah, approach to management, yeah, yeah.

I Okay, and what were you noticing when you were reviewing with the resident that led to your score?

R I guess I was looking for a few different things – whether it was comprehensive, whether she picked up on the pertinent, and as a consequence, sort of realized the sort of severity of the case, and how that informed her management plant.

I Okay, perfect, and what was some of the information or evidence that you used in making the decision to give this score?

R You know, in terms of evidence, it was really me, and, I guess, looking at, you know, some of the questions that the EPA asks of me, you know, assessing, diagnosing and initial management of the patients. I guess I'm literally just looking for those features to be a) one thought of, and b) was it appropriate for her level of training?

I Okay, thank you. If you – I dunno if you've worked with this resident previously, but if so, did their performance earlier on have any impact on your score?

R I would say so, perhaps indirectly. Well, actually, not directly, certainly. It certainly did inform my expectation of the resident, but I don't – I think I was able to judge this particular interaction, uniquely, sort of separate from her other sort of presentations and that, yeah.

I Okay, and when you were filling out this form, what were some of your – or what was your specific goal? What were you hoping to accomplish?

R I guess a couple of things. One would be actually providing useful feedback for the resident. The other aspect would be simply to help her achieve her EPA's, as well. I know they have a few of those to target.

I Okay, perfect, and how would you say that the form helped you with these goals?

R You know, I think the – where I was able to offer the most useful feedback was probably in the narrative. So I'm just looking at the form here. You know, points number 8 and 9, just where I'm asked to comment on the good and bad aspects of the performance, I think that's where I was able to provide, sort of, tailored, individualized information for this resident.

I Okay, perfect. Are there any ways that you think the form interferes with these goals?

R Not really. You know, the section 7, the milestones, some of them – many of them didn't apply, necessarily, to this case, so I think it was a bit of a distraction in terms of not having the option to choose 'not applicable' or 'not observed' point, yeah.

I So I see that you left a couple of them blank, so that's indicating that they weren't applicable to that case?

R Yeah, that's correct, yeah, yeah, yeah.

I Okay, so they sort of be distracting, or maybe not all cases are gonna fit within those milestones?

R Correct, yeah, yeah, I think distracting and, yeah, sometimes you just lose signal to noise if there's just too many extraneous pieces of data, I think, yeah.

I Right, okay. This is just sort of my own question just 'cause I'm curious, but do you find that the questions, because they're quite specific, do you find that they limit, sort of, what your actually looking for in terms of competence?

R Hmm, are you referring to all of the 9 or 10 points here?

I Yeah.

R Yeah, um –

I Like, are there things that you would otherwise look for that maybe aren't included?

R Yeah, included in here, yeah.

I Um hmm.

R That's a good question. Not off the top of my head, and even if there were, I think sections 8 and 9 are – they give me that flexibility to introduce those areas that I would like to comment on.

I Okay, good, thank you.

R Yeah,

I So you did use the milestones in this particular case. How do you – how well do you think they help you communicate how the resident is progressing in the program and what they need to do to progress?

R Yeah, that's a good – that's a tough question, I guess. Let's see, I would say they're fairly valuable. They do help you hone in, in an itemized matter. There's, like, facets of, in this case, the consultation and assessment that the resident did. I think they're helpful. I think there's a little bit of, perhaps just in my reading of it, some redundancy perhaps. The first line is 'recognize urgent problems that may need involvement of more experienced colleagues, and ask for assistance', and then, I think, a few down there's 'seek assistance as needed'. You know, there are subtle differences in those two points, but I found those to be somewhat similar.

I Okay.

R But yeah, I think it's fairly comprehensive in terms of the different facets of what I would expect in a consult from that resident.

I Okay, thank you.

R Yeah.

I Just one last question for you. How do you feel filling out this form, overall, helped or hindered teaching and patient care?

R Hmm, with respect to teaching, I think it offers a formal medium of providing feedback, which I think is useful, you know, for the resident in particular, to reflect on in the future if they ever want to or need to.

I Um hmm.

R It also helps me, I think, formally document concerns that I have with the resident. I didn't have any major concerns in this situation, but if I did, it's a formal way of me documenting that, yeah.

I Okay, and do you think that there is anything you could comment on with respect to patient care?

R You know, I'm not – I don't see a direct correlation with respect to improving patient care, other than the fact that in, you know, providing feedback and teaching a resident, you know, you're helping them grow as a physician, and

hopefully, down the road, provide better patient care, but I don't see a direct correlation, for example, this EPA and the care that that patient had or was going to go on to have.

I Okay, makes sense, thank you.

R Yeah, yeah.

I Is there anything else that you would like to add that we haven't already spoken about?

R No, I think that's – yeah, I think the EPA format is fairly easy to use, which, you know, it makes it a little bit easier to use and saves time, which is good. I would encourage maybe continuing to try to keep it fairly succinct, for two reasons. One, sorta the barrier to completing them, often times, is proportional to how intensive they are; and then, number two, sometimes, again, you lose signal to noise, perhaps, in terms of trying to communicate the essence of what you felt about a specific encounter, yeah.

I Okay, that's useful, thank you.

R Yeah.

I So, like I said, we'll be sending out the gift cards once the study is complete, so just keep an eye out for that.

R Sure.

I But other than that, I don't have any more questions for you, but thank you very much for taking the time out of your day to talk with me.

R No problem. My pleasure, [name].

I We really appreciate it.

R You're welcome. Yeah, take good care.

I You too, have a good day.

R Thanks.

I Bye

R Bye

END 10:29

FD1.33.P
11:44

I Alright, so this is interview code FD1.33.P. Okay, so my first question is, did you – just for context, did you fill out this form with the resident in person or did you use the ‘save for later’?

R She emailed it to me, after.

I Okay.

R Like a day after…we had the encounter.

I Alright.

R So I did not – yeah, so I filled it out without her.

I Okay, perfect, and why did the resident use this ‘save for later’ as opposed to doing it in person? Was it just sort of a convenience thing?

R I have no idea (laughs), really. I mean, she – ‘cause I filled out – I think I filled out four for her, and she had emailed them all to me, and I shouldn’t say I have no idea, but she did say that she feels like when she asks to do it at the moment, then it kind of interrupts the flow of the day.

I Right, that makes sense. Do you know why this resident chose this particular case? Would you have chosen this case to document?

R I think – I mean, again, I did about four for her, so I think this – yeah, this was a perfectly reasonable case to, I think, demonstrate some of her skills and that kinda thing, so yeah.

I Okay, would you say that – so you said that you filled this out later, so was it more about reviewing her approach to diagnosis and management, then, as opposed to a direct observation of skills?

R For this one, I would say the key kind of component that I was evaluating her on was, yeah, about kind of diagnosis and more about the, yeah, diagnosis and management as opposed to history taking or something like that.

I Okay, great. So I’d just like you to sort of put yourself back in the position when you were filling out this form or reviewing with the resident. What were you noticing that led you to your score?

R Well, I think she did an excellent job with, you know, collecting all the relevant information, and was able to really kind of differentiate between colonization and infection in this somewhat complicated history. So, that’s why I gave her a ‘can diagnose and manage this case with no significant change’.

I Okay, great.

R ‘Cause I didn’t change her management (laughs).

I Right, that makes sense, and would you say – so you filled out a few of these for her that day, do you think that her performance earlier on had any impact on your score – so, like, just your previous experience with her?

R I wouldn’t – I mean, certainly she was an excellent resident, but I think that, you know, each – yeah, you need to take each case, and, I mean, you need to look at it individually, and how she performed on each case, so I wouldn’t say that, necessarily, the other cases informed this case.

I Okay, and what would you say were your specific goals when filling out this form?

R My goals?

I Um hmm.

R I guess, I mean, just being able to highlight what she did well, and, I mean, again, there weren’t really a lot of areas for improvement. I thought that, overall, really, just, you know, I mean, I think the thing that she did the best was, again, kind of taking the whole history and the context and the exam and everything, and being able to identify a situation where there is colonization versus infection, and how that should be managed, and so really just highlighting the fact that she could that really quite independently.

I Okay, so moreso about giving her feedback, and communicating to her that she had done things appropriately?

R Yup.

I Okay, and how would you say that the form helped you with these goals?

R Well, I think, again, in this situation, because I chose the ‘can diagnose and manage this case with no significant changes’, then it’s, you know – I mean, I think it’s not quite as – like, all the milestones where – were skipped, right?

I Right.

R So, I think, again, it’s maybe – I mean, it maybe just helps to focus because you’re – you know, you need to write comments and everything, so just focussing your comments on what the resident did well or – was helpful, I guess.

I Okay, and so, yeah, you didn't fill out the milestones for this particular EPA, but how do you think the presence of these new milestones impacted your assessment – just sort of having the option?

R Yeah, I think – I mean, I think, in looking over the milestones, I think that if I had chosen, you know, one of the other options in number six, then it does kind of help to focus where you think they may need to – need some help or need to improve, so I think that that's helpful.

I Okay, and are there any ways that this form interfered with some of these goals to communicate feedback to the resident?

R I don't think so.

I No? Okay.

R And I think – I mean, the one thing, you know, I would say that, I mean, certainly I did giver her feedback in person on some of her cases. I didn't give her specific feedback in person on this case, so maybe it helps that, you know, getting this sent to me, she now has feedback, or she wouldn't have gotten it, so that's a positive.

I Right, okay. Yeah, that was the next question I was gonna ask, actually, was did you verbally give her any feedback that was documented on this form? But you answered that.

R Yeah, so, I mean, the short answer is no, not for this particular case. You know, it was one of those cases where she really did everything perfectly well, and I think I probably said that, but, you know, I don't think I gave her a lot more feedback than that, at the time.

I Okay, thank you. How do you feel that filling out this for, overall, helped or hindered teaching or patient care?

R I think, I mean, in terms of how does it affect patient care? I mean, hopefully, it will provide more feedback for the resident so that they can improve their patient care; but, specifically for this particular patient, I don't think it necessarily affected in a positive or negative way, because I was filling this out after the fact.

I Right, that makes sense, and with regards to teaching, how do you think that it was helpful or a hindrance?

R I think it's – no, I think these are good. I mean, as much as I'm sure some staff don't like multiple ones, I think it does make you focus your feedback on – you know, by narrowing it down to a particular case, I think that the residents probably get better feedback in terms of something that's a bit more concrete.

So, I think overall, it's probably helpful in terms of – you know, as opposed to some kind of global gestalt that they often get. This is more specific.

I Okay, thank you, and lastly, what are your thoughts on the 'save for later' option?

R Well, I actually think, in some ways, it might have been – I mean, again, she sent me a whole bunch after the fact, and I don't actually think it would have necessarily impacted workflow in most of the situations, and so I would have been happy to fill them out at the time because, you know, everything's kind of more fresh in your head (laughs) when they just presented, and they probably would get better quality feedback, but I do – I can appreciate that maybe the residents don't – like, you know, feel, I guess, unsure about interrupting the workflow to have these done, or there may be situations where it's just too busy or something like that, so I do think it's probably good to have the option, but I would say that, as much as possible, the residents should be encouraged to get it at the time because I think they will get better feedback 'cause it's – as I said, it's kind of more in the moment, and I think, you know, there's certainly going to be – and I've gotten this feedback from other residents – that there are situations where may send it to their preceptor, but then their preceptor doesn't fill out it, and that is always the – you know, the downside, I guess, of asking them to do it later.

I Right, and I guess there's a technological sort of aspect to filling these out, too, so I imagine certain preceptors would have an easier time doing them than others, or be more willing to do them in the moment than others.

R Right, so, I mean, I do think it's good to have the option in those situations where you really – the resident really feels like they can't ask in the moment, but I think, overall, residents should be encouraged to get them done at the time.

I Okay, so what do you think would happen if this option was removed to force in person feedback?

R I think you probably would get less.

I Less EPA's completed?

R Yeah, I think so.

I Okay, perfect.

R I mean, I think, mostly because the residents may not ask, so yeah.

I Okay, excellent. Okay, so that's all of the specific questions I have for you, but is there anything else about this EPA or about EPAs, in general, that you'd like to add that we haven't talked about?

R I don't think so. I think, overall, you know, it's a pretty easy form to fill out. It's pretty short; it doesn't take that long, so I don't think there's any major issues.

I Okay, great. Well thank you so much for chatting with me today and taking the time out of your lovely, beautiful sunny day to do so.

R No problem.

I It's very helpful. So once the study is complete, we'll be giving out those gift cards, so just keep an eye out for that.

R Okay.

I Okay, thank you so much.

R Thanks a lot, okay, bye by.

I Have a good day, bye.

END 11:44

FD1.33.R
08:36

I Alright, so this is interview code FD1. 33.R. Alright, so what I'd like you to do is, just thinking back or looking at the form if you have it in front of you, try to relive or visualize the moment that your preceptor was filling in this form.

R Okay, so there was actually a discrepancy between that because this is actually a form that we discussed and then she ended up filling in later.

I Okay, so just kinda put yourself back in that position, I suppose.

R Um hmm.

I Just in that mental space, and also before I forget, can I just get your verbal consent to participate?

R Yes.

I Okay, perfect, can't forget that. Okay, so what I'd like you to do is just describe to me, first of all, if this encounter was more about direct observation of your skills or about reviewing your approach to diagnosis and management.

R It was more a review of my approach.

I Okay, review of your approach, okay, perfect, and what was some of the information that you think your preceptor used in making the decision to give you this score?

R So, I saw this patient on my own, and then we reviewed it together. She then went into the room with me and confirmed some of the information as well as the physical exam findings, and then based on the plan that I had told her, she made minor adjustments then said, 'This is what we're going to continue with', so she didn't directly observe me doing the consult, but she confirmed everything that I had done up until that point.

I Okay, perfect, so then what do you think was some of the evidence or information that your preceptor would have used in making the decision to give you that score?

R Sorry, what is some of the evidence that she would have used to – sorry?

I In making the decision to give you that score, what were some of the information that she would have used?

R Okay, so it would be my – well, some of the things that she focused when she was doing the evaluation for me was to talk about the way I presented the case and the information I chose to present, and the present, and the pertinent

positive and negative – she told me that I was able to catch those appropriately – and then she confirmed all the details with the patient, and then subsequently went on to say that she agreed with the plan that I had proposed with this patient, and then saying that is also what she would have done, so I believe that's what she used.

I Okay, perfect, thank you. This only applies, I guess, if you've worked with this preceptor, previously, but do you think that they would have used only this interaction, or other observations from that day?

R I'm not really sure if I can comment on that. I've never worked with that preceptor before, and that was one of the first EPAs I filled out with her.

I Okay, what was your specific goal when getting this form filled out? What were you hoping to accomplish?

R For this specific one, it was my approach to a patient with respiratory distress, and really determining whether this was an infectious ideology or not, based on the microbiology, and so the attending team at that time said that this needed to be treated, and I guess the question for us was whether it actually needed to be treated or not, so my goal was to actually determine what the pertinent positive and negatives were in terms of treating versus not treating, and that's what I wanted her to assess at the end was whether the decision I made not to treat was actually an appropriate one, based on the information that I had gotten.

I Okay, wonderful, thank you, and what would you say that your preceptor's goal was when filling out this form?

R I think, for her, she felt that this was a good case in terms of sort of a learning moment, in that when we're interpreting microbiology results, we need to be a little bit more discerning about what we're seeing, and not treat everything that we see, so I think that's why she chose to do an EPA for this one.

I Okay, great. How would you say that this form helped you with you and your preceptor's goals?

R Sorry, could you repeat that again?

I Sure.

R I'm in the hospital. Everything keeps cutting out.

I No worries. How would you say that this form helped you with your and your preceptor's goals?

R I think, for me, she was able to tweak a little bit, in terms of some of the finer points, especially specifically to that particular case, in terms of interpreting the microbiology results in future cases that might come up like that, and, for her, I think it gave her a chance to actually sit and watch me do that, and see whether she would be able to trust my plan, going forward, in future consults.

I Okay, wonderful. Are there any ways in which this form interfered with your and your preceptor's goals? Maybe some things you didn't like?

R No, it did not, but I will say that, at the time that I asked her to fill out the EPA, she was very willing to do so, but it was – I found this a lot as well with other EPAs – that they tend to want to say 'just send it to me and I will fill it out at home' because it's faster for them. It also depends how tech savvy they are. If they're good at texting or putting that information into a form, they're more willing, but in this case she said she would prefer to do it at home, yeah.

I Okay, wonderful.

R So that was probably a little bit of a barrier for getting it done, face-to-face.

I Okay, so some barriers there would be things like the technological savviness of the particular preceptor, and maybe, like, the time or availability that they have in that moment to fill it out?

R Yeah, just how much is happening around us at that point in time, yeah.

I Okay, thank you, and how would you say that filling out this form, overall, helped or hindered teaching or patient care?

R I don't think it had any – I don't think it hindered patient care at all. I do think that there are times when asking for an EPA is a little bit more opportune than not, and I think, in this case, had this been earlier in the year, I know now when to pick my moments for an EPA versus not, but there is a little bit of a time crunch in trying to get them done, so I feel like that's been a little bit of a barrier – sort of working that around trying to get as many as I can, but not – ensure that I'm not interfering with the workflow of the day.

I Right, okay, and then could you also comment how filling out this form would have helped in terms of teaching?

R I think, from the perspective of the person that's evaluating me or -- ?

I Yeah, yeah.

R -- teaching or myself. I think, for them, especially before you go in to see the patient, I had asked her if she'd be willing to an EPA for me. Doing that, I find,

makes them actually watch you a little bit more and listen a little bit closer because now they're – put themselves into sort of an evaluator role, and so I find that I get better feedback when I tell them., before I see the consult, that I would like an EPA for it, because now they're actually wanting and evaluating you.

I Okay, that's really good information, actually, so it kind of primes them to be in that evaluator role instead of sort of just going about it how they normally would.

R Yeah, yeah, and then now they've automatically just set aside a few minutes at the end to actually to give you the feedback , instead of trying to say after you're done, 'Oh, can I get an EPA for it' when you've already discussed everything.

I Right, okay, that makes a lot of sense. Thank you.

R Okay.

I Those are the specific questions I have for you, but is there anything else you'd like to add about this particular EPA or EPAs , in general, that we haven't talked about?

R No, nothing at this point.

I Okay, perfect, so I will get that consent form from you either today or tomorrow. If tomorrow's more convenient, that's okay, I have your verbal consent.

R Okay.

I And just as a reminder, we'll send out the gift cards once the study is finished, so just keep an eye out for that.

R Okay, will. Okay, thank you so much.

I Okay, thank you so much for chatting with me today.

R No worries.

I Have a good day.

R Okay, bye.

I Bye.

END 08:36

FD1.19.P
14:18

I Alright, so this is interview FD1.19.P. Okay, so my first question for you is, did you fill this form out with the resident, in person, or did the resident use the 'save for later' function?

R Filled it out with her.

I Filled out with her, okay, perfect. Why do you think that the resident chose this case?

R I actually initiated -- suggestion that we should do an EPA because of the procedure that was done, so – or because of the situation, I said, she could do an EPA.

I Okay, so you saw it as a good situation or case to use as a learning – sort of learning experience for her?

R Yeah.

I Okay.

R Yeah.

I Would you say that this was more about direct observation of the resident's skills or about reviewing their approach to diagnosis and management?

R It was a bit of both because part of the interaction was independent, which I reviewed after, and then part of it was with direct observation, so I think it was – that particular situation was a combination.

I Okay, so a little bit of both. So, just try to visualize or relive that moment that you were observing and reviewing with the resident. What were you noticing that led to your score?

R What was I noticing during the interaction or -- ?

I Yeah, just during, I guess – the score that you gave this particular resident for the EPA, what was the some of the, sort of, information that you would have used in order to give them this score?

R It was the information that – I mean, it was her actions. So, contacting me with the information that a patient was ill; the information that she provided me on the phone,; hen when I arrived on the ward, the information that she already gathered with regards to management and the treatment that she had already initiated, and then some of the discussions we had with regards to further management. So it was primarily her actions throughout the interaction.

I Okay, great, thank you. Would you say that – this sort of depends on if you've worked with this particular resident before – but would you say that their performance earlier on had any impact on your score – or previous experience with this resident?

R Yeah, I think it would have some impact, sure.

I So, this was a resident that you'd worked with before that maybe had – you had particularly good experiences with, for example?

R Well I'd only worked with her on this week of CTU, so it's not like there was a – prior to this week I hadn't worked with her, but my interactions during the wee had been positive, and I think that does – that does influence the interaction to some degree – or the evaluation to some degree.

I Okay, thank you. What would you say that your specific goals were when you were filling out this form?

R My goals?

I Um hmm, what were you hoping to accomplish?

R First of all, to help the resident to complete some of the EPA's that they're required, and to provide some feedback on their performance and areas to improve on.

I Okay, great, and how would you say that the form helps with these goals?

R Definitely the fact that it's point of care, that it's easy to fill out and it's pretty straightforward. You know, I liked – because there was this sort of an automatic default if I felt that she did diagnose and manage with no significant change. The fact that the whole section seven was automatically filled out was also helpful.

I Right, so yeah, you didn't use the or fill out the milestones for this particular EPA because – or you clicked that they were achieved.

R Yes.

I But how would you say that the presence of these new milestones impacted your assessment?

R Well I read through them all, and just made sure that what I had checked off in six was still appropriate, just to make sure that I was capturing all of the tasks or components required by this EPA.

I Okay, great, and would you say that there's any way that this form interferes with the goals that you have?

R I don't think so. I do wonder whether – again, although I liked the fact that I didn't have to go through all of the milestones, per se, I did read them, and so if I was doing a lot more of these, that function would be nice, but if I had never filled out this EPA, I almost wonder whether the staff person or the more senior evaluator is required to go through the EPA's – not the EPA's; the milestones – to clarify, but once you've done the same EPA a few times, then I like the way it's currently set up, so I don't know if that's a possibility, even, ha.

I Um hmm, sorry, can you just sort of maybe rephrase what you mean by 'possibility'?

R Possibility? Well, if the form could be tweaked to (00:06:030 who the preceptor is or who the evaluator is.

I Sorry, your phone's kinda cutting out a little bit. You kinda sound like a robot.

R Oh, I'm sorry. If there was a way for the system to recognize that the evaluator has never filled out this EPA before, and if that was the case, it would lead the evaluator through all of the other (00:06:35). Whereas, if the evaluator has completed this form multiple times, then (00:06:42) has achieved everything (00:06:47) question number six, that those are automatically filled. If the system could allow that (sound was robotic throughout)

I Yeah, that's good feedback, actually. I sort of missed – for some reason your phone is kind of giving you a robotic effect (laughs), so –

R Oh, I'm sorry.

I It's okay, I'm only kind of getting every other word. I think I got most of it, but then it sort of cut out near the end there. So, you were saying, if the system could recognize whether the preceptor had filled out an EPA before, and then if they hadn't , to sort of guide them through it. Is that what you were saying?

R Yeah, and not any EPA but that particular EPA (00:07:36) EPA's are a little bit different and they have their own sort of nuanced requirements. (robotic)

I Okay, alright. Yeah, I find sometimes with the interviews, if the individual's in the hospital, sometimes the service cuts in and out or kind of reduces the audio quality a little bit.

R Yeah, I'm definitely in the hospital.

I Definitely in the hospital, okay, so we're still kind of getting that robotic effect. I only have a few more questions for you, but I might have to sort of – if I don't pick it up, I might just have to ask you to repeat it.

R Okay.

I Okay, so did you verbally give the resident any feedback that was documented on this form?

R I went through the form with the resident, and talked on each point. I don't (00:08:36) anything dramatically different than what is captured in the form, but, you know, we did talk about the case and the evaluation.

I Okay, so you did go through it with them, but there wasn't anything dramatically different?

R No.

I Okay, great. How do you feel that filling out this form, overall, helped or hindered teaching or patient care?

R I think it helps teaching merely because there's specific, direct feedback (00:09:19) that they are receiving feedback. Whereas, (00:09:26) without a form or something more formal, trainees don't necessarily consider it true feedback, unfortunately, but the EPAs really put it in that context, so I think it definitely helps with teaching (00:09:45) find it kind of neutral with regards to patient care, other than it's relatively quick to do.

I Okay, is there maybe another spot in your office that would have a little bit bitter reception?

R No.

I Like, the left corner or something.

R Is there anywhere I can call you at because there is a (00:10:05) line here, but it doesn't receive outside calls but (00:10:06). (robotic)

I Sure, yeah, you can give me a call back. My number is 204 – 294 – 9998. I just have maybe one or two more questions for you.

R I'll (00:10:28) Right away.

I Okay, thanks so much.
(pause)

I Okay, we are rolling. Sorry, do you might just sort of repeating your previous answer? So, I asked you how you feel that filling out this form, overall, helped teaching or patient care.

R So, I think it's relatively neutral with regards to patient care, other than it doesn't really impede patient care 'cause they're relatively quick to fill out. I do think it enhances teaching because the trainee clearly knows that they're receiving feedback – it's quite intentional – rather than the traditional method where we provide feedback, and often the trainee doesn't really feel that they're receiving feedback.

I Right.

R So it's quite intentional, and it helps the evaluator also know what the program is, in fact, requiring of the trainee.

I Okay, great. Thank you, that's really helpful. My last question for you is, what are your thoughts on the 'save for later' option -- so, the option for the resident to sort of send you the EPA later for you to fill out?

R You know, I think they're reasonable. I would – you know, I think there should be, potentially, a limit on how frequently that can be used 'cause there's definite value in filling the forms out, directly, with trainee.

I Um hmm.

R But that being said, I have had experiences where the timing doesn't work, and, you know, it was nice to still provide feedback even though it's a bit later, so, you know, it would be reasonable to keep, but there perhaps needs to be some limits on how frequently it can be used by each trainee.

I Right, so how would you feel if it was removed to force in person feedback?

R Sorry, if it was removed completely?

I Yeah, to force in person feedback.

R I still can't hear you.

I Oh, sorry, so, if this 'save for later' option was removed, which sort of forced in person feedback.

R Yeah, I mean, that type of feedback is the ideal. I have to say that there are situations, particularly in the emergent situation, or particularly the on call when you're not really with the resident on the team regularly, where it might hinder their ability to get an EPA, but, without a doubt, the in person is the ideal.

I Okay, so do you think that, if this option was removed to force that in person feedback, that you'd just get fewer EPAs in those kind of situations?

R You might get a small reduction in the number of EPAs

I Okay. Alright, thank you very much. So, like I said, once the study's complete, we'll be sending out the gift cards, so just keep an eye out for that. I believe that we'll be sending them out by email once we're done the study.

R Okay.

I So yeah, thank you so much for taking the time out of your day to chat with me. This is very helpful information.

R Okay.

I Okay, have a good day.

R Have a good day.

I You too, bye.

R Bye

END 14:18

I Okay, this is interview code C1.43.P. Okay, so my first question, just sort of for context, when you were filling out this EPA, did you fill it out with the resident, in person, or did the resident use the 'save for later' option?

R The resident used the 'save for later' option. We didn't fill it out together.

I Okay, great. Do you know why the resident chose to use the 'save for later' option as opposed to doing it in person?

R We didn't discuss it, but I think, really, a convenience thing. We were in a busy service, and we did the case, discussed it, went through – I went through his presentation and his management, but for convenience, with having to go on and see other people, the actual filling out was not (00:00:58).

I Okay, great. Why do you think that the resident chose to fill out an EPA for this particular case?

R I think it was a good case that he solely – well, (00:01:20) solely managed – but that he did by himself from the point of receiving the referral to seeing the patient, going all, sort of, through, constructing a plan, and then presenting it, and doing it – taking the history and examining in front of me, and then discussing it afterwards with me. It was a full case, and it was a good one in terms of the complexities involved in it, and in terms of the likely differential and management plan.

I Okay, great. Was this more – this encounter more about direct observation of the resident's skills or about reviewing their approach to diagnosis and management?

R So, I'd say it involved both, but with the discussion and then the exercises and milestones themselves, was probably more a discussion, you know, going on from the differential diagnosis and management.

I Okay, perfect, thank you. Just take a second to sort visualize and relive that moment that you were reviewing with the resident or filling out this form. What were you noticing, or what was some of the, I suppose, observational evidence that led to your score on this EPA?

R I think, so if we're looking at section B and the milestones breakdown, I think the 'generation and prioritization of the differential diagnosis' was very important, and around which a lot of our discussion revolved. Again, that sort of links, and also with the – you know, the appropriate investigations to be selected, based on the differential. We didn't have all the results available to interpret them, and I think, also, the final milestone box was important where it affects other healthcare professionals, including other physicians. This was a patient in the emerg who'd

initially been referred to another service, and we were a consult service, and then there had to be a lot of discussions as to who was going to take formal lead in the patient's ongoing medical care, and that involved discussion and negotiation, and that was probably an important and unanticipated aspect of the case. So, I guess those would be the ones that would jump out at me, in terms of (00:04:14).

I Great, great answer, thank you. Would you say that the resident's performance earlier on had any impact on your score?

R So, his performance, having been on service with me for the week or the time before we get the EPA – is that what you mean by that question?

I Yeah, yeah, just any previous experience that you had.

R As in how influenced was I from having worked with him, as opposed to being able to take this as a case in isolation --- is that what you mean?

I Yes, yes, that's what I mean.

R Yeah, okay, so I think I – being completely honest, I think that, you know, unless you're in an examination setting, I think it's very difficult to now be influenced by how the resident has worked and has been with you on cases. I think it's difficult. I find it difficult to do it in complete isolation.

I Um hmm, okay, thank you.

R What were your specific goals when you were filling out this form? What were you hoping to accomplish or communicate?

I So, in terms of what I wanted the resident to get out of this form was I wanted him to be encouraged in his progress and in how he was handling things. I think, all too often, it's perhaps a construct criticism that gets taken away from any evaluation or discussion, and I think this form provided a good opportunity to highlight the positives for the resident to take away, but it also highlighted, I guess – and this resident is a very good resident – and so it was sort of picking holes, or it's – you know, it's not – the obvious negatives aren't there to – or not – but the obvious, kind of, options. These areas that they need to work on aren't always that obvious, but I think this form, sort of, allowed more discussion, particularly this generation, in prioritizing differential diagnosis and selecting appropriate investigation, that it gave an area for the residents to really think about, and I hoped that that would be a message with him having seen written down, that would make sense to him from the sort of teaching and feedback that we'd one ad hoc, and on (00:06:53) with other cases where I would say, 'Well, don't just order everything that's in the guideline or in the text, but you've got to think why you're doing this. How are you going to act on it, rather just ordering a (00:07:06) test because it might support a diagnosis rather than being the actual

one that you need', and so I was hoping that the form and actually having that in writing would make it to clear to him, the sort of major, kind of, ongoing learning outcome, but really it was an opportunity to give them good feedback, that they can positive away rather than just always focussing on what they perceive as the criticism or the area they need to work on.

I Okay wonderful. I think that gave a pretty good overview of how the form sort of helps you meet some of the goals. Is there any way that the form interferes with some of your goals?

R No.

I No? You like it?

R I like it, yeah, it's a good length, and it gives, you know, the options for, you know, their ability, you know, and number six is good, and the two comment boxes are – yeah, I like the form.

I Okay, great. Were you able to verbally give the resident any feedback that was documented on this form?

R So, I did not do that in terms of specifically filly in this EPA, but I did give them rotation feedback, a one-to-one evaluation and feedback, which included some of the things that I was able to put on paper in the EPA, but I didn't do a separate, specific feedback for this EPA.

I Okay, thank you. So, in this case, you didn't use the milestones because you kind of – you clicked the 'all' or 'achieved' , correct?

R Yeah.

I Okay, so how would you say that, although you didn't use them in this case, how would you say that the presence of these new milestones impacted your assessment?

R I guess it helped me to sort of focus how – you know, what to look for when assessing a resident – you know, things that are important rather than just the overall global impression that you get. Like, it actually helps things be – it's a more targeted approach, which probably makes it a much more robust evaluation in terms of actually, rather than thinking, oh, that resident's really good, it's actually, well, they are good at x, y and z, but maybe need to do a bit more on a, b, or c.

I Right, so a lot more specific and targeted sort of feedback points?

R Yeah, it makes you think in a more targeted way rather than just a general, oh they're good; oh, they're not so – they're not quite up to scratch or whatever.

I Okay, perfect, thank you. How do you feel that filling out this form, overall, helped or hindered teaching and/or patient care?

R So, it didn't hinder patient care, and it was completed after, as I said at the start, and in terms of – sorry, what were other two things you said?

I In terms of teaching.

R Teaching?

I Yeah.

R Well, I think – so I'll have to say I'm not sure of what the rules are for how many the residents are supposed to do or when. I think maybe doing one at start and end of a rotation with the same physician would be helpful because there maybe things in the milestones that are – that require some change to approach may then be (00:11:12) milestone, have achieved it by the end of it. I think that would potentially be helpful.

I Like, a little bit more progress monitoring, almost?

R Yeah, I mean, it's – I mean, (00:11:27) services run a different way, but our service runs for a week, and by the time you take out a post call day or a stat day or whatever, your time with the residents can be limited, and so, I guess, although it is a snapshot, you know, you do want the feedback or the completing the loop, or, you know, the ongoing – the learning as a ongoing process, in order to give useful feedback or feel that they've addressed or the like, or give them an opportunity to hit that achieved, rather than leaving it as a basic approach, for example.

I Okay, that's very good feedback, thank you so much. What are your thoughts on the 'save for later' option, and how would you feel if it was removed to force in person feedback?

R So, I wouldn't – I would be – I prefer the 'save for later' option. I prefer to be able to – you know, I feel that there is a discussion and that there is on the spot feedback with the case, and I would prefer to have a little bit of time and peace to properly complete a form, rather than, you know, hitting buttons to get it done to move on, as it were, which I think would be a potential trap in a busy clinical service that you could fall into. Obviously, if it was a case where there was a lot of negative feedback (00:13:04) is the case, then, you know, I think you're going to be doing that feedback, regardless, to the resident, and regardless of how difficult it is, and although sometimes forms can help with that because it makes

it easier to feedback, sometimes it can also hinder, and so, overall, I think I would prefer to have ‘save for later’. I think it allows a much more considered process, and then for me to sort of formulate things in terms of the areas for improvement, for example, I prefer ‘save for later’. I think there’s a risk that you could end up, you know, for ease of getting things done and moving on to the next clinical thing, you could end up ticking boxes and not actually sit and think about it, and the same could work in reverse. You could just go, okay, ‘achieved’, ‘achieved’, ‘achieved’, because that’s so much easier than sitting and going, ‘well, actually, I think we need to discuss’ or (00:14:02). I think, doing that all on the spot is too much. I think a separate, full feedback session from the end of the rotation that would take into account some of these things is preferable, in my opinion, and having ‘save for later’ for the actual forms is my preference.

I Right, so it allows you to sort of think about the experience more in depth, and then provide a more careful overview and feedback to the resident, as opposed to, potentially, not having time or energy in the moment to do that.

R Absolutely, yeah. You phrased that way better than I (00:14:41).

I I don’t know about that. That was a really wonderful interview. Thank you so much for all of your very insightful comments. Once we are done completing all of the interviews, we’re going to send out those gift cards, so just keep an eye out in your email for that.

R Okay, that’s wonderful.

I Yeah, but I don’t have any other specific questions for now, but if there’s anything else that you have to add that we haven’t talked about, please, please feel free to do that.

R Okay, great, yeah, and also feel free to contact me back again.

I Okay, thank you so much.

R Okay, great, thanks very much then, cheers.

I Have a nice day.

R Bye bye, you too, bye.

I Bye

END 15:27

CD1.43.R
11:21

I Alright, so this is interview code CD1.43.R. Okay, so the first question I have for you, just sort of for context, is did your supervisor fill this form out with you, in person, or did you use the 'save for later' option?

R I think this was a 'save for later'.

I This was a 'save for later'?

R Yeah.

I Okay, great. In this particular case, why did you chose to use the 'save for later'?

R I think 'cause everything was super busy. I'm just trying to remember. I think this was done near the end of the day, and so we decided just to do it later.

I Okay, makes sense, and why did you chose this particular case to do an EPA for?

R It was the only one I had done that day.

I Okay. What was – sorry – where am I here? Okay, was the encounter with this patient more about direct observation of your skills or more about reviewing your approach to diagnosis and management?

R It was more about approach.

I Reviewing your approach, okay.

R Yeah.

I And what was your specific goal when getting this form filled out?

R Documentation (laughs).

I Documentation?

R Yeah.

I Okay, so can you just tell me a bit more about that?

R Yeah, oh, I just needed EPA's, and so I was just trying to document them.

I Okay, great. Okay, and in terms of your preceptor, what do you think that their goal was when filling out this form?

R I think just, like, contributing to medical education, I guess. It needed to be done. I asked him to do it. I guess – I'm not really sure.

I So, on the one hand, sort of going alongside your goals – like you're trying to get your EPA's filled out, so contributing to that, but then also contributing to medical education in – in what sense?

R Well, I think they're a good feedback mechanism, yeah, and I think we – moving to (00:02:45) based medicine, we need to be doing lots of these, so, I guess, if staff don't – you know , if you're a staff and you don't fill them out, then you're not doing – you're not a very effective faculty in the sense of moving residents through.

I Okay, and how would you say that the form helped you with your and your preceptor's goals?

R I guess it's kinda tricky. I mean, in this case it was – like I said, it was kind of more for documentation purposes, so I guess, you know, it's a pretty, efficient way to kinda get through and document that. I think the – when I fill these out with people right there, I guess what's nice about it is really the bottom section of, like, the strength and the weakness piece. I think that's the effective part because it facilitates better feedback (00:03:39) those two things.

I Are you referring to the, sort of, milestones portion or -- ?

R No, I'm referring to the performance piece that you should keep doing and then area of improvement.

I Right, okay.

R I've actually never filled out any of this milestone one.

I Yeah, this is a newer form, yeah.

R Yeah.

I So the reason that we're, sort of, doing these interviews is to get an idea of, sort of, how the new milestones are working, and, sort of, how we can move forward with future forms.

R Okay.

I So I know that your preceptor didn't actually use the milestones in this case because they sort of just clicked the 'all of them were achieved'.

R Yeah.

I But how do you think that the presence of these new milestones would have -- sort of just put yourself in the shoes of your preceptor, and think about how the presence of how these new milestones impacted your preceptor's assessment.

R Well, I think the first thing is I think it makes it much more likely for them to use the 'end' box. You know, if they're trying to decide between – so, for like box 6, 'assessing/diagnosing/00:04:46)', I think, if they were unsure between, you know, the third and the fourth box, I think it would make them more likely to use the 4th because it ends up being less paperwork down the road.

I Right.

R (laughs) Unfortunately. I think that, I guess the benefit of box 7 is the fact that it kinda helps potentially frame and direct specific feedback by giving some nice domains.

I Okay.

R Yeah, I think it also – I guess another thing too is it makes things clear as to what the milestones are when you're considering your answer for box 6.

I Right

R So I guess that's actually a nice piece too.

I Okay, thank you. How would you say that the form interferes with you and your preceptor's goals? Or what are some things that you don't particularly like about the EPA's?

R In general, or this form?

I You can touch on both or either, whichever –

R This form, I think it just looks really long because of those pieces – like, because of box 7 – compared to the other ones.

I Right.

R I think – I find – overall, I find the forms just difficult because they don't really fit into my workflow or my staff's workflow, and so it's a big kinda cultural habit change. I think the benefit is that when you actually get around to doing them, it does help facilitate feedback and you kind know where you're at. This is – mind you, and I think that's maybe speaking for everybody. I'm kind of a person, though, that makes a habit of getting feedback two or three times a week, anyway, every couple of days, and I ask for these types of things and talk about

these types of things, so, like, I haven't found the EPA's have really made much of a difference in terms of the feedback that I get, but I can imagine (00:06:41)

I Because you're already going through this feedback process on your own, sort of, so these are just another thing that you have to complete?

R Yeah.

I Okay.

R But I think – yeah, but I think, from kind of an overall level, it's probably reasonable.

I Okay, so what are your thoughts on the 'save for later' option? And how would you feel if it was removed to force in person feedback?

R I think it'd be a lot more difficult to do EPA's – like, a lot more difficult.

I Yeah, okay.

R Especially when you're on, like, deadline crunches and things, like, you just – I think that, yeah, if everything is cruising along nicely and you have a systematic organized day, which is what, I dunno, 25-30% of the time, you have nothing else to rush to or go to, then I think it works; but, however, most of the time you're reviewing with a fellow over the phone, or your staff is busy, and you've kinda run out of time so you decide to do it later, and the 'save for later' option is very useful in those senses, right, so I think, especially when reviewing cases over the phone with a fellow 'cause you're not gonna have time – 'cause you're not gonna re-discuss it again in the morning, and I might not necessarily remember or remember the details of the case, so it lets the – yeah.

I Okay, thank you. Did your preceptor verbally give you any of the feedback that was documented on this form?

R Not at this time for this one.

I No? Okay.

R Yeah.

I Okay, that's good. Do you have anything else to sorta comment on, on that?

R No, nothing else for that one, yeah.

I Okay. What do you think that your preceptor was noticing when they observed you or reviewed you that led to their score?

R I’m not really sure how to answer that. Could you maybe run me through those words again?

I Sure. Okay, so what do you think was some of the evidence or sort of behavioural, observational evidence that your preceptor would have used when they were observing you or reviewing you that led to the score that they gave you on this EPA?

R Hmm, not really sure. I guess in an ideal world, they’d be kinda looking at in terms of what the milestones were that were mentioned, but I actually don’t really know. I think, generally, probably a lot of it’s (00:09:23). I mean, I had worked there for almost a week by the time we did this one, so I think a lot of that history showed up, probably, like, if it was me doing it, and then just kinda, competence, (00:09:38), how well the patient seems to like you. Those things (00:09:44). I dunno.

I So do you think that they would have used previous or other observations from that day or from previously to sort of inform this score?

R Yeah.

I Okay, so this particular EPA wouldn’t have necessarily have been a super specific reflection of this one case, necessarily?

R Yeah, I mean, I don’t know what kinda margin – I mean , it’s not gonna be like (00:10:10) where they’ve only seen you once. I think it’d be very – yeah, but I think while this score is probably specific to just – or it’s probably written with this in mind, I, just thinking about it myself, probably would inform it based on previous experiences. I think, if I was filling this out for somebody else, that would impact it.

I Okay, thank you. Alright, I think that’s all of the specific questions that I have for you. Let me just double check here ‘cause I kinda went in a weird order. Okay, yeah, that’s all the specific questions that I have, but is there anything else that you’d like to add just about, sort of, EPA’s, in general, or about this particular EPA that we haven’t talked about?

R No, I don’t think so. I think we’ve kind of mentioned them.

I Okay, wonderful. Well, thank you very much for taking the time to chat with me today. This is very helpful.

R Yeah, no problem, thank you.

I	And once the study is complete we're gonna be sending out the gift cards, so just keep an eye out for that.

R	Great, thank you.

I	Okay, thanks so much. Have a good day.

R	You too, bye.

I	Bye.

END 11:21

I Alright, so this is interview code CD1.34.P. Alright, so the first question I have for you, just for context, did you fill out with the resident in person, or did the resident use 'save for later'?

R The resident just emailed it to me after, so she wasn't present at the same time.

I Okay, perfect, for 'save for later'. Do you know why the resident may have used 'save for later' as opposed to doing it in person, in this context?

R I'm not sure, actually. It may have been – I'm trying to think back to the day of the clinic, if we had other – like, this was done in an urgent clinic, and then I think we had other consults in the hospital to go see, so maybe she just felt, like, lack of time, at the time.

I Okay, thank you. Why do you think that the resident chose this case?

R I think she probably chose it because it was a patient coming to the urgent clinic, and so she had to do a full consult and try to come up with a management plan, and she got to spend a fair amount of time with the patient, so perhaps that's why she chose it.

I Okay, so kind of a nice, well rounded case where the resident had sufficient time with the patient.

R Yes.

I Okay, so would you have chosen this case to document?

R I think it was a good one to do, and it was a case that we could see in endocrine in our urgent clinic, so I think, yes, it would be a good case to do.

I Okay, would you say that this encounter was more about direct observation of the resident's skill, or about reviewing their approach to diagnosis and management?

R Reviewing approach to diagnosis and management.

I Okay, great, thank you. So I'd just like you to take a moment to try to sort of relive or visualize the moment that you were reviewing with the resident or filling out this EPA. What were you noticing that led to your score?

R What was a noticing, like, on the – I guess I'm a little bit not certain of the question. So, with the evaluation, what did I notice?

I Yeah, so, I guess what were some of the evidence or observational evidence that you would have used to give this resident the score that you did on this EPA?

R Okay, I think – so I would have been thinking back to when she presented the case to me when we were reviewing, and, you know, listening to her come up with her differential diagnosis, and what she would like to do with regards to treatment and her plan, so mainly, when I was filling out this form, that's what I was – you know, just remembering what her plan was at the time, to be able to fill out, especially the milestone section of the form.

I And did you have any overriding concerns at that point? I noticed that you did you use the milestones. There were a few that weren't sort of indicated as achieved, so what were some of the, I guess, concerns that you had while filling that out?

R Oh, on the ones that are in progress?

I Um hmm.

R And I think – it looks like, when I open it, two of them were in progress or areas to focus on, so the one, 'interpret appropriate investigations based on differential' would just be, probably in this case, thinking of, you know, discussing just all the potential in the differential diagnosis, maybe not just the top, kind of, one, two or three conditions that would cause hypothyroidism, but just having to go through and think of, sort of, the entire list that we'd want to consider, and 'monitor evolution of clinical course', so with that one it was more – I think we were talking about with treatment, with medication, you know, what sort of doses we would be using, or how often we would be monitoring the patient after we started a certain medication, so that was more of a bit of kind of, like, after the initial management, and that would be an area to just – and I wouldn't have, sort of, expected her to know all those answers, so that was more just discussion around that point.

I Okay, perfect. So just sort of off what you just said, did you, verbally, give the resident any feedback that was documented on this form?

R So I told her that she did a really good job just getting all the background history from the patient, and, like, doing a really thorough history, as well as, you know, she did a very thorough exam too, looking for all, sort of, you know, what we may look for in cases of hypothyroidism, and so, at the end of the patient case, I definitely explained that to her, and talked a little bit about what she had chosen for her investigations was really appropriate, and the start of her management, so I was able to give that feedback.

I Okay, so would you say that you, verbally, gave more feedback than what you wrote down? Or would you say you wrote more feedback than what you (00:05:44) with the resident?

R I guess, if you consider all of the milestone boxes, like, then there's probably more on this form than just verbal feedback.

I Okay, great, thank you. Was this your first time working with this resident?

R This was the first time. So this case was not the first time, but it was the first time I had been on service with her for a few days.

I Okay, and would you say that their performance, earlier on, had any impact on your score for this EPA?

R Did their earlier on? Well, I think you always have the –like, whatever experience I had with her from before, I sort of had somewhat of an idea, you know, how she had been doing with other consults or working with the rest of the team, so I don't think it – it didn't change my evaluation of her, but I had just, you know, seen her do other consults as well, which were very similar to the case that we did see, so I don't – I think I based it mostly on the consult that we had done, and I didn't let other things change my evaluation, but, you know , I had – yeah, it's hard to explain. I guess –

I No, I understand what you're saying. You had some background information.

R Yeah, so I tried to – yeah, the feedback, I've tried to give just from that encounter, but I had good experience with other consults from her as well.

I Right, okay, so maybe some of the previous consults sort of just validated the evaluation that you gave her?

R Um hmm, yeah, yeah.

I Okay, great. What were your specific goals when filling out this form?

R My goals were – it would be just to – you know, to make sure if there was anything of a concern, I would have, you know, mentioned that, and also just make sure that she gets acknowledged for the work that she did with the consult, so that's where – she did quite a good job, so that one was mostly achieved for all of them, and just to, yeah, I think the beginning , just to – I didn't write it – probably that would be the other thing – but just to write that it was – this is, like, a really good general consult that would be important for someone to know on endocrine, so she did, and just to show that she did a good job with that.

I Okay, great. So, essentially, communicating a job well done to the resident.

R Um hmm, yeah.

I Okay, excellent. How would you say that the form helped with these goals?

R Sorry, I missed that?

I How would you say that this form helped you with these goals?

R I think it makes it easier because it's – like, you can just check, you know, 'in progress' or 'achieved'. You don't have to specifically write out every aspect, and so I think it saves quite a bit of time.

I Okay, great, and are there any ways that you would say the form interferes with these goals? Or maybe just some things that you don't like about the form, or that you would, sort of, change on future forms?

R I wonder – I guess – the only thing I wonder, with the milestones, if there was – you know, I do like how there's two choices, but then I wonder if, on future forms, if there would be even potentially three areas, or just more choice.

I Right.

R Like, a little bit more of, yeah, variation. So, if someone was doing really well, you could acknowledge that, or if they were sorta at expectation, or then, in the area to focus on more, just to give it – so then it's not sort of, like, black or white – all good or all bad, so maybe a little bit more of a scale, potentially.

I Okay, that's really good feedback, thank you. Okay, so you used the milestones in this case. How well do you think that the milestones help you communicate how the resident is progressing in the program and what they need to do to progress further?

R I think they do – just looking at them here, I think they do a good job identifying what a resident, you know, needs to accomplish when they're seeing a patient, and just looking through – they talk about management, working with other healthcare professionals, so it is fairly complete, without being too long, so I think it has a good combination of touching on the important points without being too long of an evaluation as well, so, off the top of my head, I wouldn't – there's nothing to really add that I can think of, under the milestone section.

I Okay, thank you. How do you feel that filling out this form, overall, helped or hindered teaching and/or patient care?

R Helped or hindered? It was – so, It think, helped, you know, the fact that it was emailed made it east, so that was easy and it was right there. If she had asked me to do it at the same time, I could have definitely done it then, as well, so she might have got even more feedback, potentially, because when I gave her initial feedback, I had never seen this form. So I wonder, if she had asked – or if we

had it open at the same time, she might get more feedback. I know we had a resident from Calgary, and they – in their program, they have to do it with the staff person on one of their – like, on their computer or their phone, so then you do get – you know, you have the chance to sit down and discuss and fill out it, but, you know, (00:12:07) would be hard if it's really busy and that person doesn't have time to fill that out, you may miss opportunities. So, I think, having that opportunity for email is nice, but maybe the resident misses out on some feedback, potentially, just looking at the form. For patent – I don't think it hinders patient care at all because it's done, sort of, after the fact, um hmm.

I	Um hmm, okay, so just along those lines, what are your thoughts on the 'save for later' option, and how would you feel if it was removed in order to force in person feedback?

R	I think it would be – from my perspective, if you were – the 'save for later', so, that is, I think, a nice function, and you can fill it out when you have time. Potentially, the resident will get – if you don't do the 'save for later' and they have to do it right then, the resident may get more feedback, and I – endocrine is not always really, really busy. So, from my perspective, the one to do it at the time would be fine with me – like, not the 'save for later' – so, I'm guessing that most residents so the 'save for later'. Probably it's easier, and you can just email it, and don't really have to talk about it, maybe, at the time, but I would be – yeah, if it wasn't an option, that would be fine with me too. I think they might get more feedback, just on a busy service I could see that possibly being hard to always, you know, have time with the staff person to have it done.

I	Right, that's a good point, thank you. Those are all the specific questions I have for you, but is there anything else that you'd like to add or note about EPA's in general that we haven't talked about today?

R	No, I don't think so. Actually, these are the first – I think this is the first time that I've seen these ones while on service. Maybe they were a little bit different before. So, I thought, filling it out, I didn't think it was hard at all, and the questions were good, so I – yeah, I thought, yeah, overall, the form was quite well done.

I	Okay, thank you so much.

R	You're welcome.

I	I really appreciate you taking the time to give some feedback and chat with me today. These interviews are very helpful just for informing future EPA forms, just to provide residents with better feedback, so I really appreciate it.

R	Okay, no problem.

I Thank you very much.

R Thanks, [name].

I Yeah, you’re welcome. Enjoy the rest of your day.

R You too, bye bye

I Bye

END 14:51
