# Supplementary material for: Misbehavior or misalignment? Examining the drift towards bureaucratic box-ticking in Competency-Based Medical Education
Source: PLoS One. 2025 Jan 2;20(1):e0313021. doi: 10.1371/journal.pone.0313021 (PMC11694975; doi:10.1371/journal.pone.0313021)
Supplement: Attachment — Submitted filename: Response to Reviewers.docx [file pone.0313021.s003.docx]

**Response to Reviewers**

**Effect of Detailed OSCE Score Reporting on Learning and Anxiety in Medical School**

Dear Prof. Dr. Yaser Mohammed Al-Worafi

My thanks to you and the reviewers for your response to our manuscript titled, “Misbehavior or misalignment? Examining the drift towards bureaucratic box-ticking in Competency-Based Medical Education”. We are pleased the paper was received favourable comments, but also pleased for the helpful comments. We have revised the manuscript based on the reviewer’s valuable suggestions and comments. We address each of the reviewer’s comments below (the location of revisions in the manuscript are noted).

**Journal Requirements**

*1. When submitting your revision, we need you to address these additional requirements.*

*Please ensure that your manuscript meets PLOS ONE's style requirements, including those for file naming. The PLOS ONE style*

*templates can be found at*

*https://journals.plos.org/plosone/s/file?id=wjVg/PLOSOne_formatting_sample_main_body.pdf and*

*https://journals.plos.org/plosone/s/file?id=ba62/PLOSOne_formatting_sample_title_authors_affiliations.pdf.*

Thank you for pointing out this template. I am not sure how we missed this and apologize. We have made all of the changes to match the formatting correctly, but if we missed anything, please let us know.

*2. Thank you for stating the following financial disclosure:*

*[This work was supported by the University of Alberta’s Teaching and Learning Enhancement Fund with VJD as Principal Investigator*

*(https://www.ualberta.ca/provost/funding/grants/tlef/index.html). The funder had no say in anything related to study design, data collect/analysis, or writing the manuscript.].*

*Please state what role the funders took in the study. If the funders had no role, please state: ""The funders had no role in study design, data collection and analysis, decision to publish, or preparation of the manuscript.""*

*If this statement is not correct you must amend it as needed.*

*Please include this amended Role of Funder statement in your cover letter; we will change the online submission form on your behalf.*

We have added this to the cover letter.

*3. Thank you for stating the following in the Acknowledgments Section of your manuscript:*

*[Funding/Support: This work was supported by the University of Alberta’s Teaching and Learning Enhancement Fund with Dr. Daniels as Principal Investigator]*

*We note that you have provided funding information that is not currently declared in your Funding Statement. However, funding information*

*should not appear in the Acknowledgments section or other areas of your manuscript. We will only publish funding information present in*

*the Funding Statement section of the online submission form.*

*Please remove any funding-related text from the manuscript and let us know how you would like to update your Funding Statement.*

*Currently, your Funding Statement reads as follows:*

*[This work was supported by the University of Alberta’s Teaching and Learning Enhancement Fund with VJD as Principal Investigator*

*(https://www.ualberta.ca/provost/funding/grants/tlef/index.html). The funder had no say in anything related to study design, data collect/analysis, or writing the manuscript.].*

*Please include your amended statements within your cover letter; we will change the online submission form on your behalf.*

We have removed this from the manuscript and included it only in the cover letter.

*4. In the online submission form, you indicated that [The full transcripts are stored on the hard drive of the corresponding authors computer.*

*There are no limitations to sharing the transcripts.].*

*All PLOS journals now require all data underlying the findings described in their manuscript to be freely available to other researchers,*

*either 1. In a public repository, 2. Within the manuscript itself, or 3. Uploaded as supplementary information.*

*This policy applies to all data except where public deposition would breach compliance with the protocol approved by your research ethics*

*board. If your data cannot be made publicly available for ethical or legal reasons (e.g., public availability would compromise patient*

*privacy), please explain your reasons on resubmission and your exemption request will be escalated for approval.*

We have revisited our consent form and participants did not explicitly consent to having their full transcript in a data repository. We have uploaded them as supplementary information as requested. If future researchers wish to access the transcript and have received ethics approval from their institution, these can be shared.

*5. Your ethics statement should only appear in the Methods section of your manuscript. If your ethics statement is written in any section*

*besides the Methods, please delete it from any other section.*

We have kept the Ethics statement that is at the end of the Methods section and remove the other mention of ethics from the Acknowledgements section.

*6. Please include captions for your Supporting Information files at the end of your manuscript, and update any in-text citations to match*

*accordingly. Please see our Supporting Information guidelines for more information: http://journals.plos.org/plosone/s/supporting-*

*information.*

We have corrected the mentions of figures and supplementary materials in the document and renamed those files to align with the convention your journal is using.

**Reviewer comments**

**Reviewer #1:**

*Thank you for asking me to review this study of CBME examining the University of Alberta Internal Medicine program.*

*I enjoyed reading the paper and I think it sheds a light on important and under-appreciated “downsides” of CBME and EPAs.*

Thank you for your positive comment!

*I have some comments:*

*Major comment: much of the paper focuses on the terms “bureaucracy” and “bureaucratic”, implying these are negative concepts or negative developments. I think most readers will have an intrinsic dis-like of bureaucracy, but for academic purposes, this needs to be defined and better explained. What do the authors mean by bureaucracy? The dictionary definition of “bureaucracy” is: “a system of government in which most of the important decisions are made by state officials rather than by elected representatives.” However, this is not the implied definition I read from the paper, where the term seems to be used more to denote that many CBME activities have changed from authentic, enthusiastically undertaken educational exercises towards mindless, meaningless “checklist” activities.*

Thank you for pointing this out. We used the word “bureaucratic” as we are quoting from our second reference Schut et al. But we see that this may not be 100% clear so we have throughout the manuscript elaborated such as in the abstract we make it clear we mean more of a focus on the administrative aspects.

*Major comment: “Further consideration is needed for suggestions such as separating formative and summative assessments in the workplace5,14 and moving away from the notion that every feedback moment could be documented and used as data for assessment decisions.23” Some caution is needed here. The authors have spent much of the paper (quite correctly) demonstrating the heavy, heavy burden of checklists and paperwork being placed on trainees and faculty by mindless implementation of CBME. Any suggestion to fix this needs to reduce paperwork and checklists, not increase it. So if a suggestion like separating formative and summative assessments is made, it needs to go hand-in-hand with how such as suggestion could DECREASE paperwork, not increase it. Trust me. Otherwise someone of low intelligence will read this and decide to double the EPAs in their program into formative and summative. It’s sad but true.*

Thank you for pointing out how this section could be interpreted. We have revised that section to read as follows:

“Further consideration is needed for suggestions such as separating formative and summative assessments in the workplace [5,14], reducing the requirement these formative moments are captured so as to move away from the notion that every feedback moment could be documented and used as data for assessment decisions [23]. This would reduce the current burden of assessment on all involved.

*Minor comment: “Others make the argument that reforming trainee-teacher relationships toward an ‘Educational Alliance’ model may overcome artificiality from administrative mandates and realign assessment for learning goals.” I’m not sure “reform” is the right word here – it looks to me like the preceptors and trainees interviewed in this study are already working on an “educational alliance” model even if they don’t explicitly have Telio’s framework in mind (i.e. residents feel they deserve an EPA after working overnight at the hospital, preceptors oblige, etc)*

Thank you for noting this. We agree our residens and preceptors had some element of this but not as much as we like. So we have added the following sentence. “We saw some of this with how preceptors and residents used EPAs transactionally, but we feel a more explicit alliance would move us close to these goals.”

*Major comment: what happens when an EPA is largely “not achieved” or the trainee clearly requires remediation? The overall sense that I get from reading this paper is that all the interactions recorded in the paper were largely “fine” – i.e. the resident did “well enough” on the EPA and the preceptors was spared from having the “remediation” talk, and thus much of the focus was on the bureaucratic need to “get it done”. However, one of the original purposes of CBME and EPAs was to make it “easier” to give residents feedback on where they need to improve or where they are not meeting expected standards. One would imagine that, if CBME is being implemented as intended, of those 16 residents and 27 preceptors, at least one of those interactions should have been a “does not meet expectations” type of encounter. If so, please flesh this out some more in the results/Discussion. If there were no “negative” or “failing” EPAs….why? CBME/EPAs were initially sold to Canadian programs as a way of overcoming the ”failure to fail” problem in residency program. Maybe I’m reading the paper wrong and there were some substandard EPAs filled out, but if so I think that aspect needs more attention.*

Thank you for pointing this out. One our four point scale, a resident was only considered a concern if they were scored at the lowest category indicating they did not have even a basic approach to the case. All other scores were considered acceptable and the focus was giving feedback to move them to the entrustable level. To clarify this, I added the following sentence to the methods:

“A resident was only flagged for concerns if they were scored in the lowest category “Does not have a basic approach...” which was an uncommon occurrence.”

It was not part of our ethics application at the time to pull the percentage forms that were scored at the various four categories so unfortunately we cannot expand further.

*[limitations section: “First, our study included interviewing only the participants who had submitted an EPA assessment as we are unable to analyze data from participants who declined to submit assessments.”…. what could be missing here?] Just some food for thought.*

Thank you. We did mention this was a limitation and we felt we could not make too many conjectures as to what those who did not submit may have felt. That said, as we outline lower down in response to another reviewer, we have added that 80-90% of forms that are started, are submitted.

**Reviewer #2: Overall**

*-The authors present a qualitative study looking at trainee and faculty perspectives related to assessment processes following implementation of a pilot version of competency-based medical education in the Internal Medicine Program at the University of Alberta.*

*-Limitations to this study include the age of the data (2018) and the pilot nature of the implementation of CBME at this point (national deployment in 2019). It is unclear to what degree some of the challenges identified in this study are due to an early transitional period with limited familiarity with CBME (instructional video was the only preparation described). By this point (2024), programs have had much more experience with the CBME theory and tools and have made many adjustments and optimizations. The Royal College itself is preparing for revisions to CBD (CBD 2.0) which promises more flexibility.*

We thank the reviewer for these comments. Yes, it would have been ideal to have this paper out for review much earlier, but due to unforeseen circumstances impacting the first author and senior author, this was not possible.

That said, two of the authors are frontline clinical teachers who frequently do EPAs and support competency based programs and 6 years later the same tensions are felt today and have not radically changed over time. In fact the situation is so dire as we have had many programs put on “intent to withdraw accreditation” at a national level due to issues with CBD and EPAs. While this is well known nationally, this is not specifically public knowledge for us to cite in our paper.

So we feel this paper still makes a worth contribution to the literature many of our findings are not described in other papers to date.

We apologize if our faculty development was not clear. We kept the comments about it short as we have previously published our faculty development approach (which is cited in the paper) and it involved several presentations with every division in our department in addition to grand rounds and educational videos. We expanded this section so it reads now as: “Program leads oriented faculty and residents to CBME and EPAs through presentations to each division in the department, and at grand rounds, and through online videos and guidance documents on our local Internal Medicine website.”

Lastly with respect to CBD 2.0, I am glad to hear this reviewer is well versed in the Canadian context as that situates them ideally to review our paper. Coincidentally around the same date we received this revise and resubmit from PLOS ONE, we received notice about CBD 2.0 and while we agree with the reviewers there is some promising news on the horizon, it is clear EPAs are not going away and their multiple intended uses of formative and summative and checklist to prove to the public are not changing dramatically. So while the impact of our findings may change over the next 5 years depending on which way the Royal College goes, we still feel it has importance today.

*-Challenges to CBD implementation in Internal Medicine, which is more longitudinal and less amenable to evaluation of discrete tasks/procedures (like Surgery) have been the topic of discussion across the country and this manuscript has the capacity to contribute to that literature, although the manuscript itself does not address the nuanced specialty-specific challenges in detail. It is possible that such details are not evident due to the early phase of implementation at time of data collection. The interviews themselves were also relatively short (4-16 minutes).*

We agree with the reviewer that this work is a lens into only one program and cannot be used to compare with other programs.

With respect to the length of interviews, we should have explained with more statistics than a range. In addition, while 10 min seems short for most interviews, we only had three focused questions. We have modified this part of the results section to say, “Interviews covering the three main questions to participants (see S1 File) lasted between four and sixteen minutes (mean and median 10 min, 37 sec).”

*Abstract*

*-"This study sought to examine resident and faculty experiences with implemented EPA processes to elucidate what leads them toward a ‘tick-box’ approach.": The purpose seems to draw conclusions as to the outcome of participant experiences prior to description of results or discussion.*

We seem how this statement implies we knew the outcome. What we meant was that the “tick box” approach was found in other literature as well and our study was to elucidate that. So that sentence from the abstract now reads, “This study sought to examine resident and faculty experiences with implemented EPA processes to elucidate what leads them toward a ‘tick-box’ approach that has been described in the literature”

*Materials and Methods*

*-Data were collected from April 2018 - July 2018. Why are the data so old? Is this study still contemporaneous given that institutions and programs have had much more time since the study data was collected to gain experience with CBME to better understand the theoretical and practical underpinnings and implement change. This study data was very much collected in a time of transition.*

We addressed the reviewer’s concerns higher up but in short, due to unforeseen circumstances with the first and senior author, the publication of this study has been delayed. That said, as explained higher up, the concerns are still present to this day and we feel our study still adds much to the literature.

*-CBD deployment for Internal Medicine is described as July 2019. Given that the Internal Medicine program was running a pilot curriculum at the time of data collection, it would be important to clarify if there are any differences between the pilot curriculum and curriculum post deployment of CBD in Internal Medicine nationwide (e.g., number or distribution of rotations, length of stages, assessment forms).*

Thank you for this comment as it is quite fair and appropriate. We failed to describe that our curriculum had changed to meet the requirements of the upcoming CBD deployment as nationally it was originally supposed to be implemented in 2017 but was delayed two years. That paragraph now reads as follows:

“Nationwide CBME implementation for Internal Medicine in Canada took place in July 2019. Our institution started a pilot EPA program in 2016 followed by a full launch in July 2017. The curriculum of the residency program had already been adjusted to be compliant with the national standards with the focus now on assessment.”

*-Assessment tool: "The RCPSC’s approach was that if a resident was deemed entrustable for a task, the milestones were optional, and we modeled our form similarly." Please provide a reference for this statement. It may be worthwhile, if data is published and available, comparing this to the implementation of EPAs at other institutions. Are milestones optional in other Internal Medicine programs? Are there other differences in the forms between CBD implementations (e.g., N/A option for milestones that do not apply)? It seems that boiling down an assessment to just the entrustment score is the source of some of the issues raised in this paper and also turns what is meant to be an objective assessment of a particular skill into a more "gestalt" evaluation. Providing some brief details of the Internal Medicine curriculum specifically may also be helpful for context. Are there other contextual fields which could help frame the assessment data (e.g., nature of encounter inpatient/outpatient/emergency; clinical rotation; etc.) and are these collected on the forms?*

We thank the reviewer for some excellent thoughts here. We will tackle each of them in order. With respect to our modeling our form like the RCPSC approach, this was based on viewing their forms in their assessment system which is now defunct. But this approach did continue as our PGME office insisted we take this approach as we had some systems using the Royal College’s system and some using a locally developed system. We do not feel this information is required for the paper as we do not have a citation we can add so we hope the reviewer is understanding of this.

Second, our understanding is the milestones do not need to be mandatory on the forms but that most institutions made them mandatory. This is just based on national conversations and we are not aware of published data to cite for this.

With respect to the curriculum and impact of specific rotations, we do not track which rotation an EPA was done on but to address the reviewer’s question, we have added the following to the Participants section:

“EPAs would be primarily done in the emergency department for first year residents when they were seeing emergency department patients in consultation. For second year residents, it would be a mix of emergency department, inpatient consultation to other services, and ambulatory experiences that led to an EPA observation.”

*-It could be helpful if the methodology for developing the semi-structured interview guides was more thoroughly described.*

We have expanded this section in the Data collection but would be happy to add more if needed:

“The semi-structured interview guides (S1 File) were developed by two of the authors (AG, VJD). Both AG and VJD have experience in qualitative methods. The interview guide was modified after each cycle of data collection and analysis to ensure participants focused on how the form and process helped them meet their goal for the EPA encounter. The final version focused on three aspects of the encounter, 1) what the preceptors was noticing / observing, 2) what information the preceptor used to make a judgment, and 3) what the preceptor’s goals were when interacting with the assessment form (see S1 File).”

*-Interviews were conducted by telephone by a research assistant. Is the research assistant one of the authors?*

No they were not and we have updated this in this sentence in the methods: “They contacted a research assistant who was not one of the authors to schedule a phone interview within a few days of the EPA submission.”

*Results*

*-Only 16/67 eligible residents participated, which is a limitation and could potential introduce some selection bias*

We agree it would have been good to get more residents to balance resident numbers with faculty but we felt the 43 interviews were sufficient to be confident in our themes. That said we have added this to the limitations section.

*-Interviews were relatively short (four to sixteen minutes)-was there sufficient data to probe participants perceptions on CBME or their understanding of the tools to an adequately nuanced degree?*

The focus of our study was not their understanding / perception of CBME but of the process of EPA acquisition. While some interviews which were quite short, we felt most gave us the information we needed.

*-It seems there was limited training of faculty (explanatory video) on the new curriculum; were faculty adequately familiar with the CBME principles and tools during this pilot stage?*

We have addressed this comment higher up and expanded the section on faculty development.

*-Feeling obligated: Based on the thematic analysis, it seems that both trainees and faculty at this stage in implementation were focused almost entirely on number of entrustments. Did trainees and faculty understand the role of assessments to capture real-time data at a point in time?*

Yes, while this project was not aimed at evaluating if trainees and faculty understood the point of real-time workplace based assessment data, it was our experience that residents and faculty understood the intended purpose of this independent of our study.

*Were assessments used to document both positive and negative feedback to guide resident progression? Were there way to provide more granular data about individual encounters - could preceptors click N/A for certain milestones and/or were there other ways to put a particular assessment into context ("in some instances, this meant that preceptors completed an EPA even though the case was a poor fit")?*

Yes preceptors could skip any milestone that did not apply. We realize this was not clear in our paper so we have modified the Assessment tool section to state, “The RCPSC’s approach was that if a resident was deemed entrustable for a task, the milestones were optional, and we used similar language to the RCPSC approach. We also made the milestones optional but strongly encouraged preceptors to fill the applicable ones if a resident was not entrustable. Preceptors could choose to skip any that did not apply.”

*Were faculty free to decline assessments? Could faculty trigger assessments themselves? Were faculty sufficiently well versed in the necessary EPAs to know when it might be appropriate to trigger a particular EPA?*

We realize we did not address these important questions so we have added the following:

“Our system was set up so that forms could only be started by residents to put the onus on residents to ask for observations. We encourage residents to fill in the form with the preceptor, though there is an option to save it for later. Residents can trigger an email reminder to the preceptor if they have not filled it in after 72 hours but there is nothing that forces the preceptor to fill them in. Institutionally, both during the time of the study and since then, forms that are started are filled out 80-90% of time.”

*The authors also describe EPAs as being both "formative and summative"; however, there is a missing discussion that each assessment is an evaluation of a specific task at a specific point in time. There is little to no acknowledgement that the summative portion of this is partly accomplished through aggregation of data which is reviewed at the program or Competence Committee level.*

We agree with the reviewer we did not add in anything about competence committees and we have added in some references to them.

*-A prescriptive process meets the volatility of the workplace. "some preceptors adapted by “half-heartedly answer[ing] all these specific questions that did not apply.”" As per above, was there an N/A option for fields that did not apply? This would probably provide more accurate contextual information that having preceptors inaccurately evaluate a component that did not apply or was not observed in an individual encounter.*

As mentioned above, preceptors could skip milestones, but some still chose to just fill them all in.

*-"Many preceptors prioritized communicating with the resident which sometimes led to more emphasis on the narrative free-text sections with less attention to the entrustment scale": This is an important component of any assessment data, and there is much work currently being done to help better sort through the reams of quantitative and qualitative (narrative) data that is being generated through CBME.*

Yes we agree, but hopefully it came across that by downgrading the importance of the entrustment scale, that preceptor was declaring their focus was formative over summative.

*-"So, residents doubted the credibility of the assessment, wondering if the assessor was “giving the highest score to avoid doing that, or did I truly do a good job?”": Again, it would be interesting to know if this function (no questions answered if entrustment granted) is common amongst Internal Medicine programs in the implementation of CBD. It seems that this implementation would favour a "gestalt" evaluation which may have less actionable feedback.*

What we were trying to highlight here is that a model in which filling in a long list of milestones is dependent on if you think they are not yet entrustable swayed some preceptors to say a resident is entrustable to get out of filling in the rest of the form. If the reviewer feels that was not clear, we would be happy to revise things further.

*-Evidence of feedback or proof of learning: "Similarly, although they acknowledged that the scoring was supposed to be case- and task-specific, some preceptors prioritized formative usefulness over summative objectivity by arguing that “from a development perspective, I think it’s better if I can give them some meaningful feedback based on two weeks’ performance”: This statement again makes it unclear whether the faculty understood the tools at this pilot point in implementation; perhaps more detailed frequent feedback could have been provided rather than collapsing multiple encounters into one assessment. This kind of batched assessment might be more appropriate at a later stage of training?*

Yes we completely agree with the reviewer and this is why this was problematic. Preceptors were explained how EPAs are supposed to be task specific and yet disregarded this to meet their own goals. We have expanded the faculty development portion to better emphasize this as follows:

“A key aspect emphasized was that EPA observations were about a specific task and that these complement more gestalt assessment that already existed in the program.”

*-“it’s not fair to say that he didn’t achieve anything when he did really well for his level on this case.”: Does this statement suggest a miscalibration of the EPA to the stage of training? In theory, the EPAs are stage specific, and the assessment should therefore be more objective to the specific task (which should be achievable at that stage of training).*

Yes as we pointed out in this section was that case selection was part of the problem (and anecdotally is still a problem).

*-One message, two audiences: Again, since each stage has stage-specific EPAs, it seems that based on the text, faculty at this point may have required further coaching as to how to implement these stage-specific EPAs rather than qualitatively and subjectively scaling assessments to "level of training. There is also some discussion about faculty not being sent an EPA for a task that the resident did well on. Did faculty have the capacity to trigger EPA assessments?*

As mentioned they did not have the capacity to trigger EPAs at the time of the study. They do now, though we do not advertise it as we still want residents to take the lead. But we are at a stage of implementation in which that may shift.

*Discussion*

*-The authors do acknowledge the need for knowledge and buy-in of new processes. However, they indicate the use of "workarounds" as an indication that this is not an adequate explanation for the perceptions and behaviours identified in this study. However, there are numerous indications in the text that the data was collected early in implementation and there may have been some gaps in the application of CBME based on the familiarity of trainees and faculty. Certainly, the two are not mutually exclusive and both a "gamesmanship" of learner optimization to meet the requirements of their training program and a more practical aspect of feedback for learning calibration can both be true.*

While it may be that our findings could have been due to only a transition period, it has been our experience to date this behaviour continues. One of the authors sits on a competence committee for a subspecialty program and another author oversees CBME implementation and both have witnessed this ongoing behaviour of workarounds. Without reproducing the study today, it would be hard to add much to the paper to endorse that this continues to be a problem.

*-It may be worth acknowledging whether the authors have considered any potential bias due to their own training in the traditional time-based curriculum (could there be any impact on formulation of research questions? data collection?)*.

VJD was trained in a time-based model before EPAs, while ACS was in training during the launch of EPA assessments for her subspecialty training; they considered how these differing experiences may impact their interpretation of the findings.
